# Source-Sink Dynamics in Field-Grown Durum Wheat Under Contrasting Nitrogen Supplies: Key Role of Non-Foliar Organs During Grain Filling

**DOI:** 10.3389/fpls.2022.869680

**Published:** 2022-04-29

**Authors:** Raquel Martínez-Peña, Armin Schlereth, Melanie Höhne, Beatrice Encke, Rosa Morcuende, María Teresa Nieto-Taladriz, José Luis Araus, Nieves Aparicio, Rubén Vicente

**Affiliations:** ^1^Group of Cereals, Section of Herbaceous, Instituto Tecnológico Agrario de Castilla y León (ITACyL), Junta de Castilla y León, Valladolid, Spain; ^2^Max Planck Institute of Molecular Plant Physiology, Potsdam, Germany; ^3^Institute of Natural Resources and Agrobiology of Salamanca (IRNASA), Consejo Superior de Investigaciones Científicas (CSIC), Salamanca, Spain; ^4^Instituto Nacional de Investigación y Tecnología Agraria y Alimentaria (INIA), Madrid, Spain; ^5^Integrative Crop Ecophysiology Group, Section of Plant Physiology, Faculty of Biology, University of Barcelona, Barcelona, Spain; ^6^Instituto de Tecnologia Química e Biológica António Xavier, Universidade Nova de Lisboa (ITQB NOVA), Plant Ecophysiology and Metabolism Group, Oeiras, Portugal

**Keywords:** durum wheat, ear, genotypic variability, metabolism, nitrogen, phenotyping, grain yield

## Abstract

The integration of high-throughput phenotyping and metabolic approaches is a suitable strategy to study the genotype-by-environment interaction and identify novel traits for crop improvement from canopy to an organ level. Our aims were to study the phenotypic and metabolic traits that are related to grain yield and quality at canopy and organ levels, with a special focus on source-sink coordination under contrasting N supplies. Four modern durum wheat varieties with contrasting grain yield were grown in field conditions under two N fertilization levels in north-eastern Spain. We evaluated canopy vegetation indices taken throughout the growing season, physiological and metabolic traits in different photosynthetic organs (flag leaf blade, sheath, peduncle, awn, glume, and lemma) at anthesis and mid-grain filling stages, and agronomic and grain quality traits at harvest. Low N supply triggered an imbalance of C and N coordination at the whole plant level, leading to a reduction of grain yield and nutrient composition. The activities of key enzymes in C and N metabolism as well as the levels of photoassimilates showed that each organ plays an important role during grain filling, some with a higher photosynthetic capacity, others for nutrient storage for later stages of grain filling, or N assimilation and recycling. Interestingly, the enzyme activities and sucrose content of the ear organs were positively associated with grain yield and quality, suggesting, together with the regression models using isotope signatures, the potential contribution of these organs during grain filling. This study highlights the use of holistic approaches to the identification of novel targets to improve grain yield and quality in C_3_ cereals and the key role of non-foliar organs at late-growth stages.

## Introduction

Global crop production needs to double by 2050 to meet the rising population demands, nutritional requirements, and increasing biofuels consumption (Ray et al., 2013). Boosting crop yields to meet these rising demands is the ideal solution to meet this goal. Durum wheat is an economically and culturally important crops widely cultivated in the Mediterranean basin, used mainly to produce pasta and other non-baked products, as bulgur and couscous. It provides 18% of the daily intake of calories and 20% of proteins in the human diet (Royo et al., 2017). Global durum wheat production achieved around 38–40 million tons (~5% of total wheat production) and is concentrated in Mediterranean areas, being the European Union, North Africa, and Middle East countries the primary producers and consumers (Beres et al., 2020; Xynias et al., 2020). In Spain, durum wheat was grown in 266,644 ha, producing 704,086 tons, which represented 14% and 12% of the total wheat area and production in the country, respectively (Ministry of Agriculture, Fisheries and Food of Spain, 2019; www.mapa.gob.es).

To meet future food demands, a worldwide crop yield increase of 2.4% per year is required (Ray et al., 2013), although the genetic advancement in the last decade for durum wheat has been much lower or even stagnated in different Mediterranean agro-environments (Chairi et al., 2018; Del Pozo et al., 2019). Moreover, climate change will increase the vulnerability of durum wheat production to the impact of abiotic stresses in the Mediterranean countries, where a rise in mean temperatures and lower precipitations is predicted (IPCC, 2013), which will limit grain number and further grain filling by inhibiting C fixation and N assimilation (Vicente et al., 2015, 2018b, 2019a; Medina et al., 2016). Therefore, it is of strategic importance for Mediterranean agriculture to develop new varieties with more significant production potential, better adaptation to increasingly adverse environmental conditions, and better grain quality (GQ). However, genetic advance is constrained by the lack of exploring the available genetic diversity in terms of traits to select, high-throughput phenotyping techniques to implement, and the better understanding of key molecular mechanisms behind crop adaptation to stress conditions.

Firstly, the development and implementation of high-throughput phenotyping approaches are necessary for the provision of information about the genotype-by-environment interaction and selection criteria for breeding programs, but further efforts are also needed for selection toward adaptation to abiotic stresses (Kefauver et al., 2017; Vicente et al., 2019b; Prey and Schmidhalter, 2020). Secondly, most of the breeding efforts during the last century were focused on improving wheat yields. Selection toward high-yielding cultivars has been done using a few agronomical and physiological traits (Del Pozo et al., 2016). However, other factors, such as the nutritional grain quality and pasting behavior, relevant for human diet, and industrial processing, were considered secondary (Sanchez-Garcia et al., 2015). In the Mediterranean basin, where yield gaps are high and environmental stresses may prevent progress, selection for increased adaptation to abiotic stresses is a potential strategy to support future yield progress. However, its complexity has been a challenge for crop improvement as the study of local adaptation requires multidisciplinary studies with multiple environments. Hence, the success in future breeding strategies may reside in novel holistic approaches integrating agronomy, field phenotyping, metabolism, and molecular biology in extensive wheat collections to identify attributes controlling complex traits, i.e., grain yield (GY) and GQ under various stresses (Araus et al., 2021b).

Canopy photosynthesis, understood as the photosynthesis of foliar and non-foliar photosynthetic green organs, is a key target for improving crop yield and resilience (Sanchez-Bragado et al., 2020b; Araus et al., 2021b). Traditionally, it has been thought that the key contributor for canopy photosynthesis during the grain filling stage was the flag leaf blade (the last fully developed leaf in cereals), while the reserves stored in the stems before anthesis were also involved providing C and other nutrients (Sanchez-Bragado et al., 2020b). However, it has been recently shown experimentally that the photosynthesis of non-foliar organs, including the whole ear, may significantly contribute to canopy photosynthesis and, then, GY (Gámez et al., 2020; Molero and Reynolds, 2020; Shokat et al., 2020; Araus et al., 2021b). This can be particularly relevant under abiotic stresses, e.g., water stress, but may also contribute to GY under good agronomical conditions (Sanchez-Bragado et al., 2014a,b, 2016). However, the methodologies for studying the contribution of ears or other non-foliar organs to GY are frequently intrusive or cause compensatory effects (Sanchez-Bragado et al., 2016; Rivera-Amado et al., 2020). Ears exhibit higher tolerance to limiting stress conditions compared to leaves, with minor or even insignificant negative impacts on photosynthetic and electron transport rates, and N and water status, including a higher content and expression of primary metabolism intermediates and genes, respectively (Sanchez-Bragado et al., 2014b, 2017; Vicente et al., 2018b; Vergara-Diaz et al., 2020b; Tambussi et al., 2021). Ears are the latest photosynthetic organ to develop in wheat, therefore being the youngest organ and, potentially, the last to show symptoms of senescence during the grain-filling period (Vicente et al., 2018b). Moreover, ears are, by nature, more exposed to direct sun rays and less exposed to shadows due to their apical position, with a smaller physical distance to the grain than any other organ. Awns, which are not always present in wheat varieties, seem to be a major contributor to ear photosynthesis (Sanchez-Bragado et al., 2020a). Ear bracts (glumes and lemmas) have closer contact with grains and, moreover, access to the respired CO_2_ released by grains, which could be relevant in possible refixation of CO_2_ (Sanchez-Bragado et al., 2020b). Regarding sheaths and peduncles, they have been associated with storage and nutrient transport functions (Scofield et al., 2009; Cimini et al., 2015). Overall, the precise pathways associated with the metabolism operating in the ears, and other non-foliar organs are still poorly understood.

N metabolism is a key factor in plant growth, with a crucial impact on GY and GQ traits, such as protein content, dough quality, and processing characteristics (Zörb et al., 2018; Wang et al., 2021). It is relevant for the wheat research to understand the N assimilation and remobilization taking place in the different green organs, especially during grain filling. For example, under water stress, the ear bracts showed active biosynthesis of organic and amino acids, which was not observed in flag leaves, thanks to a coordination between C and N metabolism, including N assimilation, photorespiratory N cycle, and tricarboxylic acid (TCA) cycle (Vergara-Diaz et al., 2020b). A preliminary study analyzing the N content and isotope composition in different parts suggested that the potential contribution of the ear, providing N to the growing grains, was around 42% (Sanchez-Bragado et al., 2017), which makes this topic of interest for addressing new avenues for crop improvement.

This study aims to perform a holistic approach, integrating agronomic, physiological, and biochemical traits to identify novel components involved in the control of complex traits in response to different N levels as a selection criterion for breeding programs. Our specific objectives are to understand (i) the phenotypic traits that are related to GY and GQ at canopy and organ levels, (ii) the potential contribution of foliar and non-foliar photosynthetic organs to developing grains and (iii) how N availability and genotypic variability modulate such factors. We evaluated a wide range of canopy vegetation indices, physiological, nutritional, and metabolic traits in green organs (flag leaf blade, sheath, peduncle, awn, glume, and lemma), and agronomic and GQ traits.

## Materials and Methods

### Plant Material and Experimental Design

**Figure 1:**
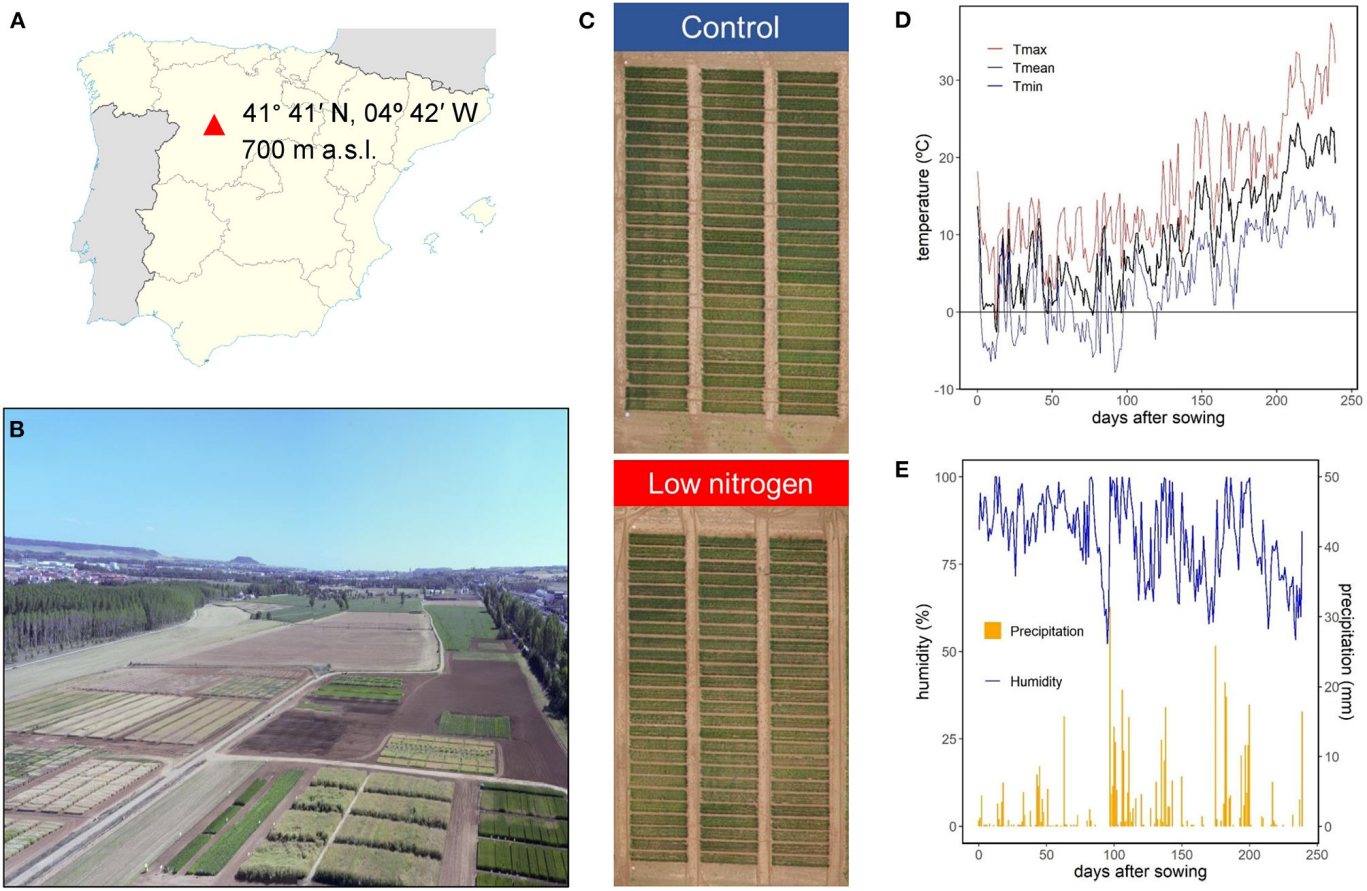
Location **(A)** and aerial images **(B,C)** of the field trials, and daily mean (T mean), maximum (T max), and minimum (T min) temperatures **(D)**, relative humidity, and precipitation **(E)** during the crop season from November 23, 2017 to July 20, 2018 **(D)**.

Four modern durum wheat [*Triticumturgidum* L. ssp. *durum* (Desf.)] varieties, widely used in the Mediterranean region, in particular in Spain, were grown during the crop season of 2017/2018, with contrasting agronomic components (e.g., yield): Euroduro, Don Ricardo, Kiko Nick, and Haristide, released in 2007, 2008, 2009, and 2015, respectively. Haristide was considered as a high-yielding variety, together with Euroduro, while Don Ricardo and, especially, Kiko Nick, were considered as low-yielding varieties (see details below). The field trials were carried out in the experimental station of Zamadueñas from the Instituto Tecnológico Agrario de Castilla y León (ITACyL), located in Valladolid, Spain (41° 41′ N, 04° 42′ W, 700 m above the sea level; Figures 1A–C). The climate is continental Mediterranean, and the soil is xerofluvent with sandy slit texture; the upper 0.30 m having 11.9 g kg^−1^ organic matter, 33 g kg^−1^ carbonate, 0.68 g kg^−1^ N, pH of 8.4, and electric conductivity of 0.135 dS m^−1^. Meteorological data were collected at an automated meteorological station located in the experimental station. During the whole crop season, the average, maximum, and minimum temperatures were 9.8, 15.8, and 4.5°C, respectively, with 83% humidity and 476 mm of accumulated precipitation (Figures 1D,E). The sowing, at a rate of 250 seeds m^−2^, and the harvest, about 20–25 days after reaching physiological maturity, were performed on November 23, 2017 and July 20, 2018, respectively. Before sowing, the field trial had received a basal application of 300 kg ha^−1^ of 8-15-15 NPK fertilizer on November 22, 2017. Then, two N regimes were applied: control N supply, following the standard agronomic practices in this area, and a lower N supply (termed “low N” hereinafter; Figure 1C). The control N treatment was dressed with N applied at the beginning of tillering (February 20, 2018) and jointing (April 14 2018) using a dose of 150 kg ha^−1^ of calcium ammonium nitrate (CAN, 27%) and 150 kg ha^−1^ of ammonium nitrosulfate (ASN, 26%). The low N treatment was not fertilized, relying exclusively on the N available in the soil before sowing. Therefore, the control and low N treatments received a total of 105 and 24 N kg ha^−1^, respectively. The trial was performed in an alpha-lattice design, with three replications per variety and N supply. The size of each plot was 6-m long per 1.5-m wide (9 m^2^), with six rows and a space between them of 0.25 m. Weeds, insect pests, and diseases were controlled by applying of the recommenced agrochemicals to avoid yield limitations. Agronomic and GQ traits were evaluated at harvest and the phenology monitored throughout the growth cycle using the Zadoks scale (Zadoks et al., 1974). Ground-phenotypingwas performed during the crop cycle at the canopy level, while physiological and biochemical analyses were carried out in different foliar and non-foliar green organs (flag leaf blades and sheaths, peduncles, awns, glumes, and lemmas) at two specific stages; anthesis (Zadoks 65) and mid-grain filling (MGF; Zadoks 75). For these stages, the phenology of each variety was considered. Therefore, the samplings at anthesis for Kiko Nick and Don Ricardo were performed at 181 days after sowing (DAS) and for Haristide and Euroduro at 187 DAS, while at MGFEuroduro, Kiko Nick and Don Ricardo were sampled 195 DAS and, Haristide, 207 DAS.

### Agronomic Components and Grain Quality Traits

At maturity, plant height (from the soil surface to the ear, excluding the awns) was evaluated. By this time, 1 week before the harvest, we had determined the number of plants per m^2^ and ears per plant in two 0.5-m-length samples from the central rows of the plots. These samples were drying at 70°C for 24 h to determine the aboveground biomass. Then, the number of grains per ear, thousand grain weight (TGW), peduncle length (from the last internode to the base of the ear), and ear length (excluding the awns) were calculated in a subset of 10 main stems per plot. At the end of the experiment, the plots were harvested mechanically, and GY was determined for each one and adjusted to a 10% moisture level. Harvest index (HI) was calculated as grain weight/(grain weight + biomass). A pool of 250 g of grains per plot was collected at harvest for qualitative analyses. Protein content was determined using an Infratec 1226 Grain Analyzer (Foss Analytical, Denmark) on a dry basis. The moisture content of the grains was measured with the GrainMoistureTester PM-450 (Kett, USA). Specific weight (SW) was determined according to AACC Method 55–10. The percentage of vitreous grains (vitreousness) was determined on two lots of 100 seeds. Whole-grain flour samples were obtained after using an LM 3100 Mill (Perten Instruments AB, Sweden). Then, gluten strength was evaluated by the sodium dodecyl sulfate sedimentation (SDSS) test (Axford et al., 1978), and the yellow-color index (b^*^, CIE L^*^a^*^b^*^ color system) by using a portable reflectance colorimeter (CR-310, Konica-Minolta Sensing Inc., Tokyo). Wet gluten (WG) and gluten index (GI) were determined according to the International Association for Cereal Science and Technology (ICC) Methods 155 and 158, respectively.

### Canopy Vegetation Indices and Leaf Pigments

At the canopy level, we measured the normalized difference vegetation index (NDVI) by using a hand-held portable spectroradiometer (GreenSeeker, NTech Industries, USA) and RGB (red-blue-green) indices through the crop cycle as a proxy for biomass and healthy and green vegetation. RGB images were acquired by holding a 20.1-megapixel camera (Sony ILCE-QX1, Sony Corporation, Japan) attached to a Monopod VCTMP1 (Sony Corporation, Japan) at 1 m above the canopy in a zenithal plane and focusing near the center of the plot. The images were analyzed for the calculation of the vegetation indices GA (green area) and GGA (greener green area) with BreedPix software (Casadesús et al., 2007). GA and GGA are the percentage of pixels in the image (values from 0 to 1) in the hue range of 60–180° (from yellow to bluish green) and 80–180° (from yellowish-green to bluish-green), respectively (Vergara-Diaz et al., 2016). Compared to GA, GGA excludes yellowish-green tones and estimates more accurately photosynthetically active biomass (Vicente et al., 2019b). In addition, CSI (crop senescence index) was calculated according to Zaman-Allah et al. (2015) as:


$$\begin{array}{l} CSI=\left[\left(GA-GGA\right)/GA\right]\times 100 \end{array}$$

At the leaf level, the relative contents of chlorophylls (chl), flavonols, and anthocyanins, as well as the N balance index (NBI), were measured with the leaf-clip sensor (DUALEX, Force A, France) at Zadoks 65 and 75. This portable device measures the UV absorbance of the leaf epidermis by double excitation of chlorophyll fluorescence, which allows the calculation of reflectance (chlorophyll) and fluorescence (flavonols and anthocyanins) indices, as well as the ratio between chlorophylls and flavonols as a proxy for nitrogen/carbon balance (NBI). The measurements were carried out in the middle of the flag leaf blades of five plants per plot selected randomly, and then the values were averaged per plot, being the same plants that were collected for the biochemical analyses described below. All these phenotyping measurements were done around noon (12–14 h, UTC + 1) on sunny days.

### Relative Leaf Water Content and Fresh and Dry Weight

Leaf relative water content (LRWC) was determined according to Estévez-Geffriaud et al. (2020) at Zadoks 65 and 75 using the fresh (FW), turgid (TW), and dry (DW) weights of flag leaf blades in the following equation:


$$\begin{array}{l} LRWC\;\left(\%\right)=\left[\left(FW-DW\right)/\left(TW-DW\right)\right]\times 100 \end{array}$$

In parallel, five plants per plot were collected at Zadoks 65, 75, and 85 (soft dough) and used to calculate FW and DW of different organs (after drying in the oven at 70°C for 48 h): flag leaf blades and sheaths, peduncles, and whole ears.

### Determination of the Content of Carbohydrates

Foliar and non-foliar green organs were harvested with liquid N_2_ at Zadoks 65 and 75 at noon on sunny days and stored at −80°C. While blades, sheaths, and peduncles were grounded to fine powder with a mill (Mixer Mill MM300, Retsch GmbH, Germany), the awns, glumes, and lemmas were grounded manually using a mortar and pestle with liquid N_2_ to allow the proper separation of the organs. First, the soluble carbohydrates glucose, fructose, sucrose, and fructans were extracted from aliquots of the green organs using serial extractions with boiling ethanol as described by Stitt et al. (1989). A water extraction step was added to solubilize the fructans. Then, the supernatants were lyophilized and resuspended in bidistilled water, while the insoluble residue was used to determine starch after incubating with amyloglucosidase and α-amylase at 37°C overnight (Morcuende et al., 2004). The different carbohydrates were analyzed in 96-well plates using a Synergy 2 multi-mode microplate reader (BioTek, Germany) with a spectrophotometric assay coupled to NADP reduction as described by Morcuende et al. (2004).

### Quantification of Rubisco Large Subunit Content

Aliquots of finely powdered material from each green organ at Zadoks 65 and 75 were used for the extraction of proteins by mixing with 10 volumes of an extraction buffer that contains Tris/HCl 62.5 mM pH, 6.8; glycerol, 10% (v/v); sodium dodecyl sulfate (SDS), 2% (w/v); bromophenol blue, 0.0125% (w/v); β-mercaptoethanol, 0.05% (v/v). Then, the samples were heated at 95°C for 5 min and centrifuged at 13,000 g for 5 min. Aliquots of the supernatants were immediately subjected to SDS–polyacrylamide gel electrophoresis (SDS-PAGE) in 12.5% (w/v) polyacrylamide gels (0.75 mm of thickness), containing SDS, 0.1% (w/v), in a Mini-PROTEAN Tetra Cell system (Bio-Rad, USA). Additionally, PageRuler prestained a protein ladder (10–180 kDa, Thermo Fisher Scientific, USA), and bovine serum albumin (BSA) was used as molecular weight and concentration standards, respectively. The electrophoresis was performed at room temperature at a constant 200 V. Afterwards, the gels were stained in a solution, containing 5:4:1 (v/v/v) water–methanol–acetic acid mixture with 0.001% (w/v) Coomassie brilliant blue R-250 dye (Thermo Fisher Scientific) for 1 h, and subsequently rinsed in water to remove excess stain. The gels were scanned in a ChemiDoc MP imaging system (Bio-Rad) and the amount of Rubiscolarge subunit determined by densitometry with Image Lab software (Pérez et al., 2011; Vicente et al., 2016).

### Measurement of C-N Enzyme Activities

The enzyme activities of Rubisco (initial and total), phosphoenolpyruvate carboxylase (PEPCase), glutamine synthetase (GS), ferredoxin-dependent glutamate synthase (GOGAT), and NADH-dependent glutamate dehydrogenase (GDH) were determined in green organs at Zadoks 65 and 75. Enzymes were extracted from 20-mg aliquots of finely powdered material by adding 10 mg of polyvinylpolypyrrolidone (w/v) and 1 ml of an ice-cold extraction buffer containing Hepes/KOH, 50 mM, pH 7.5, MgCl_2_ 10 mM; (ethylenedinitrilo) tetraacetic acid (EDTA), 1 mM; ethylene-bis (oxyethylenenitrilo) tetraacetic acid (EGTA) 1 mM; benzamidine, 1 mM; ε-aminocapronic acid, 1 mM; BSA, 0.25% (w/v); leupeptin, 20 mM; 1,4-dithiothreitol, 0.5 mM; Triton X-100, 1% (v/v); glycerol, 20% (v/v); and phenylmethylsulfonyl fluoride, 1 mM. After centrifugation at 14,000 g and 4°C for 10 min, appropriate dilutions of the supernatants were rapidly used for the different enzyme assays as described in Sulpice et al. (2007) for Rubisco and in Gibon et al. (2004) for the other enzymes. The assays were carried out in 96-well microplates using ELx800 microplate readers (Bio-Tek, USA) at the Max Planck Institute of Molecular Plant Physiology (Germany). Rubisco activation state was calculated as the ratio between initial and total Rubisco activity.

### C and N Isotope Signature and Nutrient Composition in Green Organs and Grains

The flag leaf blades and sheaths, peduncles, and whole-ears samples used to calculate DW per organ at Zadoks 65 and 75 were finely grounded and used to assess the stable C (δ^13^C) and N (δ^15^N) isotope composition and the total C and N contents in the dry matter with an elemental analyzer Flash 1112 EA (Thermo Finnigan, Germany), coupled with an isotope ratio mass spectrometer Delta C IRMS (Thermo Finnigan) at the Scientific-Technical Services of the University of Barcelona (CCiTUB, Spain) as described in Medina et al. (2016). Moreover, grains at Zadoks 75, 85, and 92 (grain hard, not dented by thumbnail) were also collected and dried for 48 h at 70°C for the same analyses. The contents of other macro- and micro-nutrients were also analyzed in green organs collected at Zadoks 65 and 75 (K, Ca, P, Mg, Fe, Mn, Cu) and in grains at Zadoks 92 (K, P, S, Mg, Ca, Mn, Fe, Na, Zn, Cu, Mo). For each sample analyzed, around 500 mg of dried material was mixed with 8 ml of HNO_3_ 60% and 2 ml H_2_O_2_ 30% in a Teflon container. Then, the samples were subsequently digested at 200°C in a microwave digestion system (ETHOS UP, Milestone, Italy). Afterwards, the solutions were cooled to room temperature and diluted to 25 ml by adding deionized water. Nutrient concentration was determined at the Analysis and Instrumentation Service of IRNASA-CSIC (Spain) with an inductively coupled plasma-optical emission spectrometer (ICP-OES Varian 720-ES, Agilent Technologies, USA). The yield of each nutrient and protein in grains at harvest was calculated by multiplying their concentration by GY.

### Statistical Analysis

All the variables were subjected to two-way ANOVA using the general linear model to calculate the effects of N, genotypic variability, and their interaction by using IBM SPSS v23.0 (SPSS Inc., USA). To analyze the differences between the means of the specific groups, we used the Tukey's honest significant difference (HSD) test. Significance was accepted at *p* < 0.05. The heatmap tables were prepared in Microsoft Excel 2016 using the conditional formatting (Microsoft Corporation, USA), while the rest of the figures were prepared in R environment. The scatter and bar plots were generated using the package *ggplot2*. Stepwise regressions were performed using the *stepAIC()* function, while the proportion of variance explained by each predictor was calculated with the package *relaimpo*. The packages *factoextra* and *FactoMineR* were used to extract and visualize the multivariate data analyses (PCA, principal component analysis). Pearson correlation matrices were built to analyze the relationships between trait pairs using the function *cor()*, and visualized using the package *corrplot*. The abbreviations for each trait used in the figures and tables are summarized in Supplementary Table 1.

## Results

### Effect of N and Genotypic Variability on Durum Wheat Agronomic Components, Grain Quality, and Physiology

**Figure 2:**
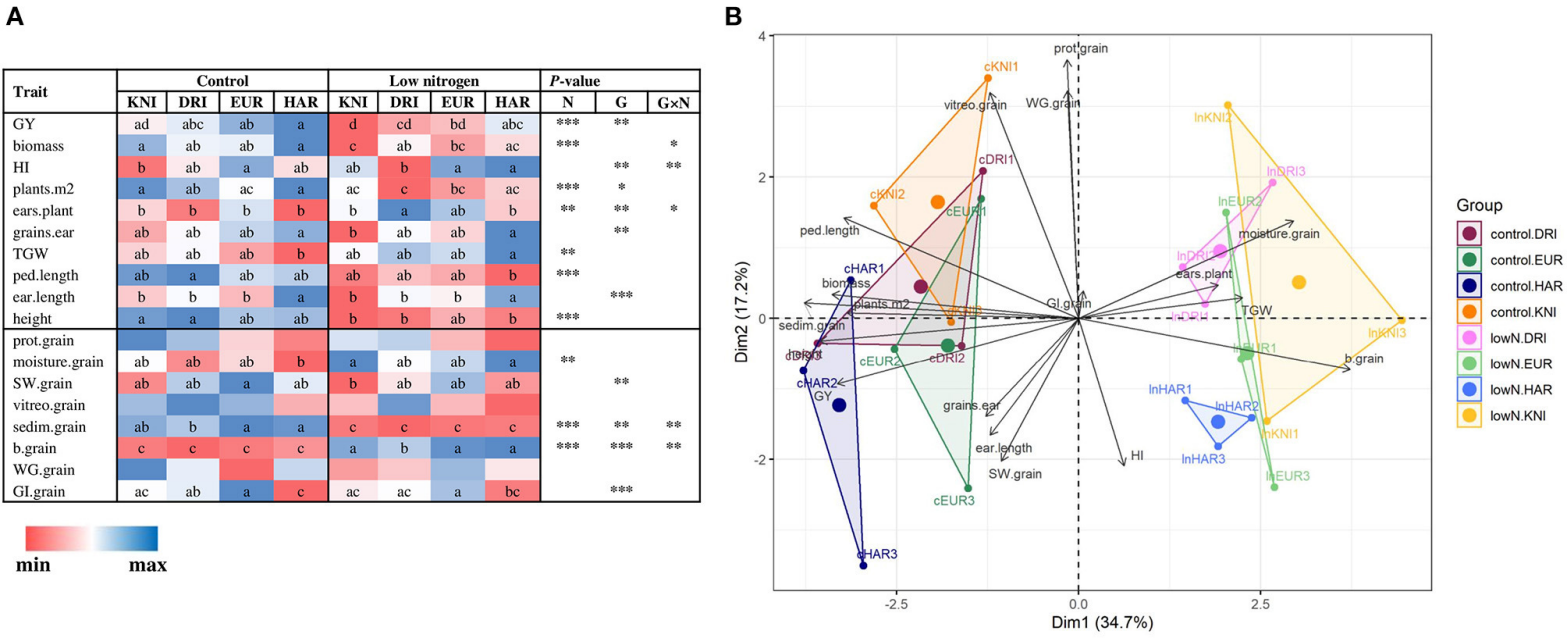
Effects of N supply and genotypic variability on agronomic components and grain quality traits **(A)** and principal component analysis **(B)** in four varieties of field-grown durum wheat (Kiko Nick, Don Ricardo, Euroduro, and Haristide) at two N levels (control vs. low N). In **(A)**, the different letters differ statistically (*, *p* < 0.05; **, *p* < 0.01; ***, *p* < 0.001). The abbreviations are described in Supplementary Table 1.

Low N reduced GY (22%), biomass (25%), and plants per unit area (29%), as well as peduncle length and plant height, compared to control N, while TGW slightly increased (Figure 2A; Supplementary Table 2). For GQ, low N significantly reduced sedimentation index and increased moisture content and yellowness index (Figure 2A; Supplementary Table 2). The different N supplies were undoubtedly separated in the PCA by X-axis, representing a 34.7% of the variability. The four varieties clearly showed differences in agronomic and GQ traits, with a similar trend at each N supply as evidenced by the low number of significant G × N interactions and their distribution in the PCA (Figure 2). The variety Haristide had the highest GY regardless of N supply, followed by Euroduro, Don Ricardo, and Kiko Nick (Figure 2A). Most changes in agronomic components followed the trend observed in GY, being Kiko Nick the one with lower values and Haristide with higher ones. These differences were slightly more pronounced under low N than control N. In the PCA, Y-axis explained 17.2% of the variance in the data, which was partially related with differences among varieties (Figure 2B). The most relevant GQ traits in this axis were grain protein content, WG, GI, and SW.

**Figure 3:**
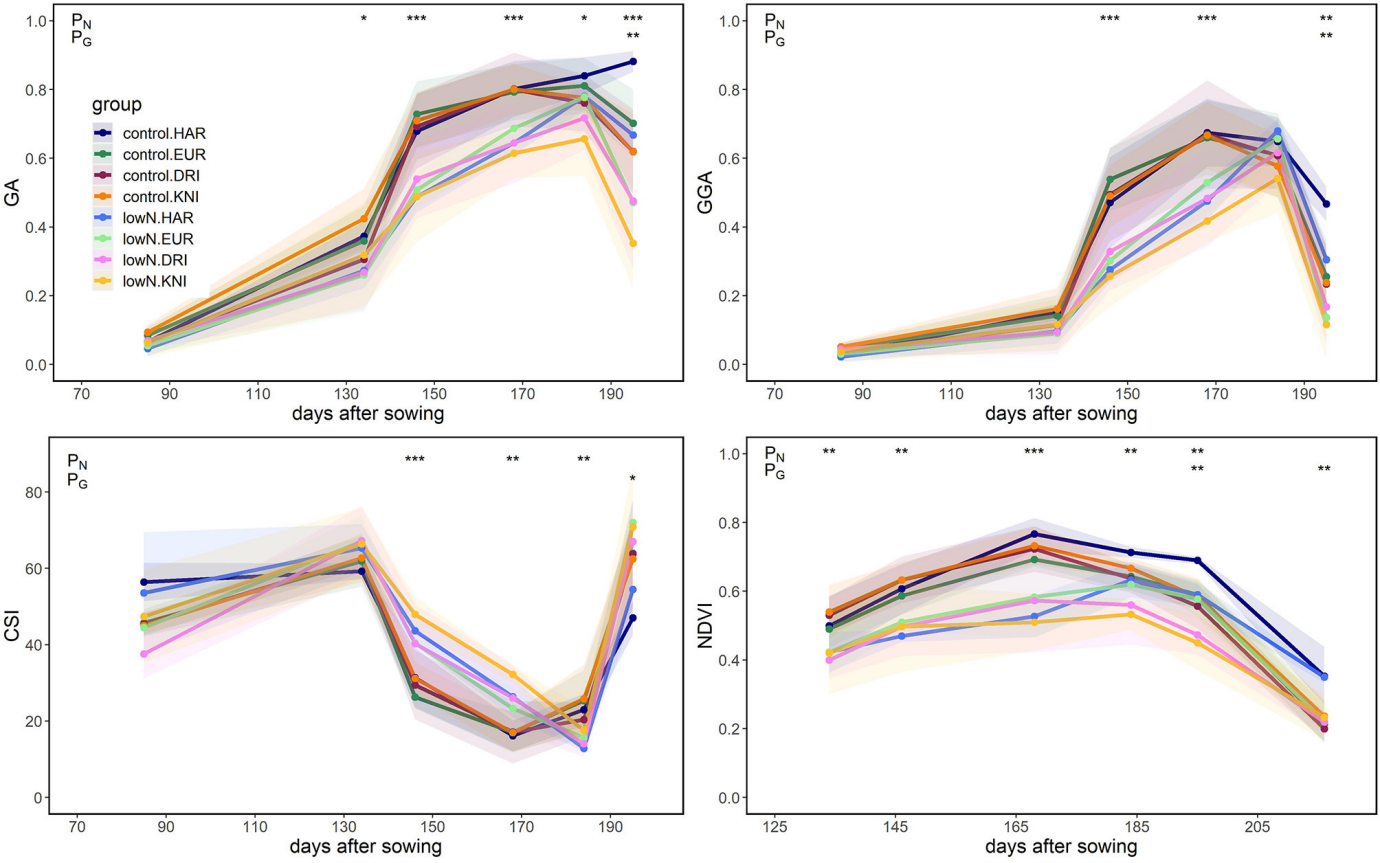
Green area (GA), greener green area (GGA), crop senescence index (CSI), and normalized difference vegetation index (NDVI) in four varieties of field-grown durum wheat (Kiko Nick, Don Ricardo, Euroduro, and Haristide) at two N levels (control vs. low N) measured at the canopy level. Asterisks indicate a significant difference between varieties (G) and N levels (N) according to the two-way ANOVA(*, *p* < 0.05; **, *p* < 0.01; ***, *p* < 0.001). The interaction G × N did not reach significance for these parameters.

Ground phenotyping was performed to monitor plant growth, pigment content, and senescence at canopy and leaf levels. Low N supply significantly decreased GA, GGA, and NDVI, and increased CSI from early stages to maturity (Figure 3). Minor changes, albeit significant, were observed among varieties, mainly at late-growth stages where Haristide showed a better performance regardless of the N supply (Figure 3; Supplementary Table 2). Leaf flavonols content was increased under low N compared to control, being significant at anthesis (Supplementary Figure 1). The flavonols content was higher in Kiko Nick, followed by Don Ricardo, Euroduro, and Haristide at both growth stages independently of the N supply, while NBI tended to be higher in Haristide and Euroduro.

Low N decreased LRWC significantly at anthesis and MGF, while Haristide and Euroduro had higher LRWC at anthesis than Kiko Nick and Don Ricardo (Supplementary Table 2). Regarding the effect of N on organ weights, low N decreased FW at anthesis and MGF and DW at Zadoks 85 in blades, and DW at Zadoks 85 in sheaths and peduncles, but not in ears (Supplementary Table 2). Genotypic variability affected organ weights, mainly blades, sheaths, and peduncles at anthesis and MGF, while ear DW was the lowest in Kiko Nick at late-grain filling regardless of N supply.

### Correlations Between Agronomic Components and Grain Quality and Physiological Traits

**Figure 4:**
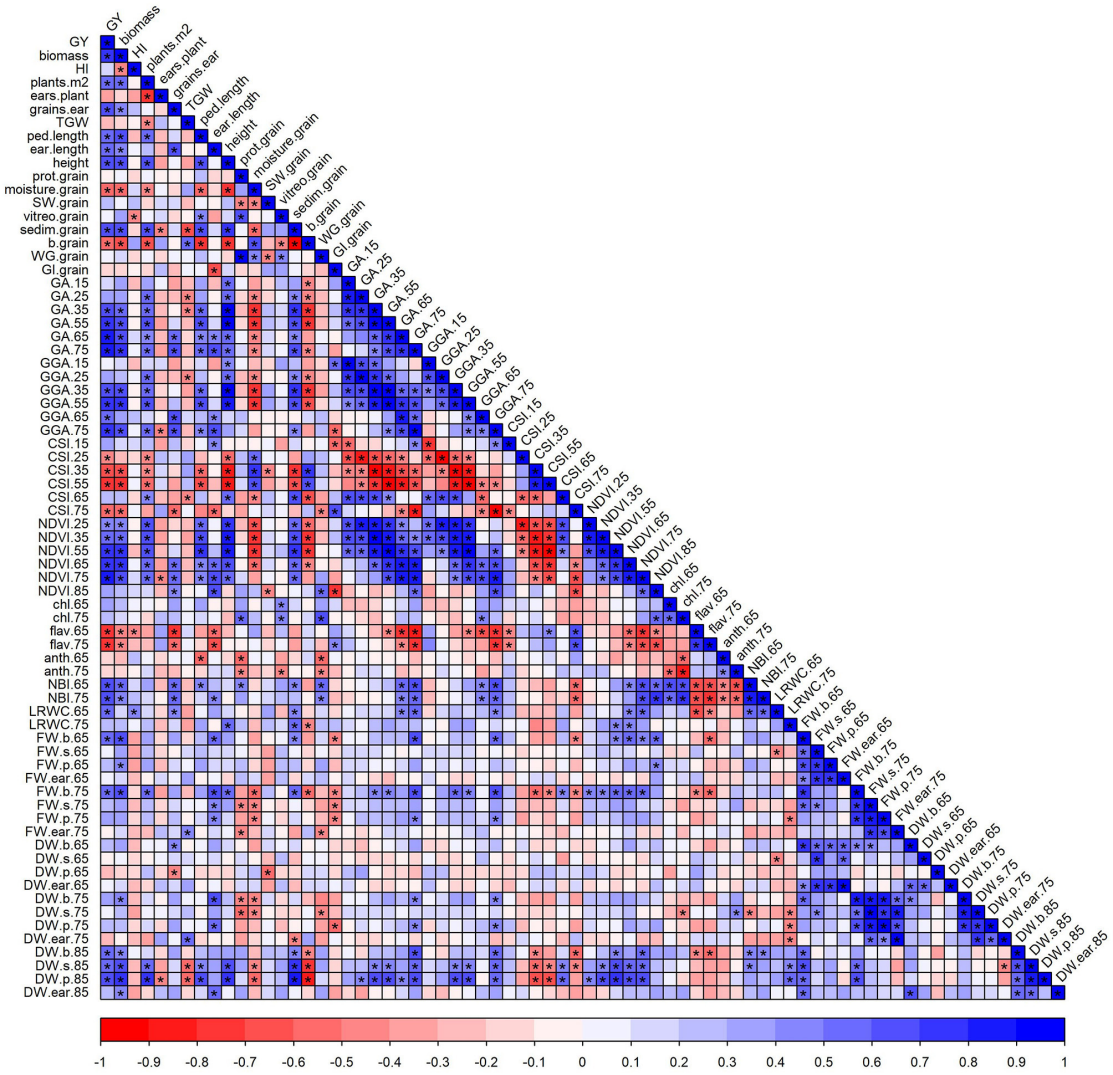
The correlation matrix of agronomic components and grain quality and physiological traits. Each point of the matrix is a Pearson correlation coefficient between two traits (blue, positive correlation; red, negative correlation). Asterisks indicate a significant correlation (*p* < 0.05). The abbreviations are described in Supplementary Table 1.

GY was positively correlated with several agronomic components, such as biomass, plants per unit area, grains per ear, peduncle and ear lengths, and plant height (Figure 4). GY also correlated with the GQ traits sedimentation index, positively, and, with yellowness index, negatively. In addition, significant positive correlations were also observed between GY and traits, such as LRWC and blade FW at both anthesis and MGF, and blade, sheath, and peduncle DW at late-grain filling. Among the significant correlations between agronomic components and GQ traits, the most interesting were the correlations between GY and grain moisture content, and the sedimentation and yellowness indices. Grain protein correlated positively with vitreousness, WG, leaf chl, and N contents, and negatively with SW, leaf anthocyanins, and some organ weights (Figure 4). Biomass was highly correlated with GY and, therefore, the NDVI and RGB canopy indices with higher correlation coefficients as growth progressed (Figure 4). The leaf spectral indices were good proxies for GY, yield-related traits, and biomass, particularly flavonols and NBI. These indices were also correlated with several GQ traits, such as grain protein content, vitreousness, sedimentation index, WG, and GI.

### Effect of N and Genotypic Variability on Grain Nutrient Compositions in Field-Grown Durum Wheat

**Figure 5:**
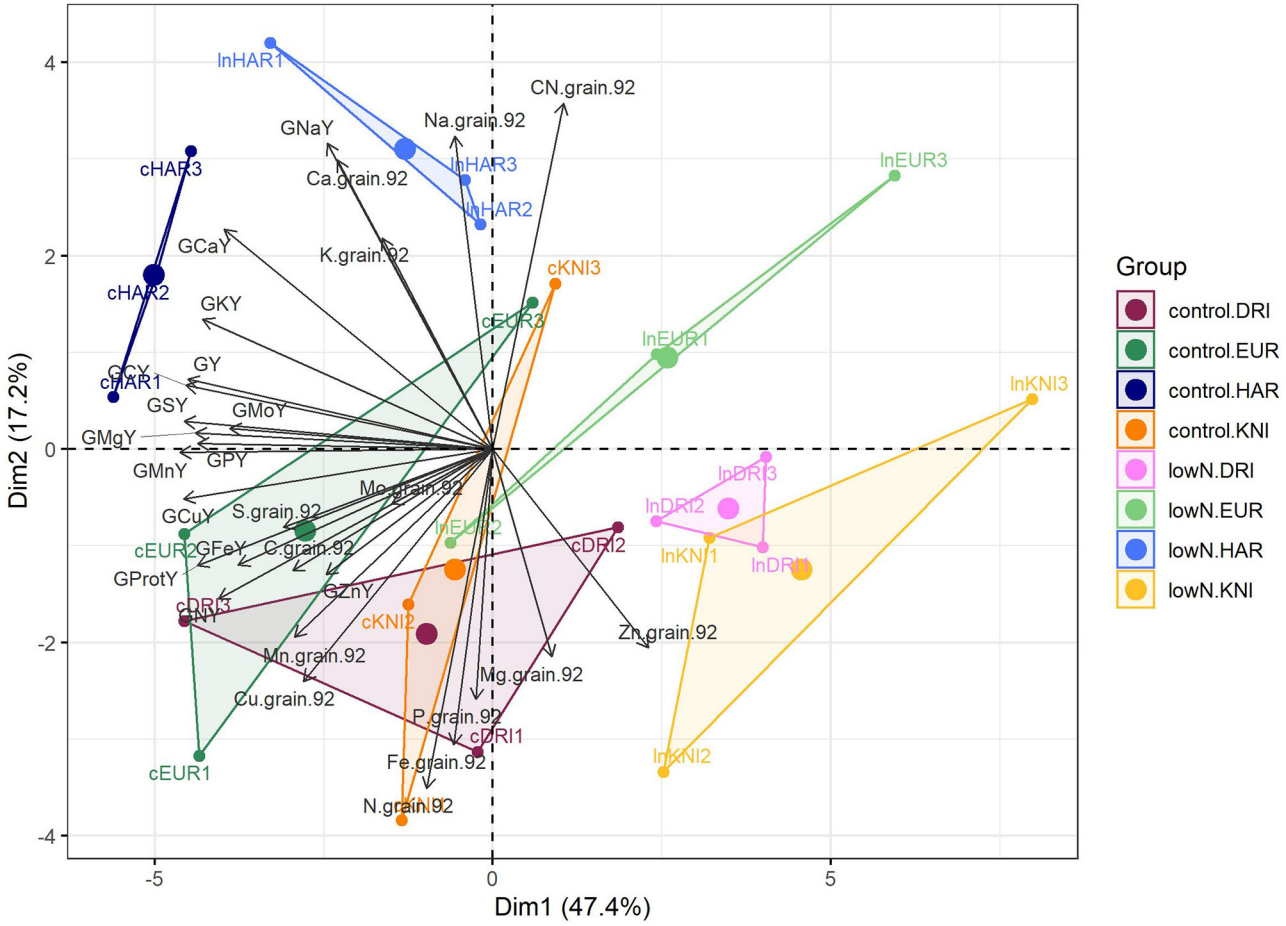
Principal component analysis of grain nutrient compositions and yields at harvest in four varieties of field-grown durum wheat (Kiko Nick, Don Ricardo, Euroduro, and Haristide) at two N levels (control vs. low N). The abbreviations are described in Supplementary Table 1.

We built a PCA with grain and protein yields and 13 grain minerals expressed as concentrations and yields measured at harvest to further characterize GQ under G × N interaction (Figure 5). X-axis and Y-axis explained 47.4 and 17.2% of the variance in the data, respectively, and they were associated with changes due to both N treatment and genotypic variability. N effect was clear, while the differences between varieties were similar under control or low N supply. Control N was certainly associated with grain protein and N yields, and nutrients, such as C, S, Fe, P, Cu, and Mn. The high-yielding Haristide had the highest concentrations of nutrients, such as Ca, K, and Na, and the lowest of Zn (Figure 5), being statistically significant Ca and Zn by Tukey's HSD test (Supplementary Table 2). Considering the nutrient amounts by yields, low N undoubtedly decreased the uptake of most of them, while Haristide was the variety that uptakes highest levels of most nutrients and Kiko Nick the least regardless of N supply.

### Primary Metabolism and Nutrient Composition in Green Organs of Durum Wheat During Grain Filling Under Contrasting N Supply

**Figure 6:**
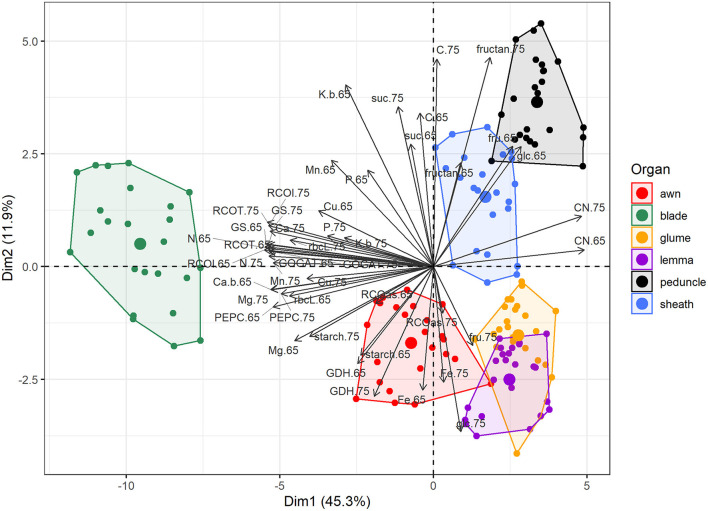
Principal component analysis of metabolites, enzyme activities, and nutrient composition in different organs (blade, sheath, peduncle, awn, glume, and lemma) in field-grown durum wheat. The abbreviations are described in Supplementary Table 1.

We examined the metabolism of photosynthetic green organs by determining carbohydrates and Rubisco protein contents, C-N metabolism enzyme activities, and nutrient and isotope composition. The blade was significantly separated from the other organs in the PCA due to its higher values for most of these traits, e.g., Rubisco protein, Rubisco, PEPCase, GS, and GOGAT activities, and nutrients, such as N, Ca, Mg, Mn, and Cu (Figure 6). Initial Rubisco activity was 42–56, 23–39, 14–18, 11–18, and 11–14% in awns, sheaths, peduncles, glumes, and lemmas, respectively, compared to blades depending on N supply and the growth stage (calculated from Supplementary Table 2). Similar patterns were observed for total Rubisco activity, which correlated with protein level (*r* = 0.80), as well as for the other enzymes, except GDH whose values were not so low in the non-foliar organs. Considering X-axis (45.3% of the variability), the closer organ to the blade was the awn. Ear bracts, glumes, and lemmas almost overlapped and were close to awns, being separated from sheaths and peduncles mainly by Y-axis (11.9% of the variability). Ear organs had high levels of GDH activity, Rubisco activation state, and fructose, glucose, and Fe levels at MGF. Sheaths and peduncles were characterized by the highest fructans levels and free carbohydrates (glucose and fructose) at anthesis.

The N effect was relatively similar between organs, but when the position of the centroids was examined per organ and N supply, we observed slightly stronger effects on blades, while, in other organs, such as the ear bracts, it was smaller or almost negligible (Supplementary Figure 2). Low N increased the levels of fructose, fructans, and starch in the peduncle and sucrose in blades at anthesis compared to control N (Supplementary Table 2). Interestingly, low N decreased sucrose levels in blades, sheaths, and, more significantly, in ear organs at MGF, together with starch. Low N increased Rubisco protein in peduncles and lemmas at anthesis but reduced it in sheaths and peduncles at MGF and in blades at both stages (Supplementary Table 2). Low N tended to decrease both initial and total Rubisco activities in most organs, except for a small increase in blades and a stronger increase in peduncles at anthesis. Low N also decreased activities of PEPCase (e.g., in glumes, sheaths, and peduncles), GS, and GOGAT, while GDH was less affected, with an interesting strong increase in blades in low N compared to control N. Although the N effect on grain nutrient composition at harvest was clear (Figure 5), its effects were not so evident in the organ-specific nutrient concentrations (Supplementary Table 2). Among the most relevant data, lower N supply increased Fe content, especially in glumes, and reduced C content at late stages and, non-significantly, N.

The genotypic variability greatly influenced primary metabolism and nutrient composition in green organs (Supplementary Figure 3, Supplementary Table 2). Fructose levels were higher in most green organs of high (Haristide and Euroduro) vs. low-yielding (Don Ricardo and Kiko Nick) varieties (Supplementary Table 2). Sucrose content was strongly reduced in Haristide in all organs at anthesis, but, at MGF, it was higher in blades, awns, glumes, and lemmas compared to the other varieties, regardless of N supply. The pattern of changes in starch was very similar to sucrose, while overall fructans decreased in Haristide, except for an increase in peduncles at MGF. Rubisco protein content and activities were organ specific and highly variable between varieties. Due to the amount of traits, treatments, and significant results obtained for the other enzyme activities and nutrients, we focused on their correlations with agronomic components and GQ traits detailed in the following sections. An overview of the most relevant results for each variety and organ is shown in Supplementary Figure 3. In summary, the metabolism and nutritional composition of the high-yielding variety Haristide were markedly different from the others at the whole plant.

### Changes in Primary Metabolism and Nutrient Composition in Green Organs Between Anthesis and Mid-Grain Filling

**Figure 7:**
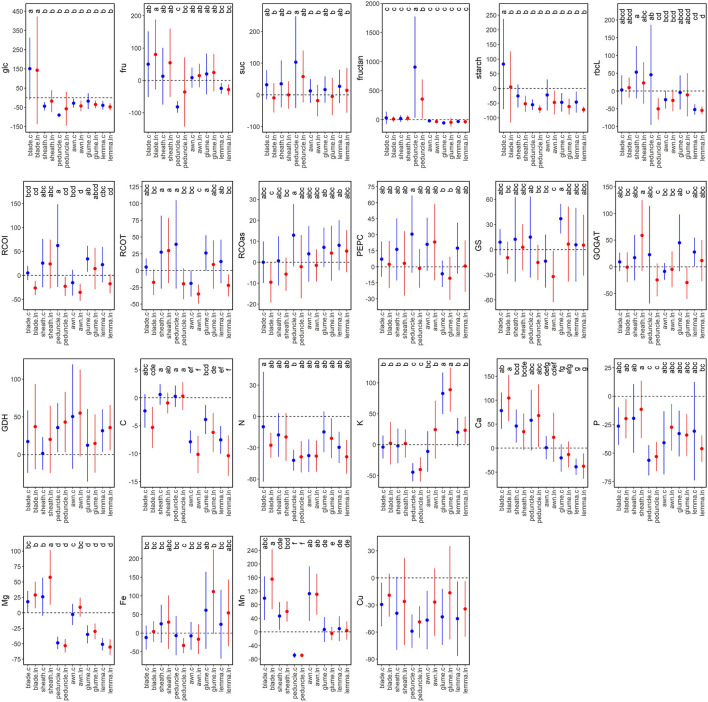
Percentage (%) of variation between anthesis (Zadoks 65, blue) and mid-grain filling (Zadoks 75, red) for the metabolite contents, Rubisco large subunit protein, enzyme activities, and nutrient contents. Each dot is the average of four field-grown durum wheat varieties per organ (blade, sheath, peduncle, awn, glume, and lemma) and N supply (c, control; ln, low N). The different letters differ statistically (*p* < 0.05). The abbreviations are described in Supplementary Table 1.

Metabolic changes between anthesis and MGF were studied by organ (grouping all varieties as their differences were similar for each N regime) to understand their metabolic evolution during grain filling (Figure 7). Glucose content at both N supplies and starch at control N increased in blades and decreased in other organs at MGF compared to anthesis. Fructose content increased in blades, particularly under low N, while decreased in peduncles compared to other organs. Sucrose content was slightly higher in all organs under control N at MGFcompared to anthesis, while it was similar under low N, except for the high increase in peduncles at both N levels. A marked increase in fructans was observed in peduncles, higher under control N. The amount and activity of Rubisco in peduncles increased at MGF under control N but decreased under low N, while they increased in sheaths. In ear organs, i.e., glumes and lemmas, Rubisco protein content tended to decrease, but the initial activity increased, partly due to a better activation state (Figure 7). By contrast, Rubisco activity was significantly decreased in blades at MGF under low N. PEPCase activity tended to increase at MGF, being negatively affected by low N, higher in peduncles under control N and lower in glumes. GS activity increased remarkably in glumes under control N and decreased in awns under low N. GOGAT activity was stable in blades, increased in sheaths under low N and glumes under control N, or decreased in peduncles and glumes under low N. In general, GDH activity was higher at MGF. The nutrients exhibited significant differences between the organs but limited N effects (Figure 7). MGF led to a decrease of C in blades and more strongly in ear organs, N, P (more in peduncles), Cu, K, and Mn in peduncles; Ca in ear bracts; and Mg in peduncles and ear bracts, and an increase of K in glumes; Ca in blades, peduncles, and sheaths; Mg in blades and sheaths; Fe in ear bracts; and Mn in blades, sheaths, and awns.

### Correlations Between Agronomic Components and Grain Quality Traits With the Metabolic Status of Green Organs

**Figure 8:**
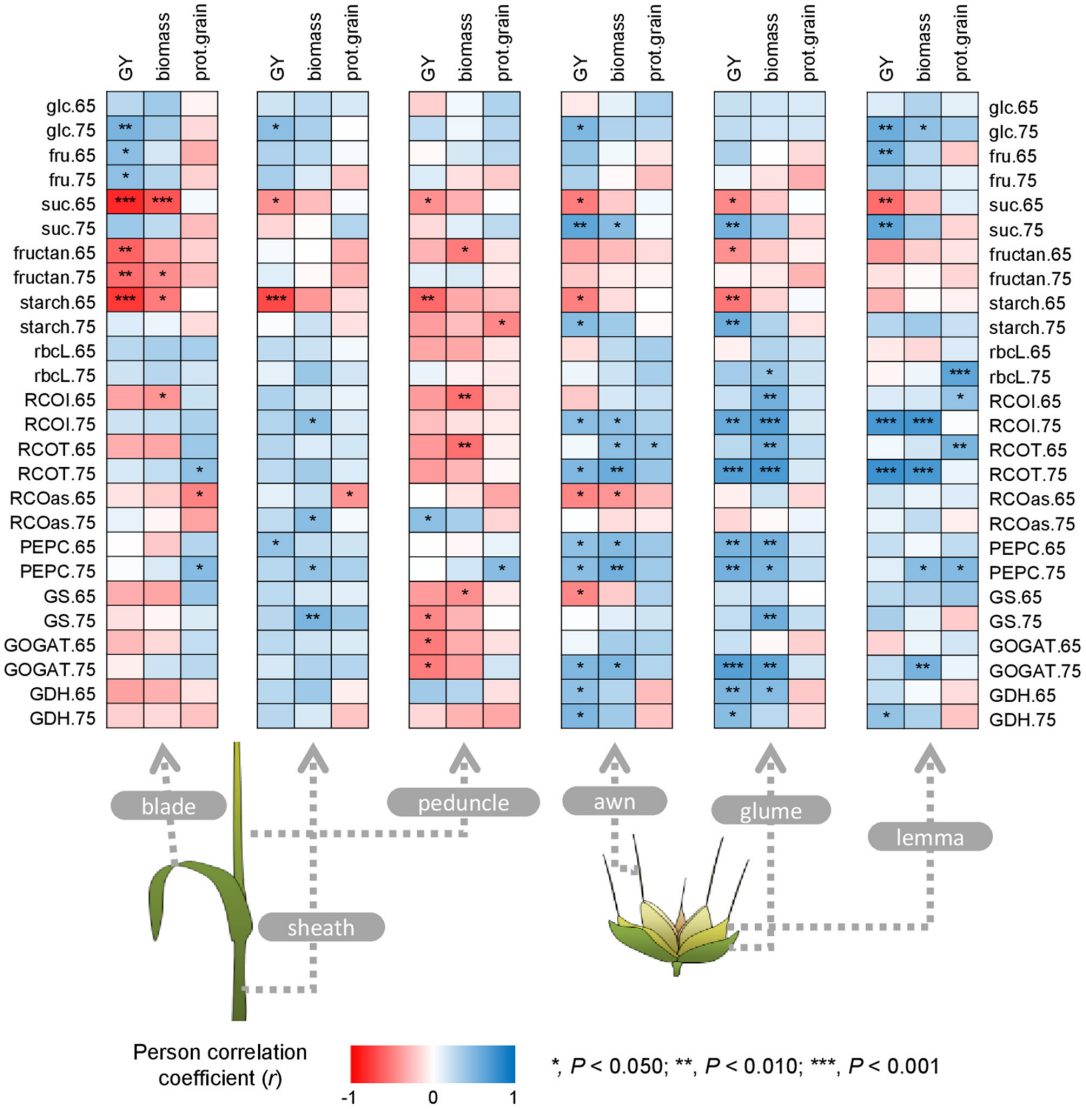
Correlations between metabolic traits in the different green organs at anthesis (Zadoks 65) and mid-grain filling (Zadoks 75) and agronomic components and grain quality traits at harvest. Each point is a Pearson correlation coefficient between two traits (blue, positive correlation; red, negative correlation). Asterisks indicate a significant correlation according to the legend. The abbreviations are described in Supplementary Table 1.

In blades, GY correlated positively with free carbohydrates and negatively with sucrose, fructans, and starch, also observed for biomass (Figure 8). Grain protein content was positively associated with total Rubisco and PEPCase activities at MGF, and negatively with Rubisco activation state at anthesis. In sheaths, GY correlated positively with PEPCase activity at anthesis and glucose content at MGF, and negatively with sucrose and starch contents at anthesis. Biomass correlated with Rubisco, PEPCase, and GS activities at MGF, while grain protein content correlated negatively with Rubisco activation state at anthesis. In peduncles, sucrose, and starch contents at anthesis, GS activity at MGF, and GOGAT activity at both stages correlated negatively with GY. Negative correlations were also observed for biomass and the peduncle biochemical related traits, i.e., fructans, initial and total Rubisco and GS activities at anthesis. Grain protein content only correlated with starch and PEPCase activity at MGF. In awns, glumes, and lemmas, GY generally correlated negatively with sucrose and starch at anthesis and positively with sucrose and starch, initial and total Rubisco and GOGAT activities at MGF, and PEPCase activity at both growth stages. GY also correlated positively with free carbohydrates in awns and lemmas, and negatively with Rubisco activation state and GS activity in awns at anthesis. Correlations between metabolic traits and biomass in ears were similar to those found for GY, with a remarkable correlation between biomass and Rubisco, PEPCase and GOGAT activities. Grain protein content predominantly correlated with lemma metabolic traits, such as Rubisco protein and Rubisco and PEPCase activities.

**Figure 9:**
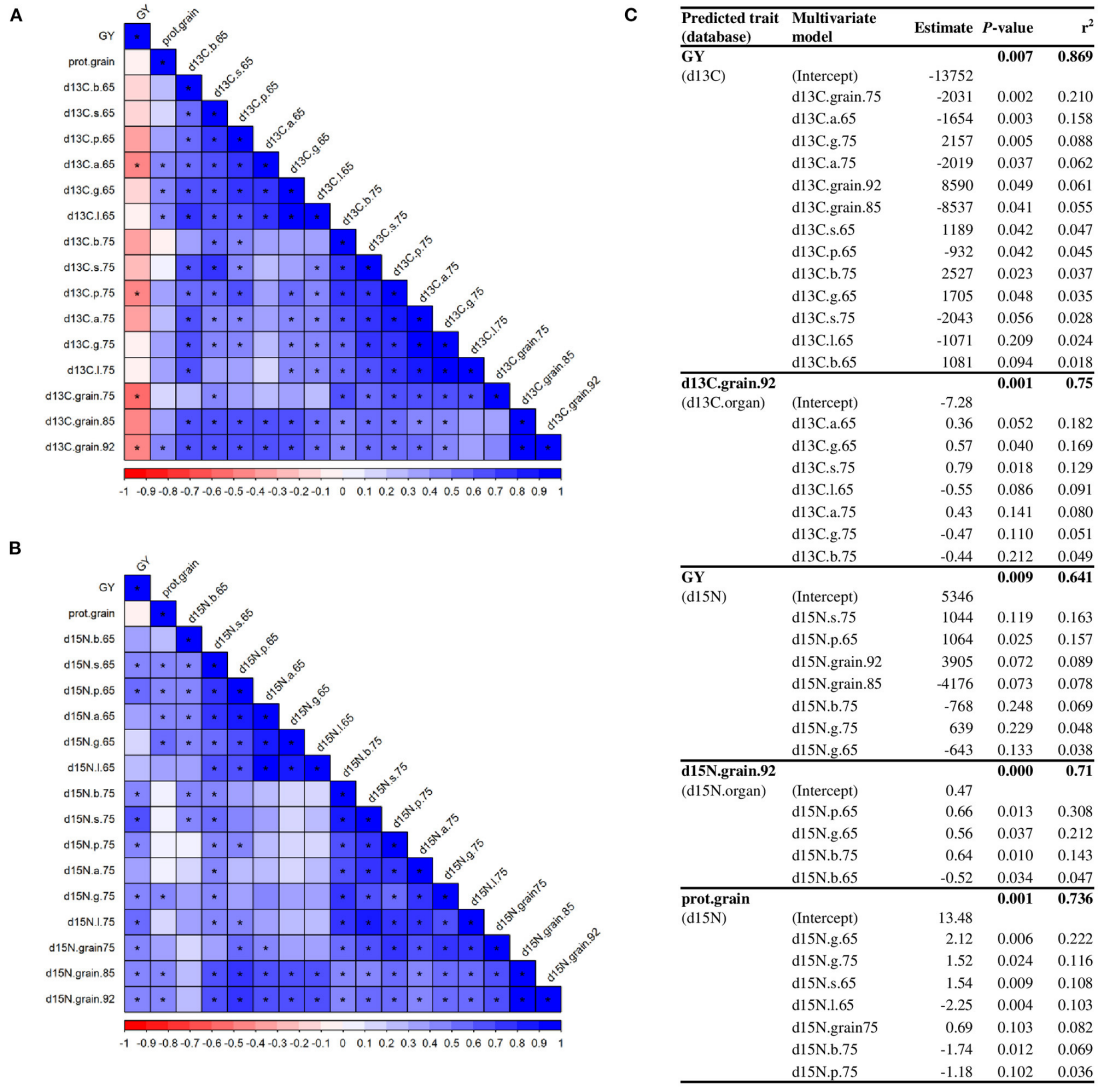
The correlation matrix of C **(A)** and N **(B)** isotope composition per organ with grain yield and grain protein content. Asterisks indicate a significant correlation (*p* < 0.05). Multivariate regression models **(C)** explaining grain yield and C and N isotope composition in grain at harvest across varieties under different N supplies. The abbreviations are described in Supplementary Table 1.

We used the isotope signatures of green organs to predict their contribution to grain filling and GY. A high number of significant correlations were observed between the δ^13^C of organs and grains (Figure 9A). GY correlated negatively with δ^13^C of awns at anthesis, peduncles at MGFand grains at MGF and harvest, while grain protein content correlated with δ^13^C of awns, glumes, and lemmas at anthesis and grains at harvest. Similar to δ^13^C, there were a high number of correlations between δ^15^N of the organs with the one in grains, GY and grain protein content (Figure 9B). Moreover, we used regression models with the organ-specific isotope compositions to predict GY and grain δ^13^C, δ^15^N, and protein content (Figure 9C). According to the proportion of variance explained by each predictor (*r*^2^), GY was mainly predicted by δ^13^C of grains, awns and glumes, and grain δ^13^C at harvest by δ^13^C of ear organs and sheaths. Using the δ^15^N values, GY was predicted by δ^15^N of sheaths, peduncles, grains, blades, and glumes, δ^15^N of grains at harvest by δ^15^N of peduncles, glumes, and blades, and grain protein content by δ^15^N of many organs, predominantly the glumes.

## Discussion

We performed a holistic study integrating phenotyping measurements of canopy and flag leaves and biochemical analyses of six foliar and non-foliar photosynthetic organs to identify the traits at the whole plant level, which are related to plant growth, GY, and GQ in field-grown durum wheat. A total of 426 traits were studied in four varieties grown under contrasting N fertilization conditions. The pattern of changes among varieties was similar under both N conditions, as shown by the low G × N interaction. Therefore, we focused our attention mainly on the effects of N and genotypic variability separately.

### N Fertilization Has a Significant Effect on Grain Yield and Quality, While Agronomic Differences Between Varieties Were Not Affected by N Availability

An efficient use of N fertilization that meets sustainability is necessary since it is the nutrient that most affects crop production and quality, but it is costly, and its excessive use can cause soil and water pollution (Vicente et al., 2019b; Wang et al., 2021). Then, it is important to identify those physiological and metabolic parameters affected by N, with an impact on GY and GQ. In our study, lower N supply decreased plant biomass by reducing plant height, peduncle length, and tillering, with a direct impact on GY (Figure 2). However, under N-limiting conditions, there were less plants per unit area, but they used efficiently their nutrients on producing more ears per plant with larger grains (i.e., higher TGW), as it has been previously shown in barley (Vicente et al., 2019b) and wheat (Liu et al., 2021). Grain protein content is frequently affected by N fertilization (Wang et al., 2021), but our contrasting N levels were not enough to alter it (Figure 2). Nevertheless, low N modified moisture content, and sedimentation and yellowness indices, which indicated an impoverishment of grain processing and end-product qualities (Zörb et al., 2018).

The variety with highest GY, Haristide, was characterized by shorter peduncles and longer and heavier ears capable of lodging more grains, parameters associated with higher sink strength (Figure 2; Supplementary Table 2). Genotypic variability did not alter grain protein content but affected other traits related to grain processing and end-product qualities. The most productive varieties Haristide and Euroduro had higher milling potential (SW), baking quality of wheat flour (sedimentation and yellowness indices) and, only for Haristide, lower gluten strength (GI). In Djouadi et al. (2021), durum wheat yield also correlated with several grain quality traits, but, frequently, a negative correlation is found with grain protein content.

### Phenotyping Approaches to Assess the Effect of N and Genotypic Variability on Grain Yield and Quality

Phenotyping approaches are suitable for characterizing plant performance and identifying key attributes for plant growth and production (Kefauver et al., 2017; Vicente et al., 2019b; Prey and Schmidhalter, 2020). We performed ground-based phenotyping to quantify canopy greenness and thus relate it to plant biomass and health (Casadesús et al., 2007; Vergara-Diaz et al., 2016). RGB and spectral indices were good predictors of the N fertilization on green biomass in durum wheat from early stages to maturity (Figure 3). These indices tend to saturate at intermediate growth periods, so their use is more valuable at early or late stages, as it happened in our study. Haristide showed a stay-green phenotype compared to the other varieties regardless of the N supply, which implies longer standing photosynthetically active biomass (Figure 3). Spectral indices measured at the flag leaf level suggested that Haristide had improved performance due to better N status (NBI index) and lower flavonoid content (Supplementary Figure 1), while higher LRWC at anthesis and grain δ^13^C indicated a better water status and water use efficiency, respectively (Rebetzke et al., 2002; Araus et al., 2021a).

High-throughput phenotyping has been used to predict yield-related traits in wheat, e.g., using unmanned aerial systems, multispectral cameras, and spectroradiometers (Prey and Schmidhalter, 2020; Garriga et al., 2021; Vatter et al., 2021), but their high cost and user training are drawbacks for their expansion. The vegetative indices used here quantified the greenness by counting pixels in the green-color range or by the spectrum reflected by the vegetation, so their high correlation with biomass and hence GY was not surprising (Figure 4). The correlations were higher as growth progressed, indicating that late stages are better for prediction, although this is not an advantage for the use of high-throughput phenotyping in early detection or breeding programs. The best leaf spectral indices for prediction were those estimating flavonoids and N content, which highlighted the relevance of antioxidant capacity and N status for productivity. Thus, the lower flavonoid content of Haristide compared with the other varieties may indicate a reduced need to produce antioxidants to counter the detrimental effects of reactive oxygen species that often occur during stress conditions or senescence (Agati et al., 2020). Canopy indices did not stand out for its prediction of GQ parameters, except for those traits that already correlated with GY (Figure 4). However, leaf spectral indices had potential to predict some key GQ traits. In short, our study highlighted the use of low-cost and affordable phenotyping devices (RBG imaging and leaf spectral sensors) to rapidly estimate the effects of N fertilization, to select high-yielding varieties, and to predict GY and GQ.

### Nitrogen Fertilization Affects the Uptake and/or Allocation of Micro- and Macronutrients to the Grain, While Ca and Zn Could Play an Important Role in Yield

The concentration of mineral elements in the grain is relevant for GQ and human diet, being determined by the genotype-by-environment interaction (Sanchez-Garcia et al., 2015; Guzmán et al., 2016). The nutrient concentrations varied between N supplies and varieties, with few significant G × N interactions (Figure 5). Higher N supply quantitatively increased grain N and thus protein yields, and the uptake and/or allocation of nutrients, such as C, S, Fe, P, Cu, and Mn to the grain. This may be associated with a promotion of root growth under higher N supply that favors the nutrients uptake, as suggested by Mariem et al. (2020). Nevertheless, an assessment of its cost benefit and associated environmental pollution is crucial when selecting the best application rate and timing (Kefauver et al., 2017; Vicente et al., 2019b).

The high-yielding Haristide had higher yields of most nutrients than the other varieties, indicating higher uptake of nutrients from the soil, regardless of nutrient concentration (Figure 5). Anyway, the concentration of nutrients in the grain is an important factor affecting the quality parameters by which wheat flours are graded. Haristide was mainly distinguished from the other varieties by a significantly higher Ca and lower Zn concentration, being Ca mainly accumulated in blades (Supplementary Table 2). Brennan et al. (2007) reported that Ca application had a direct impact on wheat yields, which can suggest that its better uptake in Haristide could be a key factor to increase production. Indeed, Ca may modulate the absorption and translocation of several elements and maintain the integrity of selective ion transport proteins (Cobalchin et al., 2021). Lower Zn content in Haristide, irrespective of N supply, could suggest a poor root uptake or remobilization from shoots to grains (Liu et al., 2019), which is relevant for human diet to avoid symptoms, such as loss of appetite, growth retardation, rough and peeling skin, and immune system dysfunction (Wang et al., 2020). Uauy et al. (2006) showed that delayed senescence may decrease N, Fe, and Zn content in the grain. Haristide showed a stay-green phenotype, which might explain the lower Zn content found in this variety. In conclusion, N fertilization is crucial to stimulate nutrient uptake, while higher GY was associated with better Ca status but lower Zn.

### Canopy Photosynthesis, N Assimilation, and C-N Allocation to the Grain Are the Result of a Common Effort of the Green Organs of the Plant

After characterizing wheat agronomy and canopy, we focused on the metabolism of green photosynthetic organs and their impact on GY and GQ. We hypothesized that non-foliar green organs have special physiological and metabolic features that make them suitable as source organs during grain filling, at least to complement the contribution of the flag leaf. This role has been predicted through other approaches under optimal and, more significantly, under stress conditions (Sanchez-Bragado et al., 2014a,b, 2016, 2020b; Vicente et al., 2018b), although the precise metabolic pathways operating in each part are poorly understood. Multivariate analysis of metabolic and mineral traits indicated that the metabolism in the blades was, undoubtedly, the most active (Figure 6). It was followed by the awns, albeit by a wide margin. The different ear bracts, which were very similar to each other, had a similar behavior to the awns. The peduncles and the sheaths were separated from the rest of the organs, suggesting they might have similar functions. Based on the PCA-centroids distribution of Supplementary Figure 2, it seems that N effect was more relevant on blades and less on bracts. Nevertheless, the differences were not very large, while Sanchez-Bragado et al. (2014a) found that the whole ear performance and contribution to grain filling improved under high N fertilization.

We measured different metabolism traits, such as Rubisco protein and activity, and the amount of photoassimilates, as an alternative to previous approaches to characterize photosynthetic capacity of non-foliar organs to GY, which were frequently intrusive or causing compensatory effects (Sanchez-Bragado et al., 2016; Rivera-Amado et al., 2020). The protein content and activities of Rubisco, directly involved in the fixation of atmospheric CO_2_, were significantly higher in blades, but not negligible in other organs, such as the awns, demonstrating active photosynthetic capacities at late stages, including a high degree of activation state in ear organs (Supplementary Table 2). Higher PEPCase activity was shown in blades and awns, which was associated with their higher photosynthetic capacity and the need to process the C fixed, but the activities in the other organs were remarkable (Supplementary Table 2). This enzyme is involved in the balance of C and N metabolism by regulating the synthesis of C skeletons for the synthesis and nitrogenous compounds and its possible role in the re-assimilation of CO_2_, such as grain respiration (Jia et al., 2015; Shi et al., 2015; Sanchez-Bragado et al., 2020b). Sucrose, which is the main compound used to transport C in cereals (Vicente et al., 2016; Al-Sheikh Ahmed et al., 2020), was highly abundant in all the green organs studied, which could be explained more by their photosynthetic capacity than by sucrose transport. The peduncles and the sheaths were, clearly, the organs where fructans accumulated (Figure 6), suggesting their predominant storage function. Takahashi et al. (2001) proposed a long-term storage function in peduncles and short-term in sheaths, involved in diel fluctuations. Starch, a minor storage carbohydrate in wheat (Scofield et al., 2009), is accumulated mainly in blades and, later, in ear organs. Glucose and fructose were predominantly abundant in sheaths and peduncles at earlier stages and in ear organs at both growth stages. The free carbohydrates are frequently derivate from the breakdown of other carbohydrates to transport C through the plant (Cimini et al., 2015), which could indicate that sheaths and peduncles provided C at anthesis (e.g. C from blades), and ears at grain filling. The ear is the youngest organ in the plant, so its delayed senescence (Jia et al., 2015; Vicente et al., 2018b; Tambussi et al., 2021) may indicate that ear organs play a more active role at later stages. According to Takahashi et al. (2001), from late grain-filling, any new assimilate is used for grain growth. These results indicated that not only the blades but any of the green organs are actively contributing to canopy photosynthesis with an impact on yield. Previous studies pointed out that the photosynthesis of non-laminar organs, mainly the ears, significantly contributed to canopy photosynthesis and, then, GY (Maydup et al., 2010; Jia et al., 2015; Gámez et al., 2020; Araus et al., 2021b). Gross ear photosynthesis was ~56% of leaf photosynthesis on an area basis (Molero and Reynolds, 2020), while net photosynthesis may be much higher if we subtract the high ear respiration (Gámez et al., 2020; Tambussi et al., 2021) or consider the larger ear area (Olszewski et al., 2018; Sanchez-Bragado et al., 2020b), making ear photosynthesis a promising target for crop improvement.

A previous study suggested that 42% of the N in grains was coming from the ears, using N isotope signatures (Sanchez-Bragado et al., 2017). We combined measurements of N content, isotope composition, and enzyme activities to deepen into N metabolism at the whole plant level. The enzyme profiles revealed active N metabolism functioning in every organ, with higher levels of GS and GOGAT in blades and awns, and GDH in blades and lemmas (Figure 6). It may indicate that an important part of N metabolism takes place outside the blades, corroborating at the biochemical level's previous results (Lopes et al., 2006; Sanchez-Bragado et al., 2017). The high GDH activities in ears and, particularly, in lemmas may suggest an important role in plant glutamate homeostasis, involved in C-N signaling (Labboun et al., 2009) and, given their proximity to grains, in the N supply for grain filling at late stages. Organ-specific N levels followed a similar trend that Rubisco traits (high in blades and awns), mainly due to the fact that Rubisco and other photosynthetic structures require a high N budget (Evans and Clarke, 2019). The rest of the nutrients also had higher levels in blades, but very high levels of Fe in the glumes were observed. Fe is essential for photosynthetic processes, heme biosynthesis, and Fe-S cluster assembly (Morrissey and Guerinot, 2009), but its specific role in glumes remains unclear and should be further investigated.

Lower N fertilization significantly inhibited photosynthetic capacity and N assimilation at the whole plant level, except for an upregulation in the peduncle during anthesis (Supplementary Table 2). It also promoted the storage of C in peduncles, as reported previously in bread wheat (Scofield et al., 2009), while the high decrease of sucrose and starch levels in ear organs at MGF may suggest that either (i) the ears decreased their capacity to supply C to the grain or other organs under low N, or (ii) most of the C produced is sent out due to the high demand of heterotrophic tissues. Our isotopic results and those of Sanchez-Bragado et al. (2017), together with the better activation state of Rubisco at late stages (Figure 7), pointed to the latter.

### Metabolic and Nutrient Changes Between Anthesis and Mid-Grain Filling Point to the Specialization of Each Green Organ in the Later Growth Stages

The clear increase in free carbohydrates at MGF in blades may suggest that different C-rich cellular components are degraded to provide nutrients to other organs (Figure 7). The decrease of fructose and the drastic increase of fructan levels in peduncles at MGF may indicate that this organ is actively accumulating C, which will be probably used when plant photosynthesis ceases at the end of grain filling (Takahashi et al., 2001). These changes were not observed in sheaths, which could support the hypothesis that they participate more in the diurnal accumulation of fructans (Takahashi et al., 2001). Furthermore, CO_2_ fixation by Rubisco was improved at MGF in sheaths, glumes, and, only at control N, in peduncles and lemmas, suggesting a relevant photosynthetic contribution at late stages. The increased Rubisco activity in ears was due to an increase in its activation state, even though protein levels decreased. We hypothesize that these organs may have redistributed efficiently the N stored in this enzyme to other limiting processes. Interestingly, Kanno et al. (2017) observed that rice mutants with lower Rubisco content improved N use efficiency and photosynthesis. While N decreased at MGF in every organ, GDH tended to increase, suggesting that it may act predominantly deaminating glutamate at late stages and, then, reallocating N to the developing grains (Labboun et al., 2009). Low N supply had a clear effect on reducing C assimilation through the observed changes in sucrose levels and Rubisco activation state at the whole plant level, reflecting the strong coordination between C and N metabolism (Vicente et al., 2018a). In general, low N also inhibited PEPCase, GS, and GOGAT activities, probably by limiting their substrate concentrations. In parallel to N, P and Cu also decreased at MGF. The changes in K, Ca, Mg, Fe, and Mn were organ specific. Meanwhile, C decreased in blades and, more significantly, in ear organs, which may suggest a high C contribution of ears at late stages to the developing grains. Overall, the pattern of changes between anthesis and MGF suggested that each organ evolves in a different way, indicating diverse but complementary roles for the control of starch and protein deposition to the grain during the grain-filling phase.

### Linear and Stepwise Regressions Highlight the Key Role of Ear Metabolic Traits and Blade Carbohydrates for Durum Wheat Growth and Productivity

Although correlations do not imply cause-effect relationships, we used them to determine the possible contribution of the different photosynthetic organs to grain filling and to identify key traits (Figure 8). Accumulation of free carbohydrates and lower sucrose, starch, and fructan contents was positively associated with GY, mainly in blades and the different ear organs. It clearly highlighted that higher productivity is linked to rapid translocation of photoassimilates, predominantly for grain filling since plant growth is ceased at late stages (Figure 3). Oppositely, sucrose was positively correlated with GY in ear organs at MGF, suggesting again ears as key C sources for grains. Apart from carbohydrate metabolism, it was surprising that other metabolic traits in blades were not associated with GY (Figure 7). However, GY and biomass were linked to a more active C and N metabolism in awns, glumes, and lemmas, as observed with the concomitant association of Rubisco, PEPCase, and sucrose at late stages with GY. The high contribution of ears to grain filling may be related to its proximity to the grain, delayed senescence, higher light harvesting at the top of the canopy, or even its putative capacity to reassimilate respired CO_2_ (Sanchez-Bragado et al., 2020b; Tambussi et al., 2021). Whether awn metabolism or, particularly, its photosynthetic capacity is relevant for GY has been controversial (Sanchez-Bragado et al., 2020a). Our results do suggest this at the biochemical level. Based on the correlations, the sheath appeared to be an organ that performed functions more oriented for plant growth, while the metabolic traits of the peduncle did not have a considerable impact on yield or biomass, even negative correlations between these parameters were observed (Figure 7). This may be associated with the advantage of shorter varieties (i.e., peduncles or stems), which favors the contribution of ears to grain filling (Tambussi et al., 2021). The only study to our knowledge, comparing leaf and whole-ear photosynthesis with GY, suggested that the latter correlated better than the former (Abbad et al., 2004). We previously found that Rubisco gene expression in durum wheat ears and leaves, as well as several N-metabolism-related genes, was correlated with higher productivity (Vicente et al., 2018b). Moreover, Vergara-Diaz et al. (2020a) proved that leaf, glume, and lemma metabolomes were determinant for GY in durum wheat. Lastly, Shokat et al. (2020) also reported that antioxidant and C metabolism enzymes in leaves and whole ears correlated with yield traits in bread wheat.

The similarity of the isotope compositions between green organs and grains at harvest has been used as a non-intrusive technique to estimate the relative organ contribution to grain filling (Sanchez-Bragado et al., 2014b, 2017; Tambussi et al., 2021). Our models suggested that the supply of C and N to the grains was, to some extent, due to the contribution of the different organs (Figure 9). Moreover, the relative contribution of C from non-foliar organs, in particular the ear organs, stood out above the rest, while, for N, the contribution was more varied in terms of plant parts. Protein content, considered as the most important GQ trait, was mainly associated with the metabolism of lemmas and blades (Figure 8), while the isotope signatures suggested a key role also for glumes (Figure 9).

## Conclusions

We highlight that our novel characterization of key enzymes activities in six different green organs, together with carbohydrate profiles, mineral compositions, natural isotope compositions, and plant canopy monitoring, was an integrative approach to identify metabolic and physiological targets involved in grain filling. The primary metabolism of green organs suggested that all have important functions in contributing to early and late grain filling. Although, in absolute terms, the blades presented the greatest metabolic activity among the green organs, only their carbohydrate metabolism was associated with GY. The pattern of correlations between key enzyme activities and sucrose in ear organs with GY emphasized the key role of ears during grain filling at the metabolic level (Sanchez-Bragado et al., 2014b, 2017; Vicente et al., 2018b; Shokat et al., 2020; Vergara-Diaz et al., 2020b). Our results showed that, regardless of the N supply, high yield was associated with plants with shorter peduncles and longer ears (high sink strength), stay-green phenotype with more photosynthetically active biomass at late-growth stages, better leaf water and N status, and more active ear metabolism, particularly at MGF (i.e., higher Rubisco, PEPCase, GOGAT, and GDH activities). This study opens the doors to investigate on a larger population of varieties of the molecular and morphological mechanisms operating in non-foliar photosynthetic organs that impact GY and GQ. We predict that advances in organ-specific high-throughput phenotyping and metabolic regulation of source-sink dynamics will strongly contribute to crop improvement under optimal and unfavorable environments, highlighting the need of including ear photosynthesis in the breeding programs as a new target for crop improvement.

## Data Availability Statement

The raw data supporting the conclusions of this article will be made available by the authors, without undue reservation.

## Author Contributions

RV, NA, and JA conceived and supervised the project. RM-P and NA performed the field trials. RM-P prepared the samples and carried out most of the measurements, with help from AS, MH, BE, and RV for enzyme assays, MN-T for grain quality traits, and RM for carbohydrates content and grain nutrient composition. RV and RM-P performed the analyses and designed the tables and figures. RV wrote the manuscript with the assistance of RM-P. All the authors provided critical feedback and contributed to the final manuscript.

## Funding

This study was supported by the projects AGL2016-76527-R and PID2019-106650RB-C22, funded by the Spanish Ministry of Science and Innovation, and the projects CSI260P20 and CLU-2019-05-IRNASA/CSIC Unit of Excellence funded by the Junta de Castilla y León and co-financed by the European Union (ERDF). We also acknowledge the support of FCT—Fundaçãopara a Ciência e a Tecnologia, I.P., through the R&D Unit GREEN-IT -Bioresources for Sustainability (UIDB/04551/2020 and UIDP/04551/2020). RMP was the recipient of an FPI-INIA fellowship from the Spanish Ministry of Science and Innovation (CPD2016-0107).

## Conflict of Interest

RM-P and NA were employed by Junta de Castilla y León. The remaining authors declare that the research was conducted in the absence of any commercial or financial relationships that could be construed as a potential conflict of interest.

## Publisher's Note

All claims expressed in this article are solely those of the authors and do not necessarily represent those of their affiliated organizations, or those of the publisher, the editors and the reviewers. Any product that may be evaluated in this article, or claim that may be made by its manufacturer, is not guaranteed or endorsed by the publisher.
