# Supplementary material for: Source-Sink Dynamics in Field-Grown Durum Wheat Under Contrasting Nitrogen Supplies: Key Role of Non-Foliar Organs During Grain Filling
Source: Front Plant Sci. 2022 Apr 29;13:869680. doi: 10.3389/fpls.2022.869680 (PMC9100808; doi:10.3389/fpls.2022.869680)
Supplement: Supplementary file 1 [file Data_Sheet_1.docx]

**Supplementary Table 1.** List of varieties and traits analysed in this study, including the abbreviations and units used.

| **Group** | **Trait** | **Description** | **Units** |
| --- | --- | --- | --- |
| Varieties | HAR | Durum wheat variety Haristide | - |
|  | EUR | Durum wheat variety Euroduro | - |
|  | DRI | Durum wheat variety Don Ricardo | - |
|  | KNI | Durum wheat variety Kiko Nick | - |
| Agronomic components | GY | Grain yield | kg ha^-1^ |
|  | biomass | Biomass | kg ha^-1^ |
|  | HI | Harvest index | g grain g biomass^-1^ |
|  | plants.m2 | Number of plants per area | plants m^-2^ |
|  | ears.plant | Number of ears per plant | ears plant^-1^ |
|  | grains.ear | Number of grains per ear | grains ear^-1^ |
|  | TGW | Thousand grain weight | g |
|  | ped.length | Length of peduncle (last stem internode) | cm |
|  | ear.length | Length of ear | cm |
|  | height | Plant height | cm |
| Conventional grain quality traits | prot.grain | Protein content of grain | % |
|  | moisture.grain | Moisture content of grain | % |
|  | SW.grain | Specific weight of grain | g L^-1^ |
|  | vitreo.grain | Vitreousness of grain | % |
|  | sedim.grain | Sodium dodecyl sulphate (SDS) sedimentation volumes of grain, sedimentation index | mL g^-1^ |
|  | b.grain | CIELAB b* coordinate, yellowness index, yellow colour of semolina | (dimensionless) |
|  | WG.grain | Wet gluten of grain | mg |
|  | GI.grain | Gluten index of grain | % |
| Canopy and leaf phenotyping | GA.15 | Green area index at Zadoks 15 | (dimensionless) |
|  | GA.25 | Green area index at Zadoks 25 | (dimensionless) |
|  | GA.35 | Green area index at Zadoks 35 | (dimensionless) |
|  | GA.55 | Green area index at Zadoks 55 | (dimensionless) |
|  | GA.65 | Green area index at Zadoks 65 | (dimensionless) |
|  | GA.75 | Green area index at Zadoks 75 | (dimensionless) |
|  | GGA.15 | Greener area index at Zadoks 15 | (dimensionless) |
|  | GGA.25 | Greener area index at Zadoks 25 | (dimensionless) |
|  | GGA.35 | Greener area index at Zadoks 35 | (dimensionless) |
|  | GGA.55 | Greener area index at Zadoks 55 | (dimensionless) |
|  | GGA.65 | Greener area index at Zadoks 65 | (dimensionless) |
|  | GGA.75 | Greener area index at Zadoks 75 | (dimensionless) |
|  | CSI.15 | Crop senescence index at Zadoks 15 | (dimensionless) |
|  | CSI.25 | Crop senescence index at Zadoks 25 | (dimensionless) |
|  | CSI.35 | Crop senescence index at Zadoks 35 | (dimensionless) |
|  | CSI.55 | Crop senescence index at Zadoks 55 | (dimensionless) |
|  | CSI.65 | Crop senescence index at Zadoks 65 | (dimensionless) |
|  | CSI.75 | Crop senescence index at Zadoks 75 | (dimensionless) |
|  | NDVI.25 | Normalized difference vegetation index at Zadoks 25 | (dimensionless) |
|  | NDVI.35 | Normalized difference vegetation index at Zadoks 35 | (dimensionless) |
|  | NDVI.55 | Normalized difference vegetation index at Zadoks 55 | (dimensionless) |
|  | NDVI.65 | Normalized difference vegetation index at Zadoks 65 | (dimensionless) |
|  | NDVI.75 | Normalized difference vegetation index at Zadoks 75 | (dimensionless) |
|  | NDVI.85 | Normalized difference vegetation index at Zadoks 85 | (dimensionless) |
|  | chl.65 | Relative chlorophyll content at Zadoks 65 | (dimensionless) |
|  | chl.75 | Relative chlorophyll content at Zadoks 75 | (dimensionless) |
|  | flav.65 | Relative flavonols content at Zadoks 65 | (dimensionless) |
|  | flav.75 | Relative flavonols content at Zadoks 75 | (dimensionless) |
|  | anth.65 | Relative anthocyanins content at Zadoks 65 | (dimensionless) |
|  | anth.75 | Relative anthocyanins content at Zadoks 75 | (dimensionless) |
|  | NBI.65 | Nitrogen balance index at Zadoks 65 | (dimensionless) |
|  | NBI.75 | Nitrogen balance index at Zadoks 75 | (dimensionless) |
|  | LRWC.65 | Leaf relative water content at Zadoks 65 | % |
|  | LRWC.75 | Leaf relative water content at Zadoks 75 | % |
| Weight per organ | FW.b.65 | Fresh weight of flag leaf blade at Zadoks 65 | g |
|  | FW.s.65 | Fresh weight of flag leaf sheath at Zadoks 65 | g |
|  | FW.p.65 | Fresh weight of peduncle at Zadoks 65 | g |
|  | FW.ear.65 | Fresh weight of ear at Zadoks 65 | g |
|  | FW.b.75 | Fresh weight of flag leaf blade at Zadoks 75 | g |
|  | FW.s.75 | Fresh weight of flag leaf sheath at Zadoks 75 | g |
|  | FW.p.75 | Fresh weight of peduncle at Zadoks 75 | g |
|  | FW.ear.75 | Fresh weight of ear at Zadoks 75 | g |

**Supplementary Table 1.** Continued.

| **Group** | **Trait** | **Description** | **Units** |
| --- | --- | --- | --- |
| Weight per organ | DW.b.65 | Dry weight of flag leaf blade at Zadoks 65 | g |
|  | DW.s.65 | Dry weight of flag leaf sheath at Zadoks 65 | g |
|  | DW.p.65 | Dry weight of peduncle at Zadoks 65 | g |
|  | DW.ear.65 | Dry weight of ear at Zadoks 65 | g |
|  | DW.b.75 | Dry weight of flag leaf blade at Zadoks 75 | g |
|  | DW.s.75 | Dry weight of flag leaf sheath at Zadoks 75 | g |
|  | DW.p.75 | Dry weight of peduncle at Zadoks 75 | g |
|  | DW.ear.75 | Dry weight of ear at Zadoks 75 | g |
|  | DW.b.85 | Dry weight of flag leaf blade at Zadoks 85 | g |
|  | DW.s.85 | Dry weight of flag leaf sheath at Zadoks 85 | g |
|  | DW.p.85 | Dry weight of peduncle at Zadoks 85 | g |
|  | DW.ear.85 | Dry weight of ear at Zadoks 85 | g |
| Carbohydrate content per organ | glc.b.65 | Glucose content of flag leaf blade at Zadoks 65 | µmol g FW^-1^ |
|  | glc.s.65 | Glucose content of flag leaf sheath at Zadoks 65 | µmol g FW^-1^ |
|  | glc.p.65 | Glucose content of peduncle at Zadoks 65 | µmol g FW^-1^ |
|  | glc.a.65 | Glucose content of awn at Zadoks 65 | µmol g FW^-1^ |
|  | glc.g.65 | Glucose content of glume at Zadoks 65 | µmol g FW^-1^ |
|  | glc.l.65 | Glucose content of lemma at Zadoks 65 | µmol g FW^-1^ |
|  | glc.b.75 | Glucose content of flag leaf blade at Zadoks 75 | µmol g FW^-1^ |
|  | glc.s.75 | Glucose content of flag leaf sheath at Zadoks 75 | µmol g FW^-1^ |
|  | glc.p.75 | Glucose content of peduncle at Zadoks 75 | µmol g FW^-1^ |
|  | glc.a.75 | Glucose content of awn at Zadoks 75 | µmol g FW^-1^ |
|  | glc.g.75 | Glucose content of glume at Zadoks 75 | µmol g FW^-1^ |
|  | glc.l.75 | Glucose content of lemma at Zadoks 75 | µmol g FW^-1^ |
|  | fru.b.65 | Fructose content of flag leaf blade at Zadoks 65 | µmol g FW^-1^ |
|  | fru.s.65 | Fructose content of flag leaf sheath at Zadoks 65 | µmol g FW^-1^ |
|  | fru.p.65 | Fructose content of peduncle at Zadoks 65 | µmol g FW^-1^ |
|  | fru.a.65 | Fructose content of awn at Zadoks 65 | µmol g FW^-1^ |
|  | fru.g.65 | Fructose content of glume at Zadoks 65 | µmol g FW^-1^ |
|  | fru.l.65 | Fructose content of lemma at Zadoks 65 | µmol g FW^-1^ |
|  | fru.b.75 | Fructose content of flag leaf blade at Zadoks 75 | µmol g FW^-1^ |
|  | fru.s.75 | Fructose content of flag leaf sheath at Zadoks 75 | µmol g FW^-1^ |
|  | fru.p.75 | Fructose content of peduncle at Zadoks 75 | µmol g FW^-1^ |
|  | fru.a.75 | Fructose content of awn at Zadoks 75 | µmol g FW^-1^ |
|  | fru.g.75 | Fructose content of glume at Zadoks 75 | µmol g FW^-1^ |
|  | fru.l.75 | Fructose content of lemma at Zadoks 75 | µmol g FW^-1^ |
|  | suc.b.65 | Sucrose content of flag leaf blade at Zadoks 65 | µmol g FW^-1^ |
|  | suc.s.65 | Sucrose content of flag leaf sheath at Zadoks 65 | µmol g FW^-1^ |
|  | suc.p.65 | Sucrose content of peduncle at Zadoks 65 | µmol g FW^-1^ |
|  | suc.a.65 | Sucrose content of awn at Zadoks 65 | µmol g FW^-1^ |
|  | suc.g.65 | Sucrose content of glume at Zadoks 65 | µmol g FW^-1^ |
|  | suc.l.65 | Sucrose content of lemma at Zadoks 65 | µmol g FW^-1^ |
|  | suc.b.75 | Sucrose content of flag leaf blade at Zadoks 75 | µmol g FW^-1^ |
|  | suc.s.75 | Sucrose content of flag leaf sheath at Zadoks 75 | µmol g FW^-1^ |
|  | suc.p.75 | Sucrose content of peduncle at Zadoks 75 | µmol g FW^-1^ |
|  | suc.a.75 | Sucrose content of awn at Zadoks 75 | µmol g FW^-1^ |
|  | suc.g.75 | Sucrose content of glume at Zadoks 75 | µmol g FW^-1^ |
|  | suc.l.75 | Sucrose content of lemma at Zadoks 75 | µmol g FW^-1^ |
| Carbohydrate content per organ | fructan.b.65 | Fructan content of flag leaf blade at Zadoks 65 | µmol g FW^-1^ |
|  | fructan.s.65 | Fructan content of flag leaf sheath at Zadoks 65 | µmol g FW^-1^ |
|  | fructan.p.65 | Fructan content of peduncle at Zadoks 65 | µmol g FW^-1^ |
|  | fructan.a.65 | Fructan content of awn at Zadoks 65 | µmol g FW^-1^ |
|  | fructan.g.65 | Fructan content of glume at Zadoks 65 | µmol g FW^-1^ |
|  | fructan.l.65 | Fructan content of lemma at Zadoks 65 | µmol g FW^-1^ |
|  | fructan.b.75 | Fructan content of flag leaf blade at Zadoks 75 | µmol g FW^-1^ |
|  | fructan.s.75 | Fructan content of flag leaf sheath at Zadoks 75 | µmol g FW^-1^ |
|  | fructan.p.75 | Fructan content of peduncle at Zadoks 75 | µmol g FW^-1^ |
|  | fructan.a.75 | Fructan content of awn at Zadoks 75 | µmol g FW^-1^ |
|  | fructan.g.75 | Fructan content of glume at Zadoks 75 | µmol g FW^-1^ |
|  | fructan.l.75 | Fructan content of lemma at Zadoks 75 | µmol g FW^-1^ |
|  | starch.b.65 | Starch content of flag leaf blade at Zadoks 65 | µmol g FW^-1^ |
|  | starch.s.65 | Starch content of flag leaf sheath at Zadoks 65 | µmol g FW^-1^ |
|  | starch.p.65 | Starch content of peduncle at Zadoks 65 | µmol g FW^-1^ |
|  | starch.a.65 | Starch content of awn at Zadoks 65 | µmol g FW^-1^ |

**Supplementary Table 1.** Continued.

| **Group** | **Trait** | **Description** | **Units** |
| --- | --- | --- | --- |
| Carbohydrate content per organ | starch.g.65 | Starch content of glume at Zadoks 65 | µmol g FW^-1^ |
|  | starch.l.65 | Starch content of lemma at Zadoks 65 | µmol g FW^-1^ |
|  | starch.b.75 | Starch content of flag leaf blade at Zadoks 75 | µmol g FW^-1^ |
|  | starch.s.75 | Starch content of flag leaf sheath at Zadoks 75 | µmol g FW^-1^ |
|  | starch.p.75 | Starch content of peduncle at Zadoks 75 | µmol g FW^-1^ |
|  | starch.a.75 | Starch content of awn at Zadoks 75 | µmol g FW^-1^ |
|  | starch.g.75 | Starch content of glume at Zadoks 75 | µmol g FW^-1^ |
|  | starch.l.75 | Starch content of lemma at Zadoks 75 | µmol g FW^-1^ |
| Rubisco content per organ | rbcL.b.65 | Rubisco large subunit content of flag leaf blade at Zadoks 65 | mg g FW^-1^ |
|  | rbcL.s.65 | Rubisco large subunit content of flag leaf sheath at Zadoks 65 | mg g FW^-1^ |
|  | rbcL.p.65 | Rubisco large subunit content of peduncle at Zadoks 65 | mg g FW^-1^ |
|  | rbcL.a.65 | Rubisco large subunit content of awn at Zadoks 65 | mg g FW^-1^ |
|  | rbcL.g.65 | Rubisco large subunit content of glume at Zadoks 65 | mg g FW^-1^ |
|  | rbcL.l.65 | Rubisco large subunit content of lemma at Zadoks 65 | mg g FW^-1^ |
|  | rbcL.b.75 | Rubisco large subunit content of flag leaf blade at Zadoks 75 | mg g FW^-1^ |
|  | rbcL.s.75 | Rubisco large subunit content of flag leaf sheath at Zadoks 75 | mg g FW^-1^ |
|  | rbcL.p.75 | Rubisco large subunit content of peduncle at Zadoks 75 | mg g FW^-1^ |
|  | rbcL.a.75 | Rubisco large subunit content of awn at Zadoks 75 | mg g FW^-1^ |
|  | rbcL.g.75 | Rubisco large subunit content of glume at Zadoks 75 | mg g FW^-1^ |
|  | rbcL.l.75 | Rubisco large subunit content of lemma at Zadoks 75 | mg g FW^-1^ |
| Enzyme activities per organ | RCOI.b.65 | Initial Rubisco activity of flag leaf blade at Zadoks 65 | µmol g FW^-1^ min^-1^ |
|  | RCOI.s.65 | Initial Rubisco activity of flag leaf sheath at Zadoks 65 | µmol g FW^-1^ min^-1^ |
|  | RCOI.p.65 | Initial Rubisco activity of peduncle at Zadoks 65 | µmol g FW^-1^ min^-1^ |
|  | RCOI.a.65 | Initial Rubisco activity of awn at Zadoks 65 | µmol g FW^-1^ min^-1^ |
|  | RCOI.g.65 | Initial Rubisco activity of glume at Zadoks 65 | µmol g FW^-1^ min^-1^ |
|  | RCOI.l.65 | Initial Rubisco activity of lemma at Zadoks 65 | µmol g FW^-1^ min^-1^ |
|  | RCOI.b.75 | Initial Rubisco activity of flag leaf blade at Zadoks 75 | µmol g FW^-1^ min^-1^ |
|  | RCOI.s.75 | Initial Rubisco activity of flag leaf sheath at Zadoks 75 | µmol g FW^-1^ min^-1^ |
|  | RCOI.p.75 | Initial Rubisco activity of peduncle at Zadoks 75 | µmol g FW^-1^ min^-1^ |
|  | RCOI.a.75 | Initial Rubisco activity of awn at Zadoks 75 | µmol g FW^-1^ min^-1^ |
|  | RCOI.g.75 | Initial Rubisco activity of glume at Zadoks 75 | µmol g FW^-1^ min^-1^ |
|  | RCOI.l.75 | Initial Rubisco activity of lemma at Zadoks 75 | µmol g FW^-1^ min^-1^ |
|  | RCOT.b.65 | Total Rubisco activity of flag leaf blade at Zadoks 65 | µmol g FW^-1^ min^-1^ |
|  | RCOT.s.65 | Total Rubisco activity of flag leaf sheath at Zadoks 65 | µmol g FW^-1^ min^-1^ |
|  | RCOT.p.65 | Total Rubisco activity of peduncle at Zadoks 65 | µmol g FW^-1^ min^-1^ |
|  | RCOT.a.65 | Total Rubisco activity of awn at Zadoks 65 | µmol g FW^-1^ min^-1^ |
|  | RCOT.g.65 | Total Rubisco activity of glume at Zadoks 65 | µmol g FW^-1^ min^-1^ |
|  | RCOT.l.65 | Total Rubisco activity of lemma at Zadoks 65 | µmol g FW^-1^ min^-1^ |
| Enzyme activities per organ | RCOT.b.75 | Total Rubisco activity of flag leaf blade at Zadoks 75 | µmol g FW^-1^ min^-1^ |
|  | RCOT.s.75 | Total Rubisco activity of flag leaf sheath at Zadoks 75 | µmol g FW^-1^ min^-1^ |
|  | RCOT.p.75 | Total Rubisco activity of peduncle at Zadoks 75 | µmol g FW^-1^ min^-1^ |
|  | RCOT.a.75 | Total Rubisco activity of awn at Zadoks 75 | µmol g FW^-1^ min^-1^ |
|  | RCOT.g.75 | Total Rubisco activity of glume at Zadoks 75 | µmol g FW^-1^ min^-1^ |
|  | RCOT.l.75 | Total Rubisco activity of lemma at Zadoks 75 | µmol g FW^-1^ min^-1^ |
|  | RCOas.b.65 | Rubisco activation state of flag leaf blade at Zadoks 65 | % |
|  | RCOas.s.65 | Rubisco activation state of flag leaf sheath at Zadoks 65 | % |
|  | RCOas.p.65 | Rubisco activation state of peduncle at Zadoks 65 | % |
|  | RCOas.a.65 | Rubisco activation state of awn at Zadoks 65 | % |
|  | RCOas.g.65 | Rubisco activation state of glume at Zadoks 65 | % |
|  | RCOas.l.65 | Rubisco activation state of lemma at Zadoks 65 | % |
|  | RCOas.b.75 | Rubisco activation state of flag leaf blade at Zadoks 75 | % |
|  | RCOas.s.75 | Rubisco activation state of flag leaf sheath at Zadoks 75 | % |
|  | RCOas.p.75 | Rubisco activation state of peduncle at Zadoks 75 | % |
|  | RCOas.a.75 | Rubisco activation state of awn at Zadoks 75 | % |
|  | RCOas.g.75 | Rubisco activation state of glume at Zadoks 75 | % |
|  | RCOas.l.75 | Rubisco activation state of lemma at Zadoks 75 | % |
|  | PEPC.b.65 | Phosphoenolpyruvate carboxylase activity of flag leaf blade at Zadoks 65 | nmol g FW^-1^ min^-1^ |
|  | PEPC.s.65 | Phosphoenolpyruvate carboxylase activity of flag leaf sheath at Zadoks 65 | nmol g FW^-1^ min^-1^ |
|  | PEPC.p.65 | Phosphoenolpyruvate carboxylase activity of peduncle at Zadoks 65 | nmol g FW^-1^ min^-1^ |
|  | PEPC.a.65 | Phosphoenolpyruvate carboxylase activity of awn at Zadoks 65 | nmol g FW^-1^ min^-1^ |
|  | PEPC.g.65 | Phosphoenolpyruvate carboxylase activity of glume at Zadoks 65 | nmol g FW^-1^ min^-1^ |
|  | PEPC.l.65 | Phosphoenolpyruvate carboxylase activity of lemma at Zadoks 65 | nmol g FW^-1^ min^-1^ |

**Supplementary Table 1.** Continued.

| **Group** | **Trait** | **Description** | **Units** |
| --- | --- | --- | --- |
| Enzyme activities per organ | PEPC.b.75 | Phosphoenolpyruvate carboxylase activity of flag leaf blade at Zadoks 75 | nmol g FW^-1^ min^-1^ |
|  | PEPC.s.75 | Phosphoenolpyruvate carboxylase activity of flag leaf sheath at Zadoks 75 | nmol g FW^-1^ min^-1^ |
|  | PEPC.p.75 | Phosphoenolpyruvate carboxylase activity of peduncle at Zadoks 75 | nmol g FW^-1^ min^-1^ |
|  | PEPC.a.75 | Phosphoenolpyruvate carboxylase activity of awn at Zadoks 75 | nmol g FW^-1^ min^-1^ |
|  | PEPC.g.75 | Phosphoenolpyruvate carboxylase activity of glume at Zadoks 75 | nmol g FW^-1^ min^-1^ |
|  | PEPC.l.75 | Phosphoenolpyruvate carboxylase activity of lemma at Zadoks 75 | nmol g FW^-1^ min^-1^ |
|  | GS.b.65 | Glutamine synthetase activity of flag leaf blade at Zadoks 65 | nmol g FW^-1^ min^-1^ |
|  | GS.s.65 | Glutamine synthetase activity of flag leaf sheath at Zadoks 65 | nmol g FW^-1^ min^-1^ |
|  | GS.p.65 | Glutamine synthetase activity of peduncle at Zadoks 65 | nmol g FW^-1^ min^-1^ |
|  | GS.a.65 | Glutamine synthetase activity of awn at Zadoks 65 | nmol g FW^-1^ min^-1^ |
|  | GS.g.65 | Glutamine synthetase activity of glume at Zadoks 65 | nmol g FW^-1^ min^-1^ |
|  | GS.l.65 | Glutamine synthetase activity of lemma at Zadoks 65 | nmol g FW^-1^ min^-1^ |
|  | GS.b.75 | Glutamine synthetase activity of flag leaf blade at Zadoks 75 | nmol g FW^-1^ min^-1^ |
|  | GS.s.75 | Glutamine synthetase activity of flag leaf sheath at Zadoks 75 | nmol g FW^-1^ min^-1^ |
|  | GS.p.75 | Glutamine synthetase activity of peduncle at Zadoks 75 | nmol g FW^-1^ min^-1^ |
|  | GS.a.75 | Glutamine synthetase activity of awn at Zadoks 75 | nmol g FW^-1^ min^-1^ |
|  | GS.g.75 | Glutamine synthetase activity of glume at Zadoks 75 | nmol g FW^-1^ min^-1^ |
|  | GS.l.75 | Glutamine synthetase activity of lemma at Zadoks 75 | nmol g FW^-1^ min^-1^ |
|  | GOGAT.b.65 | Ferredoxin-dependent glutamate synthase activity of flag leaf blade at Zadoks 65 | nmol g FW^-1^ min^-1^ |
|  | GOGAT.s.65 | Ferredoxin-dependent glutamate synthase activity of flag leaf sheath at Zadoks 65 | nmol g FW^-1^ min^-1^ |
|  | GOGAT.p.65 | Ferredoxin-dependent glutamate synthase activity of peduncle at Zadoks 65 | nmol g FW^-1^ min^-1^ |
|  | GOGAT.a.65 | Ferredoxin-dependent glutamate synthase activity of awn at Zadoks 65 | nmol g FW^-1^ min^-1^ |
|  | GOGAT.g.65 | Ferredoxin-dependent glutamate synthase activity of glume at Zadoks 65 | nmol g FW^-1^ min^-1^ |
|  | GOGAT.l.65 | Ferredoxin-dependent glutamate synthase activity of lemma at Zadoks 65 | nmol g FW^-1^ min^-1^ |
|  | GOGAT.b.75 | Ferredoxin-dependent glutamate synthase activity of flag leaf blade at Zadoks 75 | nmol g FW^-1^ min^-1^ |
|  | GOGAT.s.75 | Ferredoxin-dependent glutamate synthase activity of flag leaf sheath at Zadoks 75 | nmol g FW^-1^ min^-1^ |
|  | GOGAT.p.75 | Ferredoxin-dependent glutamate synthase activity of peduncle at Zadoks 75 | nmol g FW^-1^ min^-1^ |
|  | GOGAT.a.75 | Ferredoxin-dependent glutamate synthase activity of awn at Zadoks 75 | nmol g FW^-1^ min^-1^ |
|  | GOGAT.g.75 | Ferredoxin-dependent glutamate synthase activity of glume at Zadoks 75 | nmol g FW^-1^ min^-1^ |
|  | GOGAT.l.75 | Ferredoxin-dependent glutamate synthase activity of lemma at Zadoks 75 | nmol g FW^-1^ min^-1^ |
|  | GDH.b.65 | NADH-dependent glutamate dehydrogenase activity of flag leaf blade at Zadoks 65 | nmol g FW^-1^ min^-1^ |
|  | GDH.s.65 | NADH-dependent glutamate dehydrogenase activity of flag leaf sheath at Zadoks 65 | nmol g FW^-1^ min^-1^ |
|  | GDH.p.65 | NADH-dependent glutamate dehydrogenase activity of peduncle at Zadoks 65 | nmol g FW^-1^ min^-1^ |
|  | GDH.a.65 | NADH-dependent glutamate dehydrogenase activity of awn at Zadoks 65 | nmol g FW^-1^ min^-1^ |
|  | GDH.g.65 | NADH-dependent glutamate dehydrogenase activity of glume at Zadoks 65 | nmol g FW^-1^ min^-1^ |
|  | GDH.l.65 | NADH-dependent glutamate dehydrogenase activity of lemma at Zadoks 65 | nmol g FW^-1^ min^-1^ |
|  | GDH.b.75 | NADH-dependent glutamate dehydrogenase activity of flag leaf blade at Zadoks 75 | nmol g FW^-1^ min^-1^ |
|  | GDH.s.75 | NADH-dependent glutamate dehydrogenase activity of flag leaf sheath at Zadoks 75 | nmol g FW^-1^ min^-1^ |
|  | GDH.p.75 | NADH-dependent glutamate dehydrogenase activity of peduncle at Zadoks 75 | nmol g FW^-1^ min^-1^ |
|  | GDH.a.75 | NADH-dependent glutamate dehydrogenase activity of awn at Zadoks 75 | nmol g FW^-1^ min^-1^ |
|  | GDH.g.75 | NADH-dependent glutamate dehydrogenase activity of glume at Zadoks 75 | nmol g FW^-1^ min^-1^ |
|  | GDH.l.75 | NADH-dependent glutamate dehydrogenase activity of lemma at Zadoks 75 | nmol g FW^-1^ min^-1^ |
| Nutrient composition per organ | C.b.65 | Carbon content of flag leaf blade at Zadoks 65 | g kg DW^-1^ |
|  | C.s.65 | Carbon content of flag leaf sheath at Zadoks 65 | g kg DW^-1^ |
|  | C.p.65 | Carbon content of peduncle at Zadoks 65 | g kg DW^-1^ |
|  | C.a.65 | Carbon content of awn at Zadoks 65 | g kg DW^-1^ |
|  | C.g.65 | Carbon content of glume at Zadoks 65 | g kg DW^-1^ |
|  | C.l.65 | Carbon content of lemma at Zadoks 65 | g kg DW^-1^ |
|  | C.b.75 | Carbon content of flag leaf blade at Zadoks 75 | g kg DW^-1^ |
|  | C.s.75 | Carbon content of flag leaf sheath at Zadoks 75 | g kg DW^-1^ |
|  | C.p.75 | Carbon content of peduncle at Zadoks 75 | g kg DW^-1^ |
|  | C.a.75 | Carbon content of awn at Zadoks 75 | g kg DW^-1^ |
|  | C.g.75 | Carbon content of glume at Zadoks 75 | g kg DW^-1^ |
|  | C.l.75 | Carbon content of lemma at Zadoks 75 | g kg DW^-1^ |
|  | N.b.65 | Nitrogen content of flag leaf blade at Zadoks 65 | g kg DW^-1^ |
|  | N.s.65 | Nitrogen content of flag leaf sheath at Zadoks 65 | g kg DW^-1^ |
|  | N.p.65 | Nitrogen content of peduncle at Zadoks 65 | g kg DW^-1^ |
|  | N.a.65 | Nitrogen content of awn at Zadoks 65 | g kg DW^-1^ |
|  | N.g.65 | Nitrogen content of glume at Zadoks 65 | g kg DW^-1^ |
|  | N.l.65 | Nitrogen content of lemma at Zadoks 65 | g kg DW^-1^ |
|  | N.b.75 | Nitrogen content of flag leaf blade at Zadoks 75 | g kg DW^-1^ |
|  | N.s.75 | Nitrogen content of flag leaf sheath at Zadoks 75 | g kg DW^-1^ |
|  | N.p.75 | Nitrogen content of peduncle at Zadoks 75 | g kg DW^-1^ |
|  | N.a.75 | Nitrogen content of awn at Zadoks 75 | g kg DW^-1^ |
|  | N.g.75 | Nitrogen content of glume at Zadoks 75 | g kg DW^-1^ |
|  | N.l.75 | Nitrogen content of lemma at Zadoks 75 | g kg DW^-1^ |

**Supplementary Table 1.** Continued.

| **Group** | **Trait** | **Description** | **Units** |
| --- | --- | --- | --- |
| Nutrient composition per organ | CN.b.65 | Carbon-to-nitrogen ratio of flag leaf blade at Zadoks 65 | g C g N^-1^ |
|  | CN.s.65 | Carbon-to-nitrogen ratio of flag leaf sheath at Zadoks 65 | g C g N^-1^ |
|  | CN.p.65 | Carbon-to-nitrogen ratio of peduncle at Zadoks 65 | g C g N^-1^ |
|  | CN.a.65 | Carbon-to-nitrogen ratio of awn at Zadoks 65 | g C g N^-1^ |
|  | CN.g.65 | Carbon-to-nitrogen ratio of glume at Zadoks 65 | g C g N^-1^ |
|  | CN.l.65 | Carbon-to-nitrogen ratio of lemma at Zadoks 65 | g C g N^-1^ |
|  | CN.b.75 | Carbon-to-nitrogen ratio of flag leaf blade at Zadoks 75 | g C g N^-1^ |
|  | CN.s.75 | Carbon-to-nitrogen ratio of flag leaf sheath at Zadoks 75 | g C g N^-1^ |
|  | CN.p.75 | Carbon-to-nitrogen ratio of peduncle at Zadoks 75 | g C g N^-1^ |
|  | CN.a.75 | Carbon-to-nitrogen ratio of awn at Zadoks 75 | g C g N^-1^ |
|  | CN.g.75 | Carbon-to-nitrogen ratio of glume at Zadoks 75 | g C g N^-1^ |
|  | CN.l.75 | Carbon-to-nitrogen ratio of lemma at Zadoks 75 | g C g N^-1^ |
|  | K.b.65 | Potassium content of flag leaf blade at Zadoks 65 | g kg DW^-1^ |
|  | K.s.65 | Potassium content of flag leaf sheath at Zadoks 65 | g kg DW^-1^ |
|  | K.p.65 | Potassium content of peduncle at Zadoks 65 | g kg DW^-1^ |
|  | K.a.65 | Potassium content of awn at Zadoks 65 | g kg DW^-1^ |
|  | K.g.65 | Potassium content of glume at Zadoks 65 | g kg DW^-1^ |
|  | K.l.65 | Potassium content of lemma at Zadoks 65 | g kg DW^-1^ |
| Nutrient composition per organ | K.b.75 | Potassium content of flag leaf blade at Zadoks 75 | g kg DW^-1^ |
|  | K.s.75 | Potassium content of flag leaf sheath at Zadoks 75 | g kg DW^-1^ |
|  | K.p.75 | Potassium content of peduncle at Zadoks 75 | g kg DW^-1^ |
|  | K.a.75 | Potassium content of awn at Zadoks 75 | g kg DW^-1^ |
|  | K.g.75 | Potassium content of glume at Zadoks 75 | g kg DW^-1^ |
|  | K.l.75 | Potassium content of lemma at Zadoks 75 | g kg DW^-1^ |
|  | Ca.b.65 | Calcium content of flag leaf blade at Zadoks 65 | g kg DW^-1^ |
|  | Ca.s.65 | Calcium content of flag leaf sheath at Zadoks 65 | g kg DW^-1^ |
|  | Ca.p.65 | Calcium content of peduncle at Zadoks 65 | g kg DW^-1^ |
|  | Ca.a.65 | Calcium content of awn at Zadoks 65 | g kg DW^-1^ |
|  | Ca.g.65 | Calcium content of glume at Zadoks 65 | g kg DW^-1^ |
|  | Ca.l.65 | Calcium content of lemma at Zadoks 65 | g kg DW^-1^ |
|  | Ca.b.75 | Calcium content of flag leaf blade at Zadoks 75 | g kg DW^-1^ |
|  | Ca.s.75 | Calcium content of flag leaf sheath at Zadoks 75 | g kg DW^-1^ |
|  | Ca.p.75 | Calcium content of peduncle at Zadoks 75 | g kg DW^-1^ |
|  | Ca.a.75 | Calcium content of awn at Zadoks 75 | g kg DW^-1^ |
|  | Ca.g.75 | Calcium content of glume at Zadoks 75 | g kg DW^-1^ |
|  | Ca.l.75 | Calcium content of lemma at Zadoks 75 | g kg DW^-1^ |
|  | P.b.65 | Phosphorus content of flag leaf blade at Zadoks 65 | g kg DW^-1^ |
|  | P.s.65 | Phosphorus content of flag leaf sheath at Zadoks 65 | g kg DW^-1^ |
|  | P.p.65 | Phosphorus content of peduncle at Zadoks 65 | g kg DW^-1^ |
|  | P.a.65 | Phosphorus content of awn at Zadoks 65 | g kg DW^-1^ |
|  | P.g.65 | Phosphorus content of glume at Zadoks 65 | g kg DW^-1^ |
|  | P.l.65 | Phosphorus content of lemma at Zadoks 65 | g kg DW^-1^ |
|  | P.b.75 | Phosphorus content of flag leaf blade at Zadoks 75 | g kg DW^-1^ |
|  | P.s.75 | Phosphorus content of flag leaf sheath at Zadoks 75 | g kg DW^-1^ |
|  | P.p.75 | Phosphorus content of peduncle at Zadoks 75 | g kg DW^-1^ |
|  | P.a.75 | Phosphorus content of awn at Zadoks 75 | g kg DW^-1^ |
|  | P.g.75 | Phosphorus content of glume at Zadoks 75 | g kg DW^-1^ |
|  | P.l.75 | Phosphorus content of lemma at Zadoks 75 | g kg DW^-1^ |
|  | Mg.b.65 | Magnesium content of flag leaf blade at Zadoks 65 | g kg DW^-1^ |
|  | Mg.s.65 | Magnesium content of flag leaf sheath at Zadoks 65 | g kg DW^-1^ |
|  | Mg.p.65 | Magnesium content of peduncle at Zadoks 65 | g kg DW^-1^ |
|  | Mg.a.65 | Magnesium content of awn at Zadoks 65 | g kg DW^-1^ |
|  | Mg.g.65 | Magnesium content of glume at Zadoks 65 | g kg DW^-1^ |
|  | Mg.l.65 | Magnesium content of lemma at Zadoks 65 | g kg DW^-1^ |
|  | Mg.b.75 | Magnesium content of flag leaf blade at Zadoks 75 | g kg DW^-1^ |
|  | Mg.s.75 | Magnesium content of flag leaf sheath at Zadoks 75 | g kg DW^-1^ |
|  | Mg.p.75 | Magnesium content of peduncle at Zadoks 75 | g kg DW^-1^ |
|  | Mg.a.75 | Magnesium content of awn at Zadoks 75 | g kg DW^-1^ |
|  | Mg.g.75 | Magnesium content of glume at Zadoks 75 | g kg DW^-1^ |
|  | Mg.l.75 | Magnesium content of lemma at Zadoks 75 | g kg DW^-1^ |
|  | Fe.b.65 | Iron content of flag leaf blade at Zadoks 65 | mg kg DW^-1^ |
|  | Fe.s.65 | Iron content of flag leaf sheath at Zadoks 65 | mg kg DW^-1^ |
|  | Fe.p.65 | Iron content of peduncle at Zadoks 65 | mg kg DW^-1^ |
|  | Fe.a.65 | Iron content of awn at Zadoks 65 | mg kg DW^-1^ |
|  | Fe.g.65 | Iron content of glume at Zadoks 65 | mg kg DW^-1^ |
|  | Fe.l.65 | Iron content of lemma at Zadoks 65 | mg kg DW^-1^ |

**Supplementary Table 1.** Continued.

| **Group** | **Trait** | **Description** | **Units** |
| --- | --- | --- | --- |
| Nutrient composition per organ | Fe.b.75 | Iron content of flag leaf blade at Zadoks 75 | mg kg DW^-1^ |
|  | Fe.s.75 | Iron content of flag leaf sheath at Zadoks 75 | mg kg DW^-1^ |
|  | Fe.p.75 | Iron content of peduncle at Zadoks 75 | mg kg DW^-1^ |
|  | Fe.a.75 | Iron content of awn at Zadoks 75 | mg kg DW^-1^ |
|  | Fe.g.75 | Iron content of glume at Zadoks 75 | mg kg DW^-1^ |
|  | Fe.l.75 | Iron content of lemma at Zadoks 75 | mg kg DW^-1^ |
| Nutrient composition per organ | Mn.b.65 | Manganese content of flag leaf blade at Zadoks 65 | mg kg DW^-1^ |
|  | Mn.s.65 | Manganese content of flag leaf sheath at Zadoks 65 | mg kg DW^-1^ |
|  | Mn.p.65 | Manganese content of peduncle at Zadoks 65 | mg kg DW^-1^ |
|  | Mn.a.65 | Manganese content of awn at Zadoks 65 | mg kg DW^-1^ |
|  | Mn.g.65 | Manganese content of glume at Zadoks 65 | mg kg DW^-1^ |
|  | Mn.l.65 | Manganese content of lemma at Zadoks 65 | mg kg DW^-1^ |
|  | Mn.b.75 | Manganese content of flag leaf blade at Zadoks 75 | mg kg DW^-1^ |
|  | Mn.s.75 | Manganese content of flag leaf sheath at Zadoks 75 | mg kg DW^-1^ |
|  | Mn.p.75 | Manganese content of peduncle at Zadoks 75 | mg kg DW^-1^ |
|  | Mn.a.75 | Manganese content of awn at Zadoks 75 | mg kg DW^-1^ |
|  | Mn.g.75 | Manganese content of glume at Zadoks 75 | mg kg DW^-1^ |
|  | Mn.l.75 | Manganese content of lemma at Zadoks 75 | mg kg DW^-1^ |
|  | Cu.b.65 | Cupper content of flag leaf blade at Zadoks 65 | mg kg DW^-1^ |
|  | Cu.s.65 | Cupper content of flag leaf sheath at Zadoks 65 | mg kg DW^-1^ |
|  | Cu.p.65 | Cupper content of peduncle at Zadoks 65 | mg kg DW^-1^ |
|  | Cu.a.65 | Cupper content of awn at Zadoks 65 | mg kg DW^-1^ |
|  | Cu.g.65 | Cupper content of glume at Zadoks 65 | mg kg DW^-1^ |
|  | Cu.l.65 | Cupper content of lemma at Zadoks 65 | mg kg DW^-1^ |
|  | Cu.b.75 | Cupper content of flag leaf blade at Zadoks 75 | mg kg DW^-1^ |
|  | Cu.s.75 | Cupper content of flag leaf sheath at Zadoks 75 | mg kg DW^-1^ |
|  | Cu.p.75 | Cupper content of peduncle at Zadoks 75 | mg kg DW^-1^ |
|  | Cu.a.75 | Cupper content of awn at Zadoks 75 | mg kg DW^-1^ |
|  | Cu.g.75 | Cupper content of glume at Zadoks 75 | mg kg DW^-1^ |
|  | Cu.l.75 | Cupper content of lemma at Zadoks 75 | mg kg DW^-1^ |
|  | C.grain.75 | Carbon content of grain at Zadoks 75 | g kg DW^-1^ |
|  | C.grain.85 | Carbon content of grain at Zadoks 85 | g kg DW^-1^ |
|  | C.grain.92 | Carbon content of grain at Zadoks 92 | g kg DW^-1^ |
|  | N.grain.75 | Nitrogen content of grain at Zadoks 75 | g kg DW^-1^ |
|  | N.grain.85 | Nitrogen content of grain at Zadoks 85 | g kg DW^-1^ |
|  | N.grain.92 | Nitrogen content of grain at Zadoks 92 | g kg DW^-1^ |
|  | CN.grain.75 | Carbon-to-nitrogen ratio of grain at Zadoks 75 | g C g N^-1^ |
|  | CN.grain.85 | Carbon-to-nitrogen ratio of grain at Zadoks 85 | g C g N^-1^ |
|  | CN.grain.92 | Carbon-to-nitrogen ratio of grain at Zadoks 92 | g C g N^-1^ |
|  | K.grain.92 | Potassium content of grain at Zadoks 92 | g kg DW^-1^ |
|  | P.grain.92 | Phosphorus content of grain at Zadoks 92 | g kg DW^-1^ |
|  | S.grain.92 | Sulphur content of grain at Zadoks 92 | g kg DW^-1^ |
|  | Mg.grain.92 | Magnesium content of grain at Zadoks 92 | g kg DW^-1^ |
|  | Ca.grain.92 | Calcium content of grain at Zadoks 92 | g kg DW^-1^ |
|  | Mn.grain.92 | Manganese content of grain at Zadoks 92 | mg kg DW^-1^ |
|  | Fe.grain.92 | Iron content of grain at Zadoks 92 | mg kg DW^-1^ |
|  | Na.grain.92 | Sodium content of grain at Zadoks 92 | mg kg DW^-1^ |
|  | Zn.grain.92 | Zinc content of grain at Zadoks 92 | mg kg DW^-1^ |
|  | Cu.grain.92 | Cupper content of grain at Zadoks 92 | mg kg DW^-1^ |
|  | Mo.grain.92 | Molybdenum content of grain at Zadoks 92 | mg kg DW^-1^ |
| Nutrient and protein yield | GCY | Grain carbon yield | kg ha^-1^ |
|  | GNY | Grain nitrogen yield | kg ha^-1^ |
|  | GKY | Grain potassium yield | kg ha^-1^ |
|  | GPY | Grain phosphorus yield | kg ha^-1^ |
|  | GSY | Grain sulphur yield | kg ha^-1^ |
|  | GMgY | Grain magnesium yield | kg ha^-1^ |
|  | GCaY | Grain calcium yield | kg ha^-1^ |
|  | GMnY | Grain manganese yield | kg ha^-1^ |
|  | GFeY | Grain iron yield | kg ha^-1^ |
|  | GNaY | Grain sodium yield | kg ha^-1^ |
|  | GZnY | Grain zinc yield | kg ha^-1^ |
|  | GCuY | Grain cupper yield | g ha^-1^ |
|  | GMoY | Grain molybdenum yield | g ha^-1^ |
|  | GProtY | Grain protein yield | kg ha^-1^ |

**Supplementary Table 1.** Continued.

| **Group** | **Trait** | **Description** | **Units** |
| --- | --- | --- | --- |
| Carbon and nitrogen isotope composition per organ | d13C.b.65 | Carbon isotope composition (δ^13^C) of flag leaf blade at Zadoks 65 | ‰ |
|  | d13C.s.65 | Carbon isotope composition (δ^13^C) of flag leaf sheath at Zadoks 65 | ‰ |
|  | d13C.p.65 | Carbon isotope composition (δ^13^C) of peduncle at Zadoks 65 | ‰ |
|  | d13C.a.65 | Carbon isotope composition (δ^13^C) of awn at Zadoks 65 | ‰ |
|  | d13C.g.65 | Carbon isotope composition (δ^13^C) of glume at Zadoks 65 | ‰ |
|  | d13C.l.65 | Carbon isotope composition (δ^13^C) of lemma at Zadoks 65 | ‰ |
|  | d13C.b.75 | Carbon isotope composition (δ^13^C) of flag leaf blade at Zadoks 75 | ‰ |
|  | d13C.s.75 | Carbon isotope composition (δ^13^C) of flag leaf sheath at Zadoks 75 | ‰ |
|  | d13C.p.75 | Carbon isotope composition (δ^13^C) of peduncle at Zadoks 75 | ‰ |
|  | d13C.a.75 | Carbon isotope composition (δ^13^C) of awn at Zadoks 75 | ‰ |
|  | d13C.g.75 | Carbon isotope composition (δ^13^C) of glume at Zadoks 75 | ‰ |
|  | d13C.l.75 | Carbon isotope composition (δ^13^C) of lemma at Zadoks 75 | ‰ |
|  | d13C.grain.75 | Carbon isotope composition (δ^13^C) of grain at Zadoks 75 | ‰ |
|  | d13C.grain.85 | Carbon isotope composition (δ^13^C) of grain at Zadoks 85 | ‰ |
|  | d13C.grain.92 | Carbon isotope composition (δ^13^C) of grain at Zadoks 92 | ‰ |
|  | d15N.b.65 | Nitrogen isotope composition (δ^15^N) of flag leaf blade at Zadoks 65 | ‰ |
|  | d15N.s.65 | Nitrogen isotope composition (δ^15^N) of flag leaf sheath at Zadoks 65 | ‰ |
|  | d15N.p.65 | Nitrogen isotope composition (δ^15^N) of peduncle at Zadoks 65 | ‰ |
|  | d15N.a.65 | Nitrogen isotope composition (δ^15^N) of awn at Zadoks 65 | ‰ |
|  | d15N.g.65 | Nitrogen isotope composition (δ^15^N) of glume at Zadoks 65 | ‰ |
|  | d15N.l.65 | Nitrogen isotope composition (δ^15^N) of lemma at Zadoks 65 | ‰ |
|  | d15N.b.75 | Nitrogen isotope composition (δ^15^N) of flag leaf blade at Zadoks 75 | ‰ |
|  | d15N.s.75 | Nitrogen isotope composition (δ^15^N) of flag leaf sheath at Zadoks 75 | ‰ |
|  | d15N.p.75 | Nitrogen isotope composition (δ^15^N) of peduncle at Zadoks 75 | ‰ |
|  | d15N.a.75 | Nitrogen isotope composition (δ^15^N) of awn at Zadoks 75 | ‰ |
|  | d15N.b.75 | Nitrogen isotope composition (δ^15^N) of glume at Zadoks 75 | ‰ |
|  | d15N.l.75 | Nitrogen isotope composition (δ^15^N) of lemma at Zadoks 75 | ‰ |
|  | d15N.grain75 | Nitrogen isotope composition (δ^15^N) of grain at Zadoks 75 | ‰ |
|  | d15N.grain.85 | Nitrogen isotope composition (δ^15^N) of grain at Zadoks 85 | ‰ |
|  | d15N.grain.92 | Nitrogen isotope composition (δ^15^N) of grain at Zadoks 92 | ‰ |

**Supplementary Table 2.** Effects of N supply and genotypic variability on agronomic, physiological and metabolic traits analysed in field-grown durum wheat. Values are means ± SEM (n = 3) per N (control vs low N) and variety (Kiko Nick, KNI; Don Ricardo, DRI; Euroduro, EUR; Haristide, HAR) combination. The means in each row with different letters differ statistically (*P* < 0.05; two-way ANOVA, TUKEY test). The colour scale indicates the minimum (darkest red) and maximum (darkest blue) values per trait. The abbreviations are described in Supplementary Table 1.

| **Trait** | **Control** | | | | **Low N** | | | | ***P*-value** | | |
| --- | --- | --- | --- | --- | --- | --- | --- | --- | --- | --- | --- |
|  | **KNI** | **DRI** | **EUR** | **HAR** | **KNI** | **DRI** | **EUR** | **HAR** | **N** | **G** | **G×N** |
| GY | 6061 ± 215^ad^ | 6465 ± 773^abc^ | 7130 ± 411^ab^ | 7571 ± 50^a^ | 4227 ± 437^d^ | 5063 ± 282^cd^ | 5394 ± 461^bd^ | 6479 ± 289^abc^ | **<0.001** | **0.003** | 0.804 |
| biomass | 14453 ± 694^a^ | 12973 ± 827^ab^ | 13053 ± 488^ab^ | 15493 ± 653^a^ | 8080 ± 711^c^ | 12467 ± 1266^ab^ | 9907 ± 1144^bc^ | 11680 ± 1058^ac^ | **<0.001** | 0.062 | **0.035** |
| HI | 0.420 ± 0.008^b^ | 0.498 ± 0.046^ab^ | 0.545 ± 0.011^a^ | 0.490 ± 0.019^ab^ | 0.523 ± 0.026^ab^ | 0.410 ± 0.018^b^ | 0.548 ± 0.018^a^ | 0.559 ± 0.025^a^ | 0.222 | **0.004** | **0.006** |
| plants.m2 | 211 ± 20^a^ | 189 ± 15^ab^ | 161 ± 15^ac^ | 215 ± 13^a^ | 161 ± 21^ac^ | 115 ± 7^c^ | 130.7 ± 9^bc^ | 147 ± 5^ac^ | **<0.001** | **0.026** | 0.427 |
| ears.plant | 1.91 ± 0.23^b^ | 1.82 ± 0.14^b^ | 2.20 ± 0.24^b^ | 1.80 ± 0.06^b^ | 1.99 ± 0.17^b^ | 3.05 ± 0.17^a^ | 2.49 ± 0.19^ab^ | 1.90 ± 0.11^b^ | **0.003** | **0.009** | **0.013** |
| grains.ear | 28.3 ± 3.2^ab^ | 31.7 ± 0.7^ab^ | 34.0 ± 2.1^ab^ | 36.1 ± 2.4^a^ | 25.5 ± 1.0^b^ | 31.9 ± 2.5^ab^ | 30.1 ± 2.1^ab^ | 38.1 ± 0.8^a^ | 0.450 | **0.001** | 0.472 |
| TGW | 47.3 ± 2.1^ab^ | 48.9 ± 1.1^ab^ | 45.7 ± 1.3^ab^ | 43.6 ± 0.2^b^ | 48.7 ± 0.2^ab^ | 50.6 ± 1.9^ab^ | 49.9 ± 1.9^ab^ | 52.5 ± 1.7^a^ | **0.001** | 0.530 | 0.074 |
| ped.length | 38.5 ± 1.4^ab^ | 38.9 ± 1.3^a^ | 36.8 ± 0.1^ab^ | 37.2 ± 0.1^ab^ | 34.3 ± 1.5^ab^ | 35.2 ± 0.8^ab^ | 34.9 ± 1.1^ab^ | 34.0 ± 0.2^b^ | **<0.001** | 0.476 | 0.665 |
| ear.length | 5.97 ± 0.32^b^ | 6.53 ± 0.03^b^ | 5.82 ± 0.24^b^ | 9.59 ± 0.12^a^ | 5.07 ± 0.08^b^ | 6.57 ± 0.53^b^ | 6.31 ± 0.76^b^ | 8.83 ± 0.22^a^ | 0.296 | **<0.001** | 0.226 |
| height | 83.8 ± 0.3^a^ | 84.8 ± 1.9^a^ | 81.5 ± 1.9^ab^ | 82.0 ± 1.8^ab^ | 72.0 ± 2.1^b^ | 72.7 ± 3.8^b^ | 75.0 ± 0.6^ab^ | 73.0 ± 2.1^b^ | **<0.001** | 0.940 | 0.495 |
| prot.grain | 14.6 ± 1.7 | 14.3 ± 1.3 | 13.8 ± 1.6 | 13.5 ± 0.9 | 14.1 ± 1.5 | 14.2 ± 0.6 | 13.7 ± 1.1 | 13.4 ± 0.2 | 0.827 | 0.873 | 0.997 |
| moisture.grain | 11.3 ± 0.1^ab^ | 11.0 ± 0.1^ab^ | 11.2 ± 0.2^ab^ | 10.9 ± 0.1^b^ | 11.6 ± 0.1^a^ | 11.3 ± 0.1^ab^ | 11.4 ± 0.1^ab^ | 11.6 ± 0.1^a^ | **0.001** | 0.172 | 0.299 |
| SW.grain | 78.5 ± 0.6^ab^ | 81.1 ± 0.9^ab^ | 82.4 ± 0.5^a^ | 80.5 ± 0.1^ab^ | 78.0 ± 1.8^b^ | 79.8 ± 0.2^ab^ | 81.4 ± 0.8^ab^ | 78.7 ± 0.5^ab^ | 0.065 | **0.004** | 0.883 |
| vitreo.grain | 92.2 ± 5.1 | 97.3 ± 1.5 | 93.0 ± 4.0 | 77.2 ± 16.9 | 79.0 ± 9.1 | 95.8 ± 1.0 | 78.2 ± 6.3 | 69.2 ± 1.2 | 0.097 | 0.050 | 0.813 |
| sedim.grain | 34.3 ± 1.4^ab^ | 30.3 ± 1.3^b^ | 39.3 ± 1.2^a^ | 37.7 ± 1.9^a^ | 14.0 ± 0.3^c^ | 12.9 ± 0.3^c^ | 13.9 ± 0.3^c^ | 14.2 ± 0.1^c^ | **<0.001** | **0.001** | **0.009** |
| b.grain | 15.2 ± 0.1^c^ | 13.9 ± 0.3^c^ | 15.1 ± 0.3^c^ | 16.6 ± 0.1^c^ | 34.7 ± 0.7^a^ | 29.7 ± 0.9^b^ | 37.7 ± 1.8^a^ | 38.7 ± 0.9^a^ | **<0.001** | **<0.001** | **0.003** |
| WG.grain | 27.9 ± 3.8 | 26.6 ± 3.7 | 25.0 ± 5.9 | 26.9 ± 2.9 | 25.5 ± 3.9 | 25.9 ± 1.8 | 27.3 ± 2.9 | 26.2 ± 0.58 | 0.883 | 0.999 | 0.924 |
| GI.grain | 68.5 ± 2.5^ac^ | 70.9 ± 5.0^ab^ | 86.0 ± 4.4^a^ | 40.4 ± 8.8^c^ | 65.2 ± 10.9^ac^ | 68.5 ± 5.0^ac^ | 77.6 ± 2.5^a^ | 44.3 ± 1.4^bc^ | 0.552 | **<0.001** | 0.781 |
| GA.15 | 0.094 ± 0.013 | 0.066 ± 0.004 | 0.085 ± 0.015 | 0.066 ± 0.004 | 0.063 ± 0.024 | 0.070 ± 0.014 | 0.055 ± 0.014 | 0.046 ± 0.012 | 0.070 | 0.462 | 0.564 |
| GA.25 | 0.425 ± 0.050 | 0.306 ± 0.027 | 0.360 ± 0.059 | 0.373 ± 0.024 | 0.319 ± 0.087 | 0.268 ± 0.063 | 0.261 ± 0.062 | 0.273 ± 0.029 | **0.040** | 0.480 | 0.912 |
| GA.35 | 0.709 ± 0.049 | 0.693 ± 0.056 | 0.728 ± 0.055 | 0.679 ± 0.063 | 0.489 ± 0.076 | 0.540 ± 0.066 | 0.508 ± 0.029 | 0.489 ± 0.026 | **<0.001** | 0.911 | 0.913 |
| GA.55 | 0.801 ± 0.042 | 0.801 ± 0.062 | 0.793 ± 0.047 | 0.802 ± 0.047 | 0.615 ± 0.042 | 0.645 ± 0.068 | 0.687 ± 0.040 | 0.645 ± 0.019 | **<0.001** | 0.925 | 0.864 |
| GA.65 | 0.776 ± 0.020 | 0.761 ± 0.048 | 0.811 ± 0.047 | 0.841 ± 0.031 | 0.657 ± 0.062 | 0.717 ± 0.054 | 0.779 ± 0.028 | 0.781 ± 0.024 | **0.046** | 0.115 | 0.737 |
| GA.75 | 0.619 ± 0.064^ac^ | 0.620 ± 0.071^ac^ | 0.702 ± 0.057^ab^ | 0.882 ± 0.017^a^ | 0.353 ± 0.075^c^ | 0.475 ± 0.114^bc^ | 0.472 ± 0.061^bc^ | 0.668 ± 0.025^ac^ | **<0.001** | **0.003** | 0.829 |
| GGA.15 | 0.052 ± 0.008 | 0.036 ± 0.001 | 0.046 ± 0.009 | 0.029 ± 0.003 | 0.037 ± 0.016 | 0.045 ± 0.011 | 0.031 ± 0.008 | 0.022 ± 0.009 | 0.289 | 0.245 | 0.518 |
| GGA.25 | 0.161 ± 0.035 | 0.115 ± 0.014 | 0.142 ± 0.035 | 0.152 ± 0.014 | 0.116 ± 0.043 | 0.094 ± 0.037 | 0.091 ± 0.029 | 0.097 ± 0.021 | 0.059 | 0.712 | 0.939 |
| GGA.35 | 0.491 ± 0.052^ab^ | 0.495 ± 0.078^ab^ | 0.539 ± 0.053^a^ | 0.472 ± 0.076^ab^ | 0.257 ± 0.050^b^ | 0.329 ± 0.065^ab^ | 0.302 ± 0.015^ab^ | 0.276 ± 0.021^ab^ | **<0.001** | 0.758 | 0.905 |
| GGA.55 | 0.667 ± 0.056 | 0.670 ± 0.091 | 0.660 ± 0.062 | 0.675 ± 0.057 | 0.418 ± 0.035 | 0.483 ± 0.080 | 0.530 ± 0.053 | 0.476 ± 0.032 | **<0.001** | 0.857 | 0.811 |
| GGA.65 | 0.577 ± 0.053 | 0.608 ± 0.056 | 0.609 ± 0.070 | 0.649 ± 0.040 | 0.541 ± 0.057 | 0.616 ± 0.052 | 0.657 ± 0.033 | 0.680 ± 0.019 | 0.717 | 0.234 | 0.849 |
| GGA.75 | 0.239 ± 0.061^ab^ | 0.234 ± 0.067^ab^ | 0.256 ± 0.047^ab^ | 0.467 ± 0.029^a^ | 0.116 ± 0.056^b^ | 0.168 ± 0.063^b^ | 0.137 ± 0.038^b^ | 0.305 ± 0.033^ab^ | **0.005** | **0.003** | 0.825 |
| CSI.15 | 45.0 ± 1.2 | 45.5 ± 1.8 | 45.8 ± 2.1 | 56.4 ± 2.9 | 47.5 ± 7.0 | 37.6 ± 3.6 | 44.5 ± 1.0 | 53.6 ± 9.2 | 0.468 | 0.052 | 0.726 |
| CSI.25 | 62.7 ± 3.7 | 62.6 ± 1.4 | 61.9 ± 3.8 | 59.3 ± 1.2 | 66.4 ± 5.4 | 67.3 ± 5.3 | 67.0 ± 4.0 | 65.3 ± 3.6 | 0.091 | 0.900 | 0.992 |
| CSI.35 | 31.2 ± 2.5^ab^ | 29.4 ± 5.2^b^ | 26.3 ± 1.7^b^ | 31.3 ± 4.5^ab^ | 48.0 ± 2.6^a^ | 40.3 ± 5.9^ab^ | 40.5 ± 0.9^ab^ | 43.6 ± 2.3^ab^ | **<0.001** | 0.350 | 0.856 |
| CSI.55 | 17.0 ± 2.9 | 17.2 ± 4.7 | 17.1 ± 3.1 | 16.1 ± 2.3 | 32.2 ± 1.3 | 26.1 ± 5.0 | 23.3 ± 3.3 | 26.4 ± 3.1 | **0.001** | 0.613 | 0.608 |
| CSI.65 | 25.8 ± 5.2 | 20.4 ± 2.6 | 25.4 ± 4.6 | 23.0 ± 2.4 | 17.6 ± 3.4 | 14.1 ± 2.0 | 15.7 ± 1.6 | 12.9 ± 0.5 | **0.001** | 0.451 | 0.925 |
| CSI.75 | 62.5 ± 6.0 | 63.8 ± 8.1 | 63.9 ± 5.0 | 47.1 ± 2.6 | 70.8 ± 9.0 | 67.0 ± 6.2 | 72.0 ± 4.0 | 54.5 ± 4.1 | 0.127 | **0.036** | 0.969 |

**Supplementary Table 2.** Continued.

| **Trait** | **Control** | | | | **Low N** | | | | ***P*-value** | | |
| --- | --- | --- | --- | --- | --- | --- | --- | --- | --- | --- | --- |
|  | **KNI** | **DRI** | **EUR** | **HAR** | **KNI** | **DRI** | **EUR** | **HAR** | **N** | **G** | **G×N** |
| NDVI.25 | 0.540 ± 0.045 | 0.530 ± 0.031 | 0.490 ± 0.031 | 0.500 ± 0.050 | 0.420 ± 0.068 | 0.400 ± 0.031 | 0.423 ± 0.037 | 0.423 ± 0.034 | **0.005** | 0.952 | 0.846 |
| NDVI.35 | 0.633 ± 0.030 | 0.633 ± 0.039 | 0.587 ± 0.032 | 0.607 ± 0.042 | 0.497 ± 0.075 | 0.497 ± 0.049 | 0.510 ± 0.015 | 0.470 ± 0.010 | **0.001** | 0.892 | 0.850 |
| NDVI.55 | 0.733 ± 0.032^ab^ | 0.723 ± 0.038^ab^ | 0.693 ± 0.012^ab^ | 0.767 ± 0.027^a^ | 0.510 ± 0.047^b^ | 0.573 ± 0.087^ab^ | 0.583 ± 0.069^ab^ | 0.527 ± 0.035^b^ | **<0.001** | 0.944 | 0.518 |
| NDVI.65 | 0.667 ± 0.018^ab^ | 0.637 ± 0.023^ab^ | 0.643 ± 0.024^ab^ | 0.713 ± 0.009^a^ | 0.533 ± 0.026^b^ | 0.560 ± 0.067^ab^ | 0.620 ± 0.023^ab^ | 0.633 ± 0.029^ab^ | **0.003** | 0.099 | 0.414 |
| NDVI.75 | 0.583 ± 0.032^ab^ | 0.557 ± 0.037^ab^ | 0.590 ± 0.025^ab^ | 0.690 ± 0.006^a^ | 0.450 ± 0.046^b^ | 0.473 ± 0.033^b^ | 0.577 ± 0.033^ab^ | 0.590 ± 0.021^ab^ | **0.002** | **0.003** | 0.306 |
| NDVI.85 | 0.237 ± 0.027^ab^ | 0.200 ± 0.021^b^ | 0.213 ± 0.032^ab^ | 0.353 ± 0.049^a^ | 0.233 ± 0.038^ab^ | 0.220 ± 0.021^ab^ | 0.223 ± 0.034^ab^ | 0.350 ± 0.010^ab^ | 0.794 | **0.001** | 0.977 |
| chl.65 | 37.8 ± 4.9 | 43.4 ± 1.2 | 46.1 ± 2.7 | 43.8 ± 4.1 | 42.2 ± 2.6 | 42.7 ± 1.7 | 43.2 ± 2.2 | 40.7 ± 2.4 | 0.793 | 0.491 | 0.568 |
| chl.75 | 43.2 ± 2.3 | 43.3 ± 1.7 | 47.8 ± 3.0 | 46.9 ± 3.6 | 43.9 ± 3.7 | 47.0 ± 1.4 | 49.6 ± 4.3 | 45.94 ± 2.38 | 0.545 | 0.380 | 0.876 |
| flav.65 | 1.64 ± 0.08^ac^ | 1.60 ± 0.07^ac^ | 1.47 ± 0.04^bc^ | 1.40 ± 0.05^c^ | 1.73 ± 0.04^a^ | 1.68 ± 0.03^ab^ | 1.56 ± 0.05^ac^ | 1.47 ± 0.02^bc^ | **0.040** | **0.001** | 0.998 |
| flav.75 | 1.63 ± 0.03^ab^ | 1.64 ± 0.09^ab^ | 1.52 ± 0.10^ab^ | 1.42 ± 0.08^b^ | 1.76 ± 0.03^a^ | 1.67 ± 0.02^ab^ | 1.62 ± 0.03^ab^ | 1.42 ± 0.05^b^ | 0.143 | **0.002** | 0.692 |
| anth.65 | 0.113 ± 0.006 | 0.118 ± 0.004 | 0.121 ± 0.009 | 0.113 ± 0.008 | 0.123 ± 0.007 | 0.117 ± 0.005 | 0.111 ± 0.007 | 0.127 ± 0.002 | 0.456 | 0.949 | 0.269 |
| anth.75 | 0.106 ± 0.002 | 0.114 ± 0.006 | 0.082 ± 0.013 | 0.103 ± 0.010 | 0.107 ± 0.015 | 0.095 ± 0.009 | 0.086 ± 0.006 | 0.109 ± 0.006 | 0.751 | 0.067 | 0.519 |
| NBI.65 | 27.4 ± 1.2 | 27.4 ± 2.0 | 31.6 ± 2.6 | 31.5 ± 2.2 | 24.6 ± 2.1 | 25.8 ± 1.4 | 28.0 ± 2.3 | 28.0 ± 1.7 | 0.057 | 0.146 | 0.956 |
| NBI.75 | 26.9 ± 1.1 | 27.1 ± 2.8 | 33.8 ± 2.7 | 35.9 ± 4.5 | 25.0 ± 2.0 | 28.3 ± 1.1 | 30.9 ± 3.2 | 32.9 ± 2.7 | 0.406 | **0.022** | 0.851 |
| LRWC.65 | 68.6 ± 2.7^bcd^ | 65.4 ± 1.5^cd^ | 82.5 ± 0.1^a^ | 79.5 ± 1.5^a^ | 64.6 ± 2.8^cd^ | 62.1 ± 2.9^d^ | 76.7 ± 2.3^ab^ | 74.4 ± 1.7^ac^ | **0.008** | **<0.001** | 0.937 |
| LRWC.75 | 68.9 ± 5.7 | 71.3 ± 4.2 | 68.9 ± 1.2 | 66.3 ± 1.3 | 60.0 ± 2.3 | 57.0 ± 2.6 | 68.0 ± 1.6 | 60.8 ± 1.7 | **0.003** | 0.366 | 0.184 |
| FW.b.65 | 1.67 ± 0.14^ab^ | 1.54 ± 0.09^ab^ | 1.41 ± 0.15^ab^ | 2.01 ± 0.18^a^ | 1.20 ± 0.06^b^ | 1.59 ± 0.14^ab^ | 1.28 ± 0.13^b^ | 1.62 ± 0.10^ab^ | **0.020** | **0.011** | 0.206 |
| FW.s.65 | 2.07 ± 0.19 | 2.11 ± 0.23 | 1.45 ± 0.11 | 1.90 ± 0.24 | 1.86 ± 0.11 | 2.03 ± 0.07 | 1.45 ± 0.10 | 1.91 ± 0.11 | 0.540 | **0.006** | 0.881 |
| FW.p.65 | 5.65 ± 0.44 | 5.14 ± 0.36 | 4.55 ± 0.35 | 5.51 ± 0.63 | 4.92 ± 0.54 | 5.08 ± 0.38 | 4.45 ± 0.33 | 5.16 ± 0.29 | 0.316 | 0.227 | 0.856 |
| FW.ear.65 | 6.95 ± 0.68 | 6.53 ± 0.43 | 5.92 ± 0.29 | 6.18 ± 0.97 | 6.28 ± 0.56 | 7.47 ± 0.21 | 6.13 ± 0.45 | 7.18 ± 0.37 | 0.348 | 0.370 | 0.407 |
| FW.b.75 | 2.09 ± 0.23^ab^ | 2.10 ± 0.19^ab^ | 1.95 ± 0.15^b^ | 2.77 ± 0.16^a^ | 1.55 ± 0.07^b^ | 1.85 ± 0.13^b^ | 1.56 ± 0.08^b^ | 2.03 ± 0.14^ab^ | **<0.001** | **0.003** | 0.448 |
| FW.s.75 | 2.23 ± 0.28 | 2.20 ± 0.12 | 1.91 ± 0.12 | 2.58 ± 0.15 | 2.04 ± 0.08 | 2.33 ± 0.23 | 1.91 ± 0.16 | 2.30 ± 0.16 | 0.500 | **0.046** | 0.651 |
| FW.p.75 | 5.48 ± 0.82 | 5.49 ± 0.48 | 5.42 ± 0.41 | 6.87 ± 0.28 | 5.29 ± 0.77 | 5.88 ± 0.40 | 5.07 ± 0.16 | 6.10 ± 0.39 | 0.532 | 0.111 | 0.737 |
| FW.ear.75 | 19.4 ± 3.0 | 21.4 ± 2.5 | 19.1 ± 1.8 | 20.1 ± 1.3 | 19.4 ± 1.9 | 24.2 ± 2.3 | 20.9 ± 1.9 | 22.4 ± 1.8 | 0.262 | 0.413 | 0.918 |
| DW.b.65 | 0.529 ± 0.048 | 0.580 ± 0.035 | 0.497 ± 0.039 | 0.676 ± 0.061 | 0.445 ± 0.027 | 0.634 ± 0.048 | 0.560 ± 0.126 | 0.562 ± 0.026 | 0.639 | 0.121 | 0.345 |
| DW.s.65 | 0.703 ± 0.106 | 0.910 ± 0.083 | 0.633 ± 0.052 | 0.777 ± 0.069 | 0.826 ± 0.051 | 0.921 ± 0.044 | 0.649 ± 0.035 | 0.821 ± 0.058 | 0.312 | **0.007** | 0.818 |
| DW.p.65 | 1.81 ± 0.18 | 1.48 ± 0.07 | 1.37 ± 0.16 | 1.60 ± 0.06 | 1.79 ± 0.14 | 1.56 ± 0.03 | 1.68 ± 0.07 | 1.54 ± 0.03 | 0.327 | **0.048** | 0.325 |
| DW.ear.65 | 2.57 ± 0.28 | 2.50 ± 0.15 | 2.31 ± 0.10 | 2.30 ± 0.34 | 2.41 ± 0.22 | 2.82 ± 0.16 | 2.33 ± 0.14 | 2.72 ± 0.16 | 0.333 | 0.463 | 0.520 |
| DW.b.75 | 0.674 ± 0.083^ab^ | 0.755 ± 0.062^ab^ | 0.654 ± 0.051^ab^ | 0.851 ± 0.044^a^ | 0.605 ± 0.020^ab^ | 0.768 ± 0.062^ab^ | 0.585 ± 0.030^b^ | 0.782 ± 0.053^ab^ | 0.221 | **0.005** | 0.833 |
| DW.s.75 | 0.963 ± 0.136 | 0.984 ± 0.056 | 0.798 ± 0.087 | 1.035 ± 0.038 | 0.915 ± 0.017 | 1.082 ± 0.102 | 0.847 ± 0.068 | 1.015 ± 0.061 | 0.727 | 0.056 | 0.785 |
| DW.p.75 | 2.38 ± 0.36 | 2.32 ± 0.19 | 2.25 ± 0.22 | 2.93 ± 0.10 | 2.26 ± 0.34 | 2.54 ± 0.19 | 2.21 ± 0.10 | 2.89 ± 0.13 | 0.966 | **0.035** | 0.883 |
| DW.ear.75 | 8.74 ± 1.47 | 9.43 ± 0.98 | 8.46 ± 0.84 | 8.25 ± 0.60 | 9.01 ± 1.01 | 11.36 ± 1.21 | 10.01 ± 0.87 | 9.90 ± 0.90 | 0.078 | 0.460 | 0.851 |
| DW.b.85 | 0.537 ± 0.028 | 0.746 ± 0.119 | 0.881 ± 0.228 | 0.884 ± 0.097 | 0.441 ± 0.007 | 0.637 ± 0.0870 | 0.561 ± 0.0316 | 0.649 ± 0.149 | **0.034** | 0.123 | 0.736 |
| DW.s.85 | 1.47 ± 0.15^ab^ | 1.41 ± 0.13^ab^ | 1.38 ± 0.18^ab^ | 1.86 ± 0.16^a^ | 0.88 ± 0.01^b^ | 1.13 ± 0.05^b^ | 0.97 ± 0.13^b^ | 0.88 ± 0.13^b^ | **<0.001** | 0.411 | 0.080 |
| DW.p.85 | 4.33 ± 0.30^a^ | 3.63 ± 0.18^ab^ | 3.76 ± 0.07^a^ | 4.81 ± 0.31^a^ | 2.11 ± 0.20^c^ | 2.13 ± 0.10^c^ | 2.15 ± 0.25^c^ | 2.35 ± 0.51^bc^ | **<0.001** | 0.080 | 0.267 |
| DW.ear.85 | 13.6 ± 1.2^b^ | 16.2 ± 1.1^ab^ | 14.3 ± 0.8^ab^ | 18.2 ± 1.4^a^ | 12.1 ± 0.7^b^ | 18.5 ± 0.6^a^ | 15.3 ± 0.2^ab^ | 14.9 ± 0.8^ab^ | 0.560 | **0.001** | **0.034** |
| glc.b.65 | 2.86 ± 1.0 | 3.11 ± 0.29 | 2.68 ± 0.02 | 3.69 ± 0.38 | 2.92 ± 0.35 | 3.29 ± 0.81 | 2.37 ± 0.25 | 3.03 ± 0.29 | 0.625 | 0.400 | 0.838 |
| glc.s.65 | 19.7 ± 4.0 | 25.3 ± 18.4 | 7.5 ± 0.2 | 23.4 ± 5.4 | 20.8 ± 8.0 | 20.4 ± 10.9 | 6.7 ± 1.5 | 10.8 ± 0.9 | 0.484 | 0.297 | 0.857 |
| glc.p.65 | 83.8 ± 15.2^ab^ | 105.5 ± 5.1^a^ | 27.8 ± 8.3^b^ | 59.9 ± 24.8^ab^ | 81.6 ± 20.5^ab^ | 76.2 ± 15.5^ab^ | 20.0 ± 13.4^b^ | 70.1 ± 7.3^ab^ | 0.507 | **0.002** | 0.628 |
| glc.a.65 | 30.8 ± 5.8 | 17.9 ± 2.7 | 20.9 ± 1.8 | 18.9 ± 1.1 | 26.5 ± 2.7 | 20.3 ± 4.5 | 20.5 ± 5.1 | 16.9 ± 1.6 | 0.673 | **0.035** | 0.819 |
| glc.g.65 | 21.9 ± 6.5 | 20.2 ± 5.5 | 27.2 ± 3.4 | 22.1 ± 1.3 | 18.6 ± 3.9 | 22.0 ± 5.1 | 23.9 ± 3.8 | 17.2 ± 3.4 | 0.446 | 0.539 | 0.880 |
| glc.l.65 | 30.6 ± 5.5 | 27.9 ± 3.6 | 21.6 ± 1.2 | 29.8 ± 3.1 | 26.1 ± 4.8 | 26.2 ± 3.8 | 20.1 ± 3.5 | 24.4 ± 2.7 | 0.228 | 0.220 | 0.936 |

**Supplementary Table 2.** Continued.

| **Trait** | **Control** | | | | **Low N** | | | | ***P*-value** | | |
| --- | --- | --- | --- | --- | --- | --- | --- | --- | --- | --- | --- |
|  | **KNI** | **DRI** | **EUR** | **HAR** | **KNI** | **DRI** | **EUR** | **HAR** | **N** | **G** | **G×N** |
| glc.b.75 | 5.26 ± 1.14^b^ | 4.71 ± 0.87^b^ | 3.93 ± 0.16^b^ | 18.55 ± 1.29^a^ | 3.99 ± 0.35^b^ | 2.71 ± 0.65^b^ | 1.70 ± 0.50^b^ | 20.00 ± 1.30^a^ | 0.122 | **<0.001** | 0.182 |
| glc.s.75 | 6.99 ± 1.12^b^ | 5.22 ± 1.48^b^ | 5.54 ± 0.34^b^ | 15.95 ± 3.35^a^ | 7.12 ± 0.10^b^ | 5.55 ± 0.20^b^ | 5.45 ± 1.59^b^ | 16.47 ± 2.41^a^ | 0.856 | **<0.001** | 0.998 |
| glc.p.75 | 2.48 ± 0.28 | 4.12 ± 0.72 | 5.42 ± 1.66 | 6.12 ± 0.60 | 3.30 ± 0.54 | 5.51 ± 1.73 | 8.69 ± 3.22 | 8.93 ± 0.07 | 0.064 | **0.022** | 0.820 |
| glc.a.75 | 15.6 ± 1.4 | 11.8 ± 2.4 | 18.7 ± 1.7 | 13.5 ± 1.2 | 8.2 ± 2.3 | 8.9 ± 3.7 | 14.1 ± 3.7 | 15.3 ± 1.1 | 0.069 | 0.096 | 0.313 |
| glc.g.75 | 13.6 ± 2.7 | 19.4 ± 5.6 | 24.7 ± 2.7 | 11.7 ± 1.1 | 9.3 ± 1.4 | 16.8 ± 5.7 | 16.7 ± 3.3 | 10.8 ± 1.8 | 0.125 | **0.032** | 0.763 |
| glc.l.75 | 13.8 ± 1.7 | 14.4 ± 4.0 | 18.3 ± 1.2 | 16.2 ± 1.5 | 8.8 ± 2.6 | 12.8 ± 4.1 | 13.6 ± 3.1 | 14.8 ± 1.0 | 0.113 | 0.314 | 0.849 |
| fru.b.65 | 5.24 ± 1.06 | 4.62 ± 1.15 | 7.29 ± 1.43 | 5.88 ± 1.30 | 3.39 ± 0.53 | 3.32 ± 0.87 | 6.64 ± 1.55 | 6.65 ± 0.84 | 0.359 | **0.047** | 0.691 |
| fru.s.65 | 13.7 ± 3.3 | 22.2 ± 17.2 | 8.0 ± 1.0 | 20.7 ± 4.3 | 13.5 ± 5.1 | 17.6 ± 9.2 | 7.4 ± 0.7 | 14.5 ± 2.0 | 0.590 | 0.404 | 0.971 |
| fru.p.65 | 50.7 ± 9.8^ab^ | 79.1 ± 6.8^a^ | 19.4 ± 6.1^b^ | 37.8 ± 15.1^ab^ | 47.2 ± 11.5^ab^ | 48.6 ± 16.0^ab^ | 10.1 ± 5.0^b^ | 43.9 ± 5.9^ab^ | 0.222 | **0.002** | 0.367 |
| fru.a.65 | 6.43 ± 0.87^ab^ | 6.22 ± 0.58^ab^ | 10.67 ± 0.23^ab^ | 8.37 ± 0.60^ab^ | 5.47 ± 0.44^b^ | 6.19 ± 0.62^ab^ | 10.86 ± 1.79^ab^ | 11.87 ± 2.51^a^ | 0.440 | **0.001** | 0.307 |
| fru.g.65 | 8.3 ± 1.8^bc^ | 7.6 ± 1.0^c^ | 25.1 ± 1.2^a^ | 15.1 ± 0.6^b^ | 7.6 ± 1.0^c^ | 7.5 ± 0.4^c^ | 24.0 ± 1.8^a^ | 13.1 ± 2.9^bc^ | 0.396 | **<0.001** | 0.936 |
| fru.l.65 | 17.1 ± 1.8^ab^ | 18.3 ± 1.6^ab^ | 23.5 ± 1.2^a^ | 21.7 ± 1.9^ab^ | 14.8 ± 2.2^b^ | 16.5 ± 2.0^ab^ | 20.7 ± 1.9^ab^ | 20.6 ± 0.2^ab^ | 0.117 | **0.007** | 0.962 |
| fru.b.75 | 6.20 ± 0.64^b^ | 4.95 ± 1.00^b^ | 4.49 ± 0.36^b^ | 16.45 ± 1.15^a^ | 6.30 ± 1.60^b^ | 5.33 ± 0.74^b^ | 2.64 ± 0.33^b^ | 19.74 ± 0.99^a^ | 0.480 | **<0.001** | 0.091 |
| fru.s.75 | 8.94 ± 0.28^b^ | 6.01 ± 1.01^b^ | 6.21 ± 0.86^b^ | 40.22 ± 5.32^a^ | 13.25 ± 1.15^b^ | 8.88 ± 0.25^b^ | 7.67 ± 0.38^b^ | 44.77 ± 1.83^a^ | 0.041 | <0.001 | 0.872 |
| fru.p.75 | 2.23 ± 0.42^b^ | 3.27 ± 0.13^b^ | 4.24 ± 0.74^b^ | 11.75 ± 1.08^ab^ | 8.86 ± 4.19^b^ | 6.13 ± 1.19^b^ | 6.54 ± 1.30^b^ | 21.9 ± 3.68^a^ | **0.002** | **<0.001** | 0.256 |
| fru.a.75 | 7.28 ± 0.83^cd^ | 5.13 ± 0.62^d^ | 9.46 ± 1.27^bcd^ | 12.32 ± 0.79^ab^ | 6.76 ± 0.32^d^ | 5.04 ± 0.90^d^ | 11.88 ± 1.21^ac^ | 15.16 ± 1.49^a^ | 0.118 | **<0.001** | 0.261 |
| fru.g.75 | 11.7 ± 1.5 | 12.8 ± 1.2 | 18.7 ± 2.9 | 11.0 ± 0.4 | 11.2 ± 1.1 | 12.9 ± 1.2 | 15.5 ± 1.3 | 11.9 ± 1.8 | 0.566 | **0.008** | 0.601 |
| fru.l.75 | 14.1 ± 1.0^ac^ | 13.8 ± 1.1^ac^ | 18.4 ± 1.2^a^ | 12.9 ± 0.4^bc^ | 9.8 ± 1.1^c^ | 13.1 ± 1.5^bc^ | 15.8 ± 0.9^ab^ | 12.2 ± 1.1^bc^ | **0.015** | **0.001** | 0.314 |
| suc.b.65 | 118 ± 11^bc^ | 122 ± 7^ab^ | 82 ± 7^cd^ | 72 ± 8^d^ | 158 ± 3^a^ | 127 ± 4^ab^ | 117 ± 13^bc^ | 91 ± 2^bd^ | **<0.001** | **<0.001** | 0.162 |
| suc.s.65 | 135 ± 28^ab^ | 190 ± 30^a^ | 131 ± 8^ac^ | 50 ± 11^c^ | 182 ± 11^a^ | 183 ± 18^a^ | 140 ± 3^a^ | 55 ± 3^bc^ | 0.280 | **<0.001** | 0.453 |
| suc.p.65 | 197 ± 67^ab^ | 41 ± 5^c^ | 109 ± 46^bc^ | 40 ± 11^c^ | 283 ± 18^a^ | 62 ± 13^bc^ | 55 ± 4^bc^ | 40 ± 1^c^ | 0.532 | **<0.001** | 0.178 |
| suc.a.65 | 91.2 ± 4.2^a^ | 108.1 ± 4.0^a^ | 65.9 ± 5.2^b^ | 46.3 ± 0.2^b^ | 100.1 ± 7.3^a^ | 97.9 ± 3.9^a^ | 65.9 ± 3.4^b^ | 49.5 ± 1.2^b^ | 0.878 | **<0.001** | 0.184 |
| suc.g.65 | 56.5 ± 8.8^ac^ | 80.7 ± 3.6^a^ | 43.8 ± 3.3^c^ | 43.1 ± 4.9^c^ | 68.6 ± 6.1^ac^ | 74.7 ± 6.1^ab^ | 49.8 ± 0.3^bc^ | 48.0 ± 4.9^c^ | 0.270 | **<0.001** | 0.412 |
| suc.l.65 | 84.3 ± 1.1^ab^ | 97.0 ± 3.9^a^ | 57.6 ± 4.3^cd^ | 45.0 ± 5.2^d^ | 90.7 ± 2.1^a^ | 91.0 ± 5.2^a^ | 69.6 ± 2.3^bc^ | 43.2 ± 1.2^d^ | 0.306 | **<0.001** | 0.088 |
| suc.b.75 | 114 ± 12^abc^ | 122 ± 1^abc^ | 110 ± 3^bd^ | 133 ± 5^ab^ | 106 ± 13^bd^ | 85 ± 6^cd^ | 74 ± 11^d^ | 152 ± 6^a^ | **0.013** | **<0.001** | **0.009** |
| suc.s.75 | 152 ± 1^a^ | 159 ± 14^a^ | 147 ± 7^a^ | 92 ± 4^b^ | 133 ± 14^ab^ | 132 ± 5^ab^ | 125 ± 11^ab^ | 90 ± 9^b^ | **0.017** | **<0.001** | 0.590 |
| suc.p.75 | 190 ± 5^a^ | 92 ± 4^bc^ | 85 ± 8^bc^ | 109 ± 8^bc^ | 149 ± 36^ab^ | 101 ± 5^bc^ | 79 ± 4^c^ | 102 ± 1^bc^ | 0.267 | **<0.001** | 0.356 |
| suc.a.75 | 78.4 ± 6.1^a^ | 80.3 ± 4.4^a^ | 82.8 ± 3.5^a^ | 75.4 ± 4.1^a^ | 47.1 ± 3.5^b^ | 46.9 ± 1.7^b^ | 47.1 ± 6.1^b^ | 79.7 ± 0.6^a^ | **<0.001** | **0.008** | **0.001** |
| suc.g.75 | 53.7 ± 5.5^bd^ | 57.9 ± 1.4^bc^ | 56.3 ± 0.9^bc^ | 70.1 ± 2.0^ab^ | 45.0 ± 0.9^cd^ | 41.4 ± 1.2^cd^ | 36.8 ± 0.1^d^ | 85.3 ± 9.1^a^ | **0.016** | **<0.001** | **0.002** |
| suc.l.75 | 78.0 ± 4.0^b^ | 77.1 ± 0.6^bc^ | 77.6 ± 2.5^bc^ | 86.9 ± 3.9^ab^ | 61.7 ± 1.1^d^ | 61.1 ± 1.1^d^ | 64.7 ± 3.0^cd^ | 98.7 ± 3.1^a^ | **0.001** | **<0.001** | **<0.001** |
| fructan.b.65 | 124 ± 21^ab^ | 175 ± 29^a^ | 57 ± 18^ab^ | 27 ± 8^b^ | 157 ± 32^ab^ | 175 ± 39^a^ | 112 ± 43^ab^ | 51 ± 12^ab^ | 0.170 | **0.001** | 0.798 |
| fructan.s.65 | 320 ± 48 | 261 ± 44 | 331 ± 60 | 254 ± 69 | 164 ± 33 | 290 ± 55 | 330 ± 79 | 397 ± 62 | 0.929 | 0.389 | 0.121 |
| fructan.p.65 | 66 ± 5^b^ | 61 ± 9^b^ | 215 ± 76^ab^ | 76 ± 30^b^ | 205 ± 56^ab^ | 129 ± 32^ab^ | 299 ± 55^a^ | 132 ± 33^ab^ | **0.012** | **0.007** | 0.780 |
| fructan.a.65 | 77.4 ± 15.5 | 104.1 ± 25.0 | 70.2 ± 17.0 | 21.6 ± 1.2 | 68.6 ± 18.6 | 82.6 ± 23.5 | 91.2 ± 27.4 | 37.9 ± 13.7 | 0.898 | **0.025** | 0.655 |
| fructan.g.65 | 148.7 ± 10.8 | 145.2 ± 38.6 | 88.8 ± 20.9 | 60.1 ± 9.4 | 146.1 ± 19.9 | 120.4 ± 35.8 | 93.0 ± 23.6 | 89.2 ± 38.0 | 0.938 | 0.050 | 0.798 |
| fructan.l.65 | 86.9 ± 15.8 | 101.7 ± 22.7 | 63.9 ± 8.8 | 22.4 ± 5.1 | 78.2 ± 18.3 | 78.6 ± 25.0 | 55.4 ± 14.0 | 39.3 ± 9.8 | 0.618 | **0.010** | 0.675 |
| fructan.b.75 | 66 ± 24 | 143 ± 65 | 69 ± 4 | 47 ± 3 | 180 ± 28 | 156 ± 75 | 106 ± 40 | 68 ± 3 | 0.122 | 0.149 | 0.578 |
| fructan.s.75 | 252 ± 36 | 338 ± 27 | 300 ± 27 | 346 ± 40 | 260 ± 24 | 338 ± 49 | 336 ± 35 | 367 ± 12 | 0.497 | **0.039** | 0.947 |
| fructan.p.75 | 576 ± 77^ab^ | 638 ± 93^ab^ | 475 ± 39^b^ | 771 ± 27^ab^ | 570 ± 28^ab^ | 617 ± 59^ab^ | 550 ± 94^ab^ | 798 ± 36^a^ | 0.676 | **0.003** | 0.873 |
| fructan.a.75 | 55.8 ± 5.9^ac^ | 69.6 ± 18.5^a^ | 67.6 ± 8.3^ab^ | 13.4 ± 0.97^c^ | 33.7 ± 5.3^ac^ | 62.9 ± 10.0^ac^ | 64.5 ± 15.5^ab^ | 18.8 ± 3.8^bc^ | 0.369 | **<0.001** | 0.602 |
| fructan.g.75 | 38.3 ± 5.9 | 55.8 ± 18 | 56.8 ± 5.6 | 26.9 ± 3.9 | 36.0 ± 6.2 | 38.7 ± 8.4 | 42.7 ± 13.9 | 43.2 ± 7.1 | 0.537 | 0.370 | 0.337 |
| fructan.l.75 | 38.9 ± 3.2 | 55.7 ± 19.9 | 52.0 ± 3.4 | 18.5 ± 3.1 | 28.8 ± 5.6 | 44.0 ± 10.9 | 42.6 ± 10.1 | 24.9 ± 6.3 | 0.370 | **0.032** | 0.757 |

**Supplementary Table 2.** Continued.

| **Trait** | **Control** | | | | **Low N** | | | | ***P*-value** | | |
| --- | --- | --- | --- | --- | --- | --- | --- | --- | --- | --- | --- |
|  | **KNI** | **DRI** | **EUR** | **HAR** | **KNI** | **DRI** | **EUR** | **HAR** | **N** | **G** | **G×N** |
| starch.b.65 | 15.12 ± 3.60^bc^ | 16.28 ± 1.22^bc^ | 2.96 ± 0.82^d^ | 3.53 ± 0.26^d^ | 32.41 ± 2.01^a^ | 23.85 ± 1.57^ab^ | 8.20 ± 2.11^cd^ | 7.24 ± 1.10^cd^ | **<0.001** | **<0.001** | **0.009** |
| starch.s.65 | 4.59 ± 0.33^ac^ | 6.30 ± 0.89^ab^ | 2.05 ± 0.36^c^ | 1.99 ± 0.35^c^ | 5.62 ± 1.30^ac^ | 7.00 ± 0.78^a^ | 4.32 ± 1.36^ac^ | 2.89 ± 0.32^bc^ | 0.051 | **<0.001** | 0.771 |
| starch.p.65 | 3.47 ± 0.82^ab^ | 2.29 ± 0.34^ab^ | 1.47 ± 0.12^b^ | 2.01 ± 0.36^ab^ | 4.13 ± 0.32^a^ | 3.66 ± 0.41^ab^ | 2.00 ± 0.53^ab^ | 3.17 ± 0.49^ab^ | **0.012** | **0.004** | 0.772 |
| starch.a.65 | 12.50 ± 0.96^a^ | 17.75 ± 2.06^a^ | 3.26 ± 0.74^b^ | 6.35 ± 0.16^b^ | 14.44 ± 1.63^a^ | 16.62 ± 1.55^a^ | 4.44 ± 0.88^b^ | 6.11 ± 0.90^b^ | 0.626 | **<0.001** | 0.614 |
| starch.g.65 | 7.77 ± 0.20^a^ | 8.33 ± 1.31^a^ | 2.95 ± 0.51^b^ | 5.15 ± 0.32^ab^ | 8.28 ± 0.76^a^ | 7.96 ± 0.82^a^ | 3.80 ± 0.47^b^ | 5.54 ± 0.13^ab^ | 0.476 | **<0.001** | 0.831 |
| starch.l.65 | 11.7 ± 1.0^bc^ | 18.4 ± 1.0^a^ | 8.3 ± 0.6^c^ | 12.0 ± 1.7^bc^ | 13.6 ± 0.3^ac^ | 17.3 ± 2.2^ab^ | 10.0 ± 0.6^c^ | 10.5 ± 0.9^c^ | 0.782 | **<0.001** | 0.354 |
| starch.b.75 | 8.57 ± 1.65^b^ | 11.61 ± 2.36^b^ | 5.60 ± 0.30^b^ | 12.59 ± 2.57^ab^ | 10.15 ± 1.96^b^ | 6.68 ± 1.35^b^ | 5.37 ± 1.26^b^ | 20.49 ± 1.83^a^ | 0.405 | **<0.001** | **0.019** |
| starch.s.75 | 2.31 ± 0.47^ab^ | 3.52 ± 0.89^a^ | 2.17 ± 0.18^ab^ | 1.42 ± 0.09^ab^ | 0.94 ± 0.06^b^ | 2.49 ± 0.58^ab^ | 2.11 ± 0.47^ab^ | 2.22 ± 0.12^ab^ | 0.211 | **0.036** | 0.109 |
| starch.p.75 | 1.13 ± 0.53 | 1.01 ± 0.07 | 0.84 ± 0.09 | 0.80 ± 0.11 | 1.35 ± 0.18 | 1.10 ± 0.59 | 0.84 ± 0.30 | 0.62 ± 0.10 | 0.891 | 0.368 | 0.934 |
| starch.a.75 | 4.15 ± 0.46^bd^ | 7.81 ± 0.59^a^ | 4.94 ± 0.60^bc^ | 4.66 ± 0.50^bc^ | 2.28 ± 0.31^d^ | 4.02 ± 0.49^bd^ | 2.74 ± 0.45^cd^ | 6.06 ± 0.34^ab^ | **<0.001** | **<0.001** | **0.001** |
| starch.g.75 | 2.04 ± 0.27^bd^ | 2.72 ± 0.21^b^ | 2.65 ± 0.31^bc^ | 2.84 ± 0.06^b^ | 1.38 ± 0.34^d^ | 1.40 ± 0.16^d^ | 1.49 ± 0.11^cd^ | 4.34 ± 0.37^a^ | **0.035** | **<0.001** | **<0.001** |
| starch.l.75 | 7.85 ± 2.66^a^ | 5.68 ± 0.46^ab^ | 7.04 ± 1.48^ab^ | 3.22 ± 0.21^ab^ | 2.28 ± 0.24^b^ | 3.72 ± 0.33^ab^ | 3.69 ± 0.23^ab^ | 3.66 ± 0.36^ab^ | **0.004** | 0.355 | 0.091 |
| rbcL.b.65 | 10.45 ± 0.70^a^ | 5.95 ± 1.08^bc^ | 6.65 ± 0.89^ac^ | 9.86 ± 0.59^ab^ | 8.70 ± 1.05^ab^ | 4.37 ± 0.71^c^ | 4.15 ± 0.07^c^ | 6.15 ± 0.84^bc^ | **0.001** | **<0.001** | 0.547 |
| rbcL.s.65 | 2.97 ± 0.49 | 1.67 ± 0.16 | 2.23 ± 0.31 | 3.15 ± 0.72 | 2.40 ± 0.05 | 1.61 ± 0.10 | 2.48 ± 0.18 | 2.87 ± 0.62 | 0.568 | **0.022** | 0.779 |
| rbcL.p.65 | 1.63 ± 0.24^ac^ | 0.76 ± 0.18^c^ | 0.64 ± 0.26^c^ | 2.52 ± 0.58^ab^ | 2.64 ± 0.09^ab^ | 1.78 ± 0.43^ac^ | 3.00 ± 0.48^a^ | 1.18 ± 0.09^bc^ | **0.006** | 0.123 | **0.001** |
| rbcL.a.65 | 6.54 ± 0.15^a^ | 4.19 ± 0.34^ab^ | 3.50 ± 0.23^b^ | 5.47 ± 0.29^ab^ | 5.95 ± 0.59^ab^ | 5.48 ± 0.64^ab^ | 3.54 ± 0.49^b^ | 3.82 ± 0.86^b^ | 0.528 | **0.001** | 0.061 |
| rbcL.g.65 | 1.82 ± 0.07^ab^ | 1.76 ± 0.18^ac^ | 1.01 ± 0.12^c^ | 1.39 ± 0.16^ac^ | 1.18 ± 0.05^bc^ | 2.12 ± 0.25^a^ | 1.54 ± 0.21^ac^ | 1.22 ± 0.10^bc^ | 0.846 | **0.002** | **0.008** |
| rbcL.l.65 | 2.56 ± 0.07^b^ | 2.91 ± 0.18^ab^ | 2.22 ± 0.37^b^ | 2.42 ± 0.27^b^ | 2.37 ± 0.30^b^ | 3.13 ± 0.03^ab^ | 3.03 ± 0.03^ab^ | 4.22 ± 0.61^a^ | **0.006** | **0.043** | **0.022** |
| rbcL.b.75 | 13.64 ± 0.66^a^ | 3.66 ± 0.21^e^ | 9.42 ± 0.30^b^ | 7.03 ± 1.36^bd^ | 6.92 ± 0.31^bd^ | 4.04 ± 0.73^de^ | 5.40 ± 0.25^cde^ | 7.94 ± 0.15^bc^ | **<0.001** | **<0.001** | **<0.001** |
| rbcL.s.75 | 6.93 ± 1.17^a^ | 1.59 ± 0.45^b^ | 2.78 ± 0.19^b^ | 4.01 ± 0.34^b^ | 3.55 ± 0.46^b^ | 2.93 ± 0.16^b^ | 1.79 ± 0.51^b^ | 2.17 ± 0.58^b^ | **0.008** | **<0.001** | **0.006** |
| rbcL.p.75 | 1.35 ± 0.07^ac^ | 0.67 ± 0.07^c^ | 1.87 ± 0.33^a^ | 1.02 ± 0.25^ac^ | 1.63 ± 0.02^ab^ | 0.54 ± 0.08^c^ | 0.58 ± 0.18^c^ | 0.98 ± 0.19^bc^ | **0.034** | **0.001** | **0.003** |
| rbcL.a.75 | 3.82 ± 0.16 | 3.33 ± 0.40 | 3.59 ± 0.38 | 3.23 ± 0.77 | 2.83 ± 0.46 | 3.62 ± 0.92 | 3.69 ± 0.88 | 2.69 ± 0.06 | 0.501 | 0.693 | 0.686 |
| rbcL.g.75 | 1.70 ± 0.08^ac^ | 1.02 ± 0.13^ac^ | 0.78 ± 0.23^bc^ | 2.17 ± 0.57^a^ | 1.28 ± 0.09^ac^ | 0.88 ± 0.02^bc^ | 0.66 ± 0.24^c^ | 1.94 ± 0.27^ab^ | 0.230 | **<0.001** | 0.939 |
| rbcL.l.75 | 1.35 ± 0.11 | 1.01 ± 0.16 | 1.24 ± 0.35 | 1.22 ± 0.32 | 1.21 ± 0.11 | 1.44 ± 0.08 | 1.39 ± 0.13 | 1.52 ± 0.16 | 0.213 | 0.901 | 0.538 |
| RCOI.b.65 | 20.5 ± 8.0^ab^ | 19.9 ± 1.1^ab^ | 22.2 ± 1.4^ab^ | 14.1 ± 0.7^b^ | 23.3 ± 0.7^a^ | 22.2 ± 2.1^ab^ | 23.2 ± 2.9^a^ | 20.0 ± 2.5^ab^ | **0.026** | **0.023** | 0.554 |
| RCOI.s.65 | 5.50 ± 0.63^ab^ | 8.85 ± 0.50^a^ | 6.95 ± 1.07^ab^ | 6.03 ± 0.31^ab^ | 4.55 ± 0.23^b^ | 4.42 ± 0.79^b^ | 7.41 ± 1.21^ab^ | 4.28 ± 0.33^b^ | **0.005** | **0.019** | **0.025** |
| RCOI.p.65 | 1.81 ± 0.39^c^ | 1.68 ± 0.25^c^ | 3.45 ± 0.37^bc^ | 3.46 ± 0.21^bc^ | 4.96 ± 0.69^ab^ | 3.19 ± 0.52^bc^ | 6.01 ± 0.95^a^ | 1.92 ± 0.17^c^ | **0.001** | **0.001** | **0.001** |
| RCOI.a.65 | 11.9 ± 7.2 | 11.3 ± 0.8 | 8.79 ± 0.63 | 10.9 ± 0.7 | 11.1± 1.3 | 12.6 ± 0.9 | 9.8 ± 0.9 | 9.5 ± 0.8 | 0.997 | **0.027** | 0.334 |
| RCOI.g.65 | 3.08 ± 0.19^ab^ | 2.50 ± 0.26^ab^ | 2.10 ± 0.33^ab^ | 3.19 ± 0.30^a^ | 2.65 ± 0.08^ab^ | 2.78 ± 0.48^ab^ | 1.75 ± 0.34^b^ | 2.50 ± 0.09^ab^ | 0.163 | **0.015** | 0.404 |
| RCOI.l.65 | 2.65 ± 0.22 | 2.56 ± 0.17 | 1.89 ± 0.22 | 2.65 ± 0.48 | 2.36 ± 0.27 | 2.38 ± 0.14 | 2.30 ± 0.34 | 3.12 ± 0.29 | 0.629 | 0.091 | 0.437 |
| RCOI.b.75 | 21.5 ± 1.2^ab^ | 22.9 ± 04^a^ | 22.9 ± 2.7^a^ | 13.6 ± 1.5^b^ | 13.8 ± 1.6^b^ | 16.5 ± 2.5^ab^ | 20.0 ± 1.5^ab^ | 14.3 ± 0.6^b^ | **0.004** | **0.003** | 0.096 |
| RCOI.s.75 | 9.38 ± 0.77^a^ | 6.17 ± 0.68^ac^ | 7.84 ± 0.56^ac^ | 8.06 ± 0.51^ab^ | 6.43 ± 1.07^ac^ | 6.33 ± 0.32^ac^ | 4.48 ± 0.16^c^ | 5.77 ± 1.17^bc^ | **0.001** | 0.104 | 0.114 |
| RCOI.p.75 | 4.72 ± 0.24^a^ | 3.41 ± 0.12^ab^ | 3.40 ± 0.38^ab^ | 2.49 ± 0.41^bc^ | 3.59 ± 0.41^ab^ | 2.56 ± 0.27^bc^ | 3.95 ± 0.57^ab^ | 1.55 ± 0.20^c^ | **0.030** | **<0.001** | 0.108 |
| RCOI.a.75 | 9.36 ± 0.27^ab^ | 9.77 ± 1.14^a^ | 9.06 ± 1.44^ab^ | 7.01 ± 0.31^ab^ | 4.87 ± 0.72^b^ | 7.23 ± 1.06^ab^ | 7.59 ± 1.43^ab^ | 7.63 ± 0.34^ab^ | **0.010** | 0.393 | 0.099 |
| RCOI.g.75 | 3.66 ± 0.34^ac^ | 3.13 ± 0.06^ac^ | 2.77 ± 0.37^bc^ | 4.99 ± 0.89^a^ | 1.76 ± 0.14^c^ | 2.74 ± 0.19^bc^ | 2.13 ± 0.27^bc^ | 3.99 ± 0.66^ab^ | **0.007** | **0.001** | 0.388 |
| RCOI.l.75 | 2.57 ± 0.19^bc^ | 2.42 ± 0.27^bd^ | 2.74 ± 0.11^ab^ | 3.69 ± 0.15^a^ | 1.72 ± 0.30^cd^ | 2.32 ± 0.08^bd^ | 1.51 ± 0.29^d^ | 2.84 ± 0.14^ab^ | **<0.001** | **<0.001** | 0.084 |
| RCOT.b.65 | 24.3 ± 0.8^ab^ | 23.9 ± 1.3^ab^ | 25.7 ± 3.0^ab^ | 16.7 ± 0.6^b^ | 28.2 ± 2.4^a^ | 24.4 ± 1.6^ab^ | 25.0 ± 3.0^ab^ | 21.3 ± 2.6^ab^ | 0.189 | **0.015** | 0.542 |
| RCOT.s.65 | 6.13 ± 0.45^b^ | 10.99 ± 0.36^a^ | 8.55 ± 1.52^ab^ | 6.72 ± 0.10^b^ | 5.55 ± 0.26^b^ | 5.19 ± 0.81^b^ | 8.60 ± 1.61^ab^ | 4.93 ± 0.30^b^ | **0.005** | **0.008** | **0.017** |
| RCOT.p.65 | 2.69 ± 0.12^c^ | 2.40 ± 0.37^c^ | 4.72 ± 0.46^ac^ | 3.93 ± 0.13^bc^ | 6.48 ± 0.45^ab^ | 3.84 ± 0.64^bc^ | 7.37 ± 1.24^a^ | 2.32 ± 0.11^c^ | **0.001** | **<0.001** | **0.001** |
| RCOT.a.65 | 14.8 ± 0.4^a^ | 13.4 ± 0.7^ab^ | 11.3 ± 1.1^ab^ | 13.8 ± 0.5^ab^ | 12.7 ± 1.1^ab^ | 13.8 ± 0.8^ab^ | 10.5 ± 0.9^b^ | 11.4 ± 0.6^ab^ | 0.053 | **0.010** | 0.318 |
| RCOT.g.65 | 3.40 ± 0.16 | 3.26 ± 0.33 | 2.59 ± 0.41 | 3.57 ± 0.33 | 3.08 ± 0.05 | 3.23 ± 0.05 | 2.09 ± 0.39 | 3.00 ± 0.09 | 0.133 | **0.025** | 0.837 |
| RCOT.l.65 | 3.37 ± 0.18 | 3.53 ± 0.12 | 2.64 ± 0.21 | 3.23 ± 0.51 | 3.15 ± 0.27 | 3.06 ± 0.17 | 3.05 ± 0.37 | 3.35 ± 0.20 | 0.851 | 0.344 | 0.449 |

**Supplementary Table 2.** Continued.

| **Trait** | **Control** | | | | **Low N** | | | | ***P*-value** | | |
| --- | --- | --- | --- | --- | --- | --- | --- | --- | --- | --- | --- |
|  | **KNI** | **DRI** | **EUR** | **HAR** | **KNI** | **DRI** | **EUR** | **HAR** | **N** | **G** | **G×N** |
| RCOT.b.75 | 26.3 ± 1.5 | 25.6 ± 0.2 | 26.7 ± 4.1 | 17.0 ± 2.2 | 18.2 ± 2.5 | 20.2 ± 3.8 | 23.9 ± 3.1 | 17.8 ± 0.6 | 0.052 | 0.051 | 0.401 |
| RCOT.s.75 | 10.81 ± 0.96^a^ | 7.10 ± 0.97^ab^ | 9.56 ± 0.35^ab^ | 9.48 ± 0.70^ab^ | 8.02 ± 1.07^ab^ | 7.79 ± 0.53^ab^ | 5.71 ± 0.14^b^ | 7.09 ± 1.3^ab^ | **0.003** | 0.121 | 0.080 |
| RCOT.p.75 | 5.65 ± 0.44^a^ | 4.13 ± 0.18^ab^ | 4.46 ± 0.46^ab^ | 2.72 ± 0.31^bc^ | 5.15 ± 0.28^a^ | 3.48 ± 0.41^ac^ | 5.41 ± 0.87^a^ | 1.58 ± 0.28^c^ | 0.307 | **<0.001** | 0.155 |
| RCOT.a.75 | 12.14 ± 0.37^a^ | 9.85 ± 1.37^ab^ | 11.02 ± 1.07^a^ | 9.23 ± 0.64^ab^ | 5.98 ± 0.67^b^ | 8.33 ± 0.83^ab^ | 8.14 ± 1.27^ab^ | 8.71 ± 0.23^ab^ | **<0.001** | 0.902 | **0.031** |
| RCOT.g.75 | 3.82 ± 0.35^ac^ | 3.61 ± 0.22^ac^ | 3.17 ± 0.52^bc^ | 5.58 ± 0.83^a^ | 1.87 ± 0.09^c^ | 3.29 ± 0.14^bc^ | 2.58 ± 0.20^bc^ | 4.21 ± 0.39^ab^ | **0.002** | **<0.001** | 0.215 |
| RCOT.l.75 | 3.23 ± 0.12^ac^ | 2.96 ± 0.43^ac^ | 3.71 ± 0.38^ab^ | 4.08 ± 0.02^a^ | 2.13 ± 0.30^c^ | 2.60 ± 0.10^bc^ | 2.00 ± 0.38^c^ | 3.12 ± 0.14^ac^ | **<0.001** | **0.017** | 0.154 |
| RCOas.b.65 | 84.4 ± 2.8 | 83.5 ± 4.2 | 87.8 ± 5.1 | 84.7 ± 5.8 | 83.3 ± 5.3 | 91.0 ± 2.6 | 93.0 ± 2.2 | 94.0 ± 1.2 | 0.079 | 0.392 | 0.590 |
| RCOas.s.65 | 89.3 ± 3.6 | 80.5 ± 2.7 | 82.3 ± 3.2 | 89.6 ± 3.5 | 81.9 ± 0.8 | 85.1 ± 5.7 | 87.2 ± 3.4 | 86.7 ± 1.5 | 0.940 | 0.469 | 0.226 |
| RCOas.p.65 | 67.5 ± 4.5^b^ | 70.3 ± 1.1^ab^ | 72.9 ± 3.4^ab^ | 88.2 ± 5.9^a^ | 75.9 ± 5.9^ab^ | 83.1 ± 0.7^ab^ | 81.9 ± 2.1^ab^ | 82.3 ± 3.4^ab^ | **0.043** | **0.023** | 0.122 |
| RCOas.a.65 | 80.7 ± 3.5 | 84.2 ± 1.2 | 78.5 ± 7.5 | 79.5 ± 5.4 | 86.7 ± 2.7 | 90.6 ± 1.4 | 92.9 ± 0.9 | 82.7 ± 3.7 | **0.015** | 0.432 | 0.540 |
| RCOas.g.65 | 90.7 ± 4.2^a^ | 76.8 ± 1.4^b^ | 80.7 ± 0.7^ab^ | 89.6 ± 4.0^a^ | 86.0 ± 2.0^ab^ | 85.8 ± 2.1^ab^ | 83.7 ± 0.6^ab^ | 83.3 ± 0.6^ab^ | 0.882 | **0.026** | **0.018** |
| RCOas.l.65 | 78.6 ± 2.5^b^ | 72.6 ± 2.9^b^ | 71.3 ± 3.1^b^ | 81.4 ± 2.4^ab^ | 74.6 ± 2.3^b^ | 77.8 ± 0.9^b^ | 75.0 ± 4.5^b^ | 92.7 ± 3.0^a^ | 0.060 | **0.001** | 0.103 |
| RCOas.b.75 | 81.7 ± 0.2 | 89.4 ± 2.2 | 87.1 ± 4.6 | 80.3 ± 3.7 | 76.6 ± 3.1 | 83.4 ± 6.3 | 84.6 ± 4.3 | 80.8 ± 1.8 | 0.233 | 0.164 | 0.824 |
| RCOas.s.75 | 86.9 ± 0.8 | 87.5 ± 4.2 | 82.1 ± 5.1 | 85.2 ± 1.3 | 79.5 ± 3.9 | 81.4 ± 1.5 | 78.5 ± 2.1 | 80.7 ± 3.1 | **0.026** | 0.597 | 0.931 |
| RCOas.p.75 | 84.0 ± 2.2^ac^ | 82.6 ± 0.9^ac^ | 76.2 ± 1.7^bc^ | 90.1 ± 5.0^ab^ | 69.7 ± 6.5^c^ | 73.6 ± 0.9^bc^ | 73.4 ± 1.5^bc^ | 99.7 ± 4.7^a^ | 0.121 | **<0.001** | **0.022** |
| RCOas.a.75 | 77.2 ± 2.3^b^ | 99.9 ± 4.6^a^ | 81.5 ± 6.0^b^ | 76.2 ± 2.1^b^ | 80.7 ± 3.1^b^ | 86.1 ± 4.3^ab^ | 92.4 ± 3.3^ab^ | 87.5 ± 1.7^ab^ | 0.259 | **0.008** | **0.011** |
| RCOas.g.75 | 95.7 ± 1.1 | 87.3 ± 3.8 | 88.2 ± 2.9 | 88.7 ± 4.1 | 94.4 ± 6.3 | 83.1 ± 5.0 | 81.9 ± 4.6 | 93.6 ± 7.2 | 0.619 | 0.138 | 0.667 |
| RCOas.l.75 | 79.3 ± 3.3^ab^ | 82.4 ± 3.3^ab^ | 75.0 ± 5.3^b^ | 90.3 ± 3.2^ab^ | 79.7 ± 3.1^ab^ | 89.2 ± 1.6^ab^ | 75.3 ± 0.7^ab^ | 91.0 ± 3.7^a^ | 0.391 | **0.001** | 0.707 |
| PEPC.b.65 | 1624 ± 133 | 1319 ± 73 | 1690 ± 110 | 1599 ± 157 | 1695 ± 95 | 1347 ± 159 | 1802 ± 11 | 1805 ± 17 | 0.194 | **0.006** | 0.863 |
| PEPC.s.65 | 753 ± 42 | 777 ± 100 | 840 ± 121 | 880 ± 47 | 760 ± 84 | 626 ± 62 | 800 ± 78 | 844 ± 48 | 0.330 | 0.214 | 0.772 |
| PEPC.p.65 | 694 ± 46^ab^ | 502 ± 17^b^ | 705 ± 33^ab^ | 629 ± 67^ab^ | 618 ± 17^ab^ | 652 ± 24^ab^ | 775 ± 97^a^ | 563 ± 46^ab^ | 0.594 | **0.022** | 0.109 |
| PEPC.a.65 | 1104 ± 153 | 956 ± 31 | 1057 ± 64 | 1337 ± 179 | 880 ± 60 | 993 ± 35 | 1054 ± 94 | 949 ± 104 | 0.065 | 0.380 | 0.177 |
| PEPC.g.65 | 818 ± 37^ab^ | 678 ± 34^b^ | 848 ± 12^ab^ | 958 ± 104^a^ | 728 ± 89^ab^ | 682 ± 18^ab^ | 758 ± 14^ab^ | 712 ± 60^ab^ | **0.018** | 0.076 | 0.215 |
| PEPC.l.65 | 759 ± 74 | 769 ± 21 | 830 ± 93 | 917 ± 152 | 694 ± 14 | 775 ± 27 | 990 ± 43 | 974 ± 114 | 0.502 | **0.045** | 0.579 |
| PEPC.b.75 | 1924 ± 97 | 1377 ± 35 | 1732 ± 263 | 1623 ± 180 | 1730 ± 291 | 1630 ± 123 | 1514 ± 88 | 1803 ± 145 | 0.965 | 0.325 | 0.417 |
| PEPC.s.75 | 1048 ± 80^a^ | 937 ± 93^ab^ | 807 ± 100^ab^ | 890 ± 102^ab^ | 813 ± 24^ab^ | 739 ± 71^ab^ | 564 ± 53^b^ | 905 ± 108^ab^ | **0.012** | **0.043** | 0.390 |
| PEPC.p.75 | 1028 ± 84^a^ | 783 ± 104^ab^ | 734 ± 46^ab^ | 676 ± 72^b^ | 706 ± 29^b^ | 570 ± 41^b^ | 732 ± 71^ab^ | 522 ± 14^b^ | **0.002** | **0.005** | 0.132 |
| PEPC.a.75 | 1467 ± 165^a^ | 1246 ± 62^ab^ | 1294 ± 119^ab^ | 1241 ± 113^ab^ | 818 ± 41^b^ | 1339 ± 146^ab^ | 1026 ± 122^ab^ | 1550 ± 73^a^ | 0.125 | 0.123 | **0.003** |
| PEPC.g.75 | 764 ± 25^ab^ | 742 ± 23^ab^ | 719 ± 58^ab^ | 819 ± 99^a^ | 484 ± 55^b^ | 575 ± 25^ab^ | 789 ± 111^ab^ | 719 ± 81^ab^ | **0.024** | 0.128 | 0.111 |
| PEPC.l.75 | 1101 ± 57 | 881 ± 51 | 877 ± 27 | 895 ± 107 | 775 ± 132 | 948 ± 29 | 765 ± 60 | 871 ± 42 | 0.071 | 0.419 | 0.079 |
| GS.b.65 | 3205 ± 203^ab^ | 3918 ± 156^a^ | 3592 ± 353^a^ | 2245 ± 29^b^ | 3777 ± 220^a^ | 3621 ± 375^a^ | 3516 ± 293^ab^ | 2856 ± 314^ab^ | 0.297 | **0.002** | 0.256 |
| GS.s.65 | 782 ± 52^bcd^ | 1455 ± 37^a^ | 1224 ± 101^ab^ | 895 ± 114^bcd^ | 790 ± 44^bcd^ | 756 ± 19^cd^ | 1058 ± 187^ac^ | 594 ± 85^d^ | **0.001** | **0.001** | **0.012** |
| GS.p.65 | 468 ± 33^cd^ | 386 ± 46^cd^ | 629 ± 46^bc^ | 568 ± 42^bc^ | 773 ± 25^ab^ | 577 ± 83^bc^ | 1019 ± 82^a^ | 223 ± 18^d^ | **0.002** | **<0.001** | **<0.001** |
| GS.a.65 | 1870 ± 49^ab^ | 2045 ± 105^a^ | 1563 ± 138^ac^ | 1169 ± 59^c^ | 1931 ± 117^ab^ | 2048 ± 135^a^ | 1425 ± 65^bc^ | 1210 ± 168^c^ | 0.921 | **<0.001** | 0.811 |
| GS.g.65 | 506 ± 10^b^ | 527 ± 65^b^ | 395 ± 52^b^ | 512 ± 56^b^ | 492 ± 28^b^ | 508 ± 62^b^ | 299 ± 46^b^ | 805 ± 21^a^ | 0.235 | **<0.001** | **0.004** |
| GS.l.65 | 516 ± 77^ab^ | 457 ± 36^ab^ | 346 ± 37^b^ | 626 ± 34^a^ | 447 ± 77^ab^ | 452 ± 54^ab^ | 420 ± 23^ab^ | 522 ± 39^ab^ | 0.482 | **0.014** | 0.348 |
| GS.b.75 | 3390 ± 294^ab^ | 3935 ± 85^a^ | 4011 ± 179^a^ | 2556 ± 121^bc^ | 3099 ± 161^ab^ | 3673 ± 374^a^ | 3716 ± 210^a^ | 1989 ± 207^c^ | **0.038** | **<0.001** | 0.889 |
| GS.s.75 | 1342 ± 155^a^ | 959 ± 73^ac^ | 1116 ± 36.9^ab^ | 984 ± 65^ac^ | 767 ± 92^bc^ | 993 ± 52^ac^ | 749 ± 79^bc^ | 623 ± 24^c^ | **<0.001** | **0.046** | **0.013** |
| GS.p.75 | 677 ± 30^a^ | 619 ± 8^a^ | 572 ± 58^a^ | 321 ± 13^b^ | 644 ± 24^a^ | 576 ± 17^a^ | 736 ± 118^a^ | 175 ± 21^b^ | 0.678 | **<0.001** | **0.043** |
| GS.a.75 | 1688 ± 75^a^ | 1768 ± 185^a^ | 1885 ± 107^a^ | 534 ± 57^b^ | 754 ± 147^b^ | 1488 ± 109^a^ | 1538 ± 108^a^ | 581 ± 102^b^ | **<0.001** | **<0.001** | **0.006** |
| GS.g.75 | 695 ± 42^ab^ | 729 ± 55^ab^ | 507 ± 74^b^ | 711 ± 86^ab^ | 489 ± 39^bc^ | 850 ± 36^a^ | 235 ± 46^c^ | 602 ± 29^ab^ | **0.008** | **<0.001** | **0.012** |
| GS.l.75 | 481 ± 29^ab^ | 485 ± 77^ab^ | 520 ± 39^ab^ | 388 ± 90^b^ | 426 ± 11^ab^ | 401 ± 90^b^ | 365 ± 44^b^ | 710 ± 30^a^ | 0.872 | 0.241 | **0.004** |

**Supplementary Table 2.** Continued.

| **Trait** | **Control** | | | | **Low N** | | | | ***P*-value** | | |
| --- | --- | --- | --- | --- | --- | --- | --- | --- | --- | --- | --- |
|  | **KNI** | **DRI** | **EUR** | **HAR** | **KNI** | **DRI** | **EUR** | **HAR** | **N** | **G** | **G×N** |
| GOGAT.b.65 | 2351 ± 141^b^ | 3761 ± 180^a^ | 2775 ± 90^b^ | 2207 ± 87^b^ | 2821 ± 199^b^ | 3877 ± 240^a^ | 2941 ± 232^b^ | 2212 ± 75^b^ | 0.129 | **<0.001** | 0.563 |
| GOGAT.s.65 | 907 ± 66^b^ | 1412 ± 101^a^ | 926 ± 174^ab^ | 804 ± 85^b^ | 654 ± 63^b^ | 463 ± 79^b^ | 932 ± 119^ab^ | 611 ± 89^b^ | **<0.001** | 0.102 | **0.002** |
| GOGAT.p.65 | 474 ± 41^bd^ | 285 ± 69^d^ | 593 ± 25^abc^ | 362 ± 4^cd^ | 660 ± 72^ab^ | 809 ± 73^a^ | 572 ± 46^abc^ | 312 ± 47^d^ | **0.001** | **0.001** | **<0.001** |
| GOGAT.a.65 | 1550 ± 68^a^ | 1389 ± 30^ab^ | 1462 ± 48^ab^ | 1480 ± 171^ab^ | 1484 ± 122^ab^ | 1447 ± 71^ab^ | 1071 ± 89^b^ | 1212 ± 36^ab^ | **0.020** | 0.077 | 0.102 |
| GOGAT.g.65 | 301 ± 66^b^ | 394 ± 74^b^ | 326 ± 23^b^ | 506 ± 60^b^ | 387 ± 27^b^ | 355 ± 20^b^ | 387 ± 10^b^ | 1097 ± 83^a^ | **<0.001** | **<0.001** | **<0.001** |
| GOGAT.l.65 | 407 ± 17^ab^ | 543 ± 51^a^ | 413 ± 23^ab^ | 434 ± 23^ab^ | 364 ± 34^ab^ | 546 ± 55^a^ | 510 ± 38^a^ | 284 ± 59^b^ | 0.428 | **0.001** | 0.050 |
| GOGAT.b.75 | 2794 ± 226 | 3931 ± 276 | 2970 ± 92 | 2290 ± 268 | 2352 ± 389 | 4049 ± 822 | 2418 ± 186 | 2742 ± 67 | 0.687 | **0.003** | 0.493 |
| GOGAT.s.75 | 1194 ± 151^a^ | 1084 ± 76^ab^ | 925 ± 84^ab^ | 1161 ± 121^a^ | 1159 ± 106^a^ | 976 ± 42^ab^ | 682 ± 44^b^ | 916 ± 89^ab^ | **0.033** | **0.011** | 0.632 |
| GOGAT.p.75 | 541 ± 51^a^ | 566 ± 80^a^ | 462 ± 71^ab^ | 230 ± 49^bc^ | 595 ± 43^a^ | 452 ± 9^ab^ | 558 ± 67^a^ | 147 ± 26^c^ | 0.764 | **<0.001** | 0.192 |
| GOGAT.a.75 | 1205 ± 116^ab^ | 1226 ± 149^ab^ | 1345 ± 130^ab^ | 1575 ± 151^a^ | 736 ± 138^b^ | 1442 ± 141^a^ | 1055 ± 158^ab^ | 1630 ± 24^a^ | 0.211 | **0.002** | 0.073 |
| GOGAT.g.75 | 490 ± 54^b^ | 380 ± 26^bc^ | 452 ± 58^b^ | 751 ± 62^a^ | 194 ± 34^c^ | 324 ± 87^bc^ | 373 ± 21^bc^ | 468 ± 29^b^ | **<0.001** | **<0.001** | 0.051 |
| GOGAT.l.75 | 554 ± 11^ab^ | 578 ± 82^a^ | 560 ± 76^ab^ | 550 ± 16^ab^ | 440 ± 26^ab^ | 576 ± 2^a^ | 378 ± 30^ab^ | 356 ± 19^b^ | **0.001** | **0.049** | 0.141 |
| GDH.b.65 | 481 ± 47^ab^ | 593 ± 51^ab^ | 476 ± 74^ab^ | 353 ± 10^b^ | 500 ± 79^ab^ | 723 ± 62^a^ | 670 ± 77^a^ | 497 ± 63^ab^ | **0.013** | **0.010** | 0.561 |
| GDH.s.65 | 331 ± 2^b^ | 381 ± 47^ab^ | 315 ± 23^b^ | 481 ± 28^a^ | 308 ± 18^b^ | 351 ± 17^b^ | 329 ± 15^b^ | 287 ± 33^b^ | **0.006** | 0.057 | **0.005** |
| GDH.p.65 | 239 ± 10^ce^ | 187 ± 6^e^ | 274 ± 3^bcd^ | 357 ± 22^a^ | 220 ± 12^de^ | 301 ± 13^ac^ | 258 ± 25^cd^ | 343 ± 10^ab^ | 0.120 | **<0.001** | **0.001** |
| GDH.a.65 | 295 ± 33^ab^ | 315 ± 10^ab^ | 288 ± 31^ab^ | 370 ± 15^a^ | 243 ± 2^b^ | 288 ± 14^ab^ | 350 ± 36^ab^ | 366 ± 13^a^ | 0.767 | **0.004** | 0.112 |
| GDH.g.65 | 290 ± 10^c^ | 404 ± 38^bc^ | 295 ± 30^c^ | 674 ± 39^a^ | 266 ± 30^c^ | 382 ± 4^bc^ | 294 ± 33^c^ | 471 ± 24^b^ | **0.007** | **<0.001** | **0.010** |
| GDH.l.65 | 401 ± 41^cd^ | 544 ± 17^ad^ | 441 ± 31^bcd^ | 618 ± 34^ab^ | 370 ± 17^d^ | 493 ± 56^ad^ | 576 ± 34^ac^ | 650 ± 67^a^ | 0.475 | **<0.001** | 0.135 |
| GDH.b.75 | 493 ± 36^b^ | 492 ± 22^b^ | 488 ± 18^b^ | 596 ± 53^b^ | 495 ± 51^b^ | 1016 ± 152^a^ | 650 ± 71^b^ | 1000 ± 72^a^ | **<0.001** | **0.001** | **0.009** |
| GDH.s.75 | 334 ± 0^ac^ | 461 ± 51^ab^ | 287 ± 8^c^ | 424 ± 30^ac^ | 342 ± 60^ac^ | 300 ± 30^bc^ | 355 ± 36^ac^ | 483 ± 19^a^ | 0.793 | **0.007** | **0.015** |
| GDH.p.75 | 325 ± 32 | 334 ± 6 | 313 ± 4 | 395 ± 15 | 408 ± 19 | 374 ± 93 | 328 ± 14 | 445 ± 23 | 0.093 | 0.096 | 0.836 |
| GDH.a.75 | 303 ± 23^bc^ | 282 ± 44^bc^ | 439 ± 26^bc^ | 928 ± 21^a^ | 254 ± 60^c^ | 492 ± 62^b^ | 417 ± 47^bc^ | 810 ± 70^a^ | 0.881 | **<0.001** | **0.018** |
| GDH.g.75 | 186 ± 34^b^ | 562 ± 11^a^ | 477 ± 5^a^ | 532 ± 53^a^ | 193 ± 13^b^ | 503 ± 48^a^ | 437 ± 33^a^ | 469 ± 43^a^ | 0.129 | **<0.001** | 0.727 |
| GDH.l.75 | 407 ± 69^e^ | 694 ± 10^cd^ | 595 ± 34^ce^ | 969 ± 53^a^ | 472 ± 58^de^ | 740 ± 39^bc^ | 647 ± 50^cd^ | 933 ± 32^ab^ | 0.350 | **<0.001** | 0.697 |
| C.b.65 | 459 ± 7 | 454 ± 6 | 454 ± 4 | 439 ± 3 | 441 ± 4 | 451 ± 9 | 457 ± 2 | 438 ± 4 | 0.245 | **0.032** | 0.302 |
| C.s.65 | 457 ± 3^a^ | 448 ± 3^ac^ | 451 ± 3^ac^ | 441 ± 1^bc^ | 448 ± 2^ac^ | 452 ± 4^ab^ | 449 ± 2^ac^ | 439 ± 0^c^ | 0.265 | **<0.001** | 0.123 |
| C.p.65 | 462 ± 1^a^ | 461 ± 1^a^ | 456 ± 3^a^ | 443 ± 2^b^ | 453 ± 2^a^ | 461 ± 1^a^ | 460 ± 3^a^ | 442 ± 1^b^ | 0.423 | **<0.001** | **0.044** |
| C.a.65 | 446 ± 3 | 449 ± 3 | 434 ± 10 | 428 ± 0 | 430 ± 4 | 446 ± 7 | 437 ± 7 | 434 ± 7 | 0.555 | 0.072 | 0.288 |
| C.g.65 | 461 ± 2 | 456 ± 5 | 457 ± 4 | 453 ± 1 | 457 ± 1 | 456 ± 5 | 455 ± 2 | 449 ± 2 | 0.249 | 0.161 | 0.849 |
| C.l.65 | 430 ± 4^ab^ | 444 ± 1^a^ | 426 ± 8^ab^ | 419 ± 3^b^ | 421 ± 3^b^ | 447 ± 9^a^ | 417 ± 3^b^ | 413 ± 2^b^ | 0.144 | **<0.001** | 0.554 |
| C.b.75 | 432 ± 10 | 449 ± 7 | 445 ± 7 | 437 ± 8 | 424 ± 9 | 418 ± 12 | 424 ± 6 | 426 ± 7 | **0.009** | 0.876 | 0.550 |
| C.s.75 | 453 ± 2^a^ | 451 ± 2^ab^ | 449 ± 1^ab^ | 454 ± 4^a^ | 444 ± 1^ab^ | 438 ± 5^b^ | 443 ± 3^ab^ | 446 ± 1^ab^ | **<0.001** | 0.226 | 0.712 |
| C.p.75 | 460 ± 3 | 454 ± 3 | 457 ± 1 | 455 ± 0 | 457 ± 2 | 451 ± 1 | 455 ± 3 | 459 ± 3 | 0.572 | 0.151 | 0.548 |
| C.a.75 | 407 ± 4^ab^ | 415 ± 5^a^ | 401 ± 3^ab^ | 395 ± 9^ab^ | 402 ± 11^ab^ | 401 ± 10^ab^ | 385 ± 3^ab^ | 381 ± 2^b^ | **0.021** | **0.027** | 0.835 |
| C.g.75 | 438 ± 2^ab^ | 444 ± 6^a^ | 430 ± 6^ab^ | 444 ± 3^a^ | 432 ± 8^ab^ | 424 ± 13^ab^ | 411 ± 1^b^ | 438 ± 3^ab^ | **0.009** | **0.034** | 0.552 |
| C.l.75 | 388 ± 4^ab^ | 416 ± 5^a^ | 392 ± 2^ab^ | 392 ± 5^ab^ | 381 ± 11^ab^ | 388 ± 15^ab^ | 371 ± 3^b^ | 382 ± 5^ab^ | **0.006** | 0.063 | 0.495 |
| N.b.65 | 46.1 ± 4.6 | 40.2 ± 5.1 | 32.0 ± 7.8 | 41.8 ± 3.6 | 33.9 ± 5.5 | 40.0 ± 5.0 | 42.8 ± 3.9 | 36.8 ± 1.7 | 0.644 | 0.944 | 0.166 |
| N.s.65 | 15.0 ± 2.7 | 11.7 ± 1.0 | 13.6 ± 2.3 | 12.1 ± 1.7 | 10.8 ± 2.1 | 13.6 ± 2.1 | 13.0 ± 2.1 | 11.1 ± 1.0 | 0.497 | 0.854 | 0.505 |
| N.p.65 | 15.0 ± 1.9 | 15.8 ± 1.6 | 14.2 ± 1.2 | 14.4 ± 0.5 | 12.2 ± 2.4 | 16.0 ± 2.1 | 15.5 ± 1.5 | 10.4 ± 0.4 | 0.282 | 0.192 | 0.345 |
| N.a.65 | 20.0 ± 1.2 | 19.4 ± 1.7 | 20.3 ± 3.0 | 20.5 ± 0.7 | 16.3 ± 1.5 | 18.7 ± 2.0 | 18.1 ± 1.5 | 18.9 ± 0.5 | 0.105 | 0.820 | 0.846 |
| N.g.65 | 13.8 ± 0.9 | 13.5 ± 1.2 | 14.5 ± 1.5 | 13.3 ± 0.7 | 11.7 ± 0.9 | 13.3 ± 1.2 | 13.5 ± 1.2 | 12.3 ± 0.2 | 0.166 | 0.599 | 0.828 |
| N.l.65 | 14.0 ± 1.2 | 13.7 ± 1.1 | 14.1 ± 1.4 | 13.5 ± 0.4 | 12.3 ± 0.8 | 14.0 ± 1.2 | 12.9 ± 0.9 | 12.6 ± 0.4 | 0.255 | 0.857 | 0.780 |

**Supplementary Table 2.** Continued.

| **Trait** | **Control** | | | | **Low N** | | | | ***P*-value** | | |
| --- | --- | --- | --- | --- | --- | --- | --- | --- | --- | --- | --- |
|  | **KNI** | **DRI** | **EUR** | **HAR** | **KNI** | **DRI** | **EUR** | **HAR** | **N** | **G** | **G×N** |
| N.b.75 | 27.5 ± 4.1 | 32.8 ± 0.8 | 32.3 ± 4.9 | 35.8 ± 1.7 | 25.2 ± 2.9 | 28.3 ± 4.5 | 26.3 ± 3.0 | 30.0 ± 1.9 | 0.060 | 0.279 | 0.935 |
| N.s.75 | 9.4 ± 1.1 | 10.3 ± 0.5 | 10.4 ± 1.7 | 11.4 ± 0.3 | 9.6 ± 1.2 | 9.1 ± 1.3 | 7.8 ± 1.0 | 11.0 ± 1.2 | 0.235 | 0.298 | 0.642 |
| N.p.75 | 7.9 ± 0.9 | 9.9 ± 0.6 | 8.0 ± 0.7 | 8.1 ± 0.2 | 9.3 ± 1.6 | 8.8 ± 1.3 | 7.1 ± 0.8 | 7.0 ± 0.4 | 0.489 | 0.178 | 0.495 |
| N.a.75 | 10.9 ± 1.0^ac^ | 13.2 ± 1.7^ab^ | 9.7 ± 0.8^bc^ | 16.0 ± 1.1^a^ | 11.3 ± 1.5^ac^ | 11.9 ± 0.9^ac^ | 7.8 ± 0.4^c^ | 13.2 ± 0.7^ab^ | 0.098 | **0.001** | 0.520 |
| N.g.75 | 10.5 ± 1.8^ab^ | 11.7 ± 1.1^ab^ | 10.3 ± 1.3^ab^ | 14.1 ± 0.7^a^ | 10.4 ± 1.7^ab^ | 10.0 ± 0.7^ab^ | 7.7 ± 0.1^b^ | 11.6 ± 0.9^ab^ | 0.052 | **0.034** | 0.680 |
| N.l.75 | 9.05 ± 1.1^ab^ | 10.3 ± 0.7^ab^ | 7.7 ± 0.7^bc^ | 11.6 ± 0.8^a^ | 7.3 ± 0.7^bc^ | 8.4 ± 0.3^ac^ | 5.6 ± 0.1^c^ | 10.3 ± 0.8^ab^ | **0.002** | **<0.001** | 0.967 |
| CN.b.65 | 10.1 ± 0.8 | 11.7 ± 1.5 | 11.4 ± 0.4 | 10.6 ± 0.8 | 13.6 ± 1.8 | 11.6 ± 1.4 | 10.9 ± 1.0 | 11.9 ± 0.5 | 0.210 | 0.909 | 0.305 |
| CN.s.65 | 32.7 ± 6.2 | 38.9 ± 3.7 | 35.1 ± 5.6 | 38.0 ± 6.0 | 44.4 ± 7.6 | 35.0 ± 5.9 | 36.1 ± 4.8 | 39.6 ± 3.3 | 0.516 | 0.931 | 0.565 |
| CN.p.65 | 32.0 ± 4.5 | 29.9 ± 3.2 | 32.7 ± 2.8 | 30.9 ± 1.1 | 40.1 ± 7.6 | 29.9 ± 4.4 | 30.1 ± 2.4 | 42.5 ± 1.6 | 0.144 | 0.261 | 0.272 |
| CN.a.65 | 22.4 ± 1.2 | 23.5 ± 2.1 | 22.2 ± 2.5 | 20.9 ± 0.7 | 26.7 ± 2.0 | 24.4 ± 2.6 | 24.4 ± 1.6 | 22.9 ± 0.3 | 0.078 | 0.499 | 0.824 |
| CN.g.65 | 33.6 ± 1.8 | 34.3 ± 3.5 | 32.0 ± 2.9 | 34.3 ± 1.7 | 39.6 ± 2.8 | 34.3 ± 3.6 | 34.1 ± 2.7 | 36.4 ± 0.3 | 0.185 | 0.588 | 0.706 |
| CN.l.65 | 31.2 ± 2.3 | 33.4 ± 2.0 | 30.8 ± 2.6 | 31.0 ± 0.9 | 34.3 ± 2.0 | 32.2 ± 2.1 | 32.6 ± 1.9 | 32.8 ± 1.0 | 0.323 | 0.912 | 0.719 |
| CN.b.75 | 16.3 ± 2.0 | 13.7 ± 0.3 | 14.3 ± 1.9 | 12.2 ± 0.4 | 17.2 ± 1.9 | 15.4 ± 2.3 | 16.5 ± 1.7 | 14.3 ± 0.8 | 0.139 | 0.202 | 0.978 |
| CN.s.75 | 49.9 ± 6.2 | 43.9 ± 2.1 | 46.2 ± 8.8 | 39.8 ± 1.0 | 47.8 ± 5.9 | 49.9 ± 6.6 | 58.7 ± 7.5 | 41.8 ± 5.1 | 0.293 | 0.294 | 0.660 |
| CN.p.75 | 59.3 ± 6.0 | 46.1 ± 3.4 | 57.6 ± 4.6 | 55.2 ± 2.0 | 51.9 ± 7.4 | 53.9 ± 9.1 | 65.4 ± 6.6 | 66.1 ± 3.5 | 0.261 | 0.210 | 0.414 |
| CN.a.75 | 37.9 ± 3.1^ac^ | 32.6 ± 4.2^bc^ | 42.0 ± 2.9^ab^ | 24.9 ± 1.3^c^ | 36.6 ± 4.2^ac^ | 33.9 ± 1.8^bc^ | 49.6 ± 2.6^a^ | 29.1 ± 1.6^bc^ | 0.171 | **<0.001** | 0.471 |
| CN.g.75 | 44 ± 7.9 | 38.6 ± 3.1 | 42.8 ± 4.5 | 31.7 ± 1.4 | 44.0 ± 8.0 | 42.7 ± 2.1 | 53.7 ± 0.4 | 38.1 ± 2.7 | 0.125 | 0.061 | 0.673 |
| CN.l.75 | 44.2 ± 5.4^bc^ | 40.8 ± 2.3^bc^ | 51.9 ± 3.9^ab^ | 34.0 ± 2.0^c^ | 53.3 ± 5.4^ab^ | 46.2± 0.1^bc^ | 66.1 ± 0.5^a^ | 37.5 ± 2.3^bc^ | **0.004** | **<0.001** | 0.424 |
| K.b.65 | 17.0 ± 1.0^ab^ | 13.7 ± 1.5^ab^ | 18.1 ± 1.2^a^ | 14.7 ± 1.9^ab^ | 12.8 ± 1.3^ab^ | 10.1 ± 1.4^b^ | 17.7 ± 0.9^a^ | 16.0 ± 2.2^ab^ | 0.126 | **0.009** | 0.251 |
| K.s.65 | 13.1 ± 1.5 | 9.2 ± 1.0 | 13.4 ± 1.5 | 12.6 ± 1.0 | 10.6 ± 0.4 | 8.7 ± 0.2 | 11.2 ± 0.9 | 9.4 ± 1.0 | **0.010** | **0.023** | 0.598 |
| K.p.65 | 13.4 ± 0.6^bc^ | 13.1 ± 0.8^bc^ | 13.3 ± 1.4^bc^ | 17.9 ± 0.8^a^ | 10.3 ± 0.9^c^ | 11.4 ± 1.1^bc^ | 11.5 ± 0.7^bc^ | 14.7 ± 0.5^ab^ | **0.001** | **<0.001** | 0.742 |
| K.a.65 | 8.26 ± 0.62^ab^ | 8.40 ± 1.16^ab^ | 5.37 ± 0.72^ac^ | 6.70 ± 1.07^ac^ | 6.93 ± 0.54^ac^ | 9.02 ± 1.02^a^ | 3.79 ± 0.56^c^ | 4.60 ± 0.42^bc^ | 0.073 | **<0.001** | 0.384 |
| K.g.65 | 7.80 ± 0.29 | 5.70 ± 1.06 | 7.71 ± 0.30 | 5.63 ± 0.36 | 6.83 ± 0.53 | 7.33 ± 0.19 | 6.11 ± 0.07 | 5.68 ± 0.15 | 0.510 | **0.018** | **0.018** |
| K.l.65 | 6.17 ± 0.35 | 5.92 ± 0.06 | 7.04 ± 1.05 | 7.06 ± 0.64 | 5.92 ± 0.35 | 5.92 ± 0.21 | 6.51 ± 0.48 | 6.41 ± 0.10 | 0.329 | 0.232 | 0.922 |
| K.b.75 | 15.9 ± 2.0 | 12.9 ± 0.8 | 18.0 ± 3.0 | 13.8 ± 1.6 | 13.1 ± 1.3 | 12.6 ± 0.8 | 15.9 ± 0.4 | 12.1 ± 1.2 | 0.151 | 0.063 | 0.880 |
| K.s.75 | 10.9 ± 1.4^ab^ | 11.7 ± 2.2^ab^ | 14.4 ± 1.9^a^ | 9.3 ± 0.5^ab^ | 10.6 ± 0.4^ab^ | 10.7 ± 1.0^ab^ | 11.5 ± 0.8^ab^ | 7.2 ± 0.6^b^ | 0.100 | **0.014** | 0.742 |
| K.p.75 | 5.45 ± 0.63^b^ | 9.33 ± 0.81^a^ | 8.35 ± 0.76^ab^ | 8.31 ± 0.54^ab^ | 6.18 ± 0.61^ab^ | 9.10 ± 0.67^a^ | 7.46 ± 0.96^ab^ | 4.86 ± 0.75^b^ | 0.081 | **0.001** | 0.061 |
| K.a.75 | 4.99 ± 0.72 | 5.40 ± 0.93 | 5.47 ± 0.27 | 8.32 ± 0.90 | 6.93 ± 1.39 | 7.16 ± 0.28 | 5.61 ± 0.76 | 7.47 ± 0.95 | 0.229 | 0.064 | 0.327 |
| K.g.75 | 12.3 ± 0.3 | 9.4 ± 1.3 | 13.5 ± 0.2 | 13.0 ± 0.9 | 11.6 ± 0.7 | 12.0 ± 1.4 | 13.6 ± 1.2 | 11.1 ± 0.3 | 0.930 | **0.044** | 0.111 |
| K.l.75 | 7.93 ± 0.39 | 6.85 ± 0.72 | 7.33 ± 0.36 | 8.72 ± 0.03 | 7.99 ± 0.38 | 7.89 ± 1.33 | 7.32 ± 0.69 | 7.18 ± 0.29 | 0.809 | 0.625 | 0.282 |
| Ca.b.65 | 5.97 ± 0.15 | 4.43 ± 0.56 | 3.82 ± 0.26 | 5.29 ± 0.41 | 4.62 ± 0.65 | 5.59 ± 1.10 | 4.80 ± 0.82 | 6.05 ± 0.30 | 0.386 | 0.193 | 0.183 |
| Ca.s.65 | 2.11 ± 0.31 | 1.31 ± 0.18 | 1.29 ± 0.16 | 2.62 ± 0.55 | 1.53 ± 0.18 | 1.96 ± 0.26 | 1.82 ± 0.30 | 2.17 ± 0.16 | 0.856 | **0.043** | 0.100 |
| Ca.p.65 | 0.79 ± 0.13^ab^ | 0.65 ± 0.03^b^ | 0.72 ± 0.06^b^ | 1.08 ± 0.17^ab^ | 0.86 ± 0.10^ab^ | 0.76 ± 0.10^b^ | 0.89 ± 0.08^ab^ | 1.27 ± 0.08^a^ | 0.077 | **0.001** | 0.925 |
| Ca.a.65 | 2.53 ± 0.22^ac^ | 1.74 ± 0.35^c^ | 2.13 ± 0.17^ac^ | 3.35 ± 0.37^ab^ | 2.12 ± 0.32^ac^ | 1.98 ± 0.39^bc^ | 2.07 ± 0.32^bc^ | 3.58 ± 0.20^a^ | 0.996 | **<0.001** | 0.679 |
| Ca.g.65 | 1.21 ± 0.16 | 0.93 ± 0.12 | 0.99 ± 0.06 | 0.96 ± 0.11 | 1.02 ± 0.14 | 0.89 ± 0.18 | 1.03 ± 0.18 | 1.44 ± 0.16 | 0.485 | 0.239 | 0.158 |
| Ca.l.65 | 1.81 ± 0.36^ac^ | 0.68 ± 0.08^c^ | 1.24 ± 0.20^bc^ | 2.12 ± 0.36^ab^ | 1.19 ± 0.15^bc^ | 1.15 ± 0.10^bc^ | 1.32 ± 0.21^bc^ | 2.71 ± 0.40^a^ | 0.495 | **<0.001** | 0.125 |
| Ca.b.75 | 10.0 ± 1.5 | 8.1 ± 0.6 | 6.8 ± 0.4 | 9.0 ± 0.6 | 11.0 ± 2.3 | 11.3 ± 0.2 | 7.8 ± 0.3 | 11.7 ± 0.3 | **0.017** | **0.024** | 0.613 |
| Ca.s.75 | 2.65 ± 0.01^ab^ | 1.95 ± 0.12^b^ | 2.22 ± 0.25^ab^ | 3.05 ± 0.12^ab^ | 2.32 ± 0.46^ab^ | 2.41 ± 0.05^ab^ | 1.86 ± 0.11^b^ | 3.19 ± 0.40^a^ | 0.902 | **0.002** | 0.309 |
| Ca.p.75 | 1.46 ± 0.11^ac^ | 1.28 ± 0.21^ac^ | 0.94 ± 0.07^c^ | 1.05 ± 0.11^bc^ | 1.69 ± 0.26^ab^ | 1.83 ± 0.16^a^ | 1.12 ± 0.12^ac^ | 1.27 ± 0.06^ac^ | **0.015** | **0.004** | 0.597 |
| Ca.a.75 | 2.27 ± 0.17^bc^ | 1.74 ± 0.18^c^ | 2.15 ± 0.16^bc^ | 3.37 ± 0.34^ab^ | 2.51 ± 0.53^ac^ | 2.58 ± 0.37^ac^ | 2.15 ± 0.13^bc^ | 3.84 ± 0.35^a^ | 0.091 | **<0.001** | 0.573 |
| Ca.g.75 | 0.84 ± 0.12^ab^ | 0.54 ± 0.05^b^ | 0.77 ± 0.06^ab^ | 0.99 ± 0.10^ab^ | 0.94 ± 0.19^ab^ | 0.81 ± 0.14^ab^ | 0.65 ± 0.02^b^ | 1.24 ± 0.08^a^ | 0.123 | **0.003** | 0.278 |
| Ca.l.75 | 0.95 ± 0.07^ac^ | 0.54 ± 0.07^c^ | 0.68 ± 0.10^ac^ | 1.03 ± 0.04^ab^ | 1.04 ± 0.18^ab^ | 0.79 ± 0.14^ac^ | 0.57 ± 0.06^bc^ | 1.12 ± 0.03^a^ | 0.261 | **<0.001** | 0.377 |

**Supplementary Table 2.** Continued.

| **Trait** | **Control** | | | | **Low N** | | | | ***P*-value** | | |
| --- | --- | --- | --- | --- | --- | --- | --- | --- | --- | --- | --- |
|  | **KNI** | **DRI** | **EUR** | **HAR** | **KNI** | **DRI** | **EUR** | **HAR** | **N** | **G** | **G×N** |
| P.b.65 | 2.60 ± 0.22 | 2.76 ± 0.33 | 3.99 ± 0.67 | 2.56 ± 0.30 | 2.61 ± 0.10 | 2.58 ± 0.18 | 4.03 ± 0.23 | 2.61 ± 0.39 | 0.936 | **0.002** | 0.986 |
| P.s.65 | 1.09 ± 0.07^bc^ | 0.96 ± 0.11^c^ | 1.61 ± 0.04^a^ | 1.16 ± 0.06^bc^ | 1.27 ± 0.03^ac^ | 1.07 ± 0.05^bc^ | 1.40 ± 0.08^ab^ | 1.39 ± 0.15^ab^ | 0.174 | **<0.001** | 0.066 |
| P.p.65 | 2.79 ± 0.10^a^ | 3.43 ± 0.15^a^ | 2.81 ± 0.29^a^ | 3.18 ± 0.20^a^ | 1.93 ± 0.09^b^ | 3.12 ± 0.06^a^ | 3.00 ± 0.18^a^ | 2.83 ± 0.20^a^ | **0.016** | **0.001** | 0.057 |
| P.a.65 | 1.77 ± 0.23^ab^ | 2.11 ± 0.35^a^ | 1.82 ± 0.13^ab^ | 1.41 ± 0.17^ab^ | 1.67 ± 0.12^ab^ | 1.94 ± 0.17^ab^ | 1.16 ± 0.10^b^ | 1.36 ± 0.13^ab^ | 0.091 | **0.019** | 0.380 |
| P.g.65 | 2.00 ± 0.31 | 1.85 ± 0.30 | 2.34 ± 0.12 | 1.60 ± 0.17 | 1.97 ± 0.07 | 2.35 ± 0.01 | 1.63 ± 0.07 | 1.66 ± 0.12 | 0.716 | 0.082 | **0.027** |
| P.l.65 | 1.82 ± 0.15 | 1.92 ± 0.05 | 2.17 ± 0.28 | 1.94 ± 0.23 | 1.70 ± 0.13 | 1.82 ± 0.06 | 1.59 ± 0.09 | 2.58 ± 0.54 | 0.821 | 0.224 | 0.135 |
| P.b.75 | 1.62 ± 0.10^b^ | 2.00 ± 0.36^b^ | 2.80 ± 0.21^ab^ | 2.14 ± 0.27^b^ | 2.04 ± 0.30^b^ | 1.98 ± 0.05^b^ | 3.78 ± 0.34^a^ | 1.77 ± 0.14^b^ | 0.168 | **<0.001** | 0.076 |
| P.s.75 | 0.71 ± 0.05^c^ | 0.72 ± 0.09^c^ | 1.05 ± 0.05^ac^ | 1.28 ± 0.22^ab^ | 1.33 ± 0.06^ab^ | 0.80 ± 0.11^bc^ | 1.55 ± 0.11^a^ | 0.84 ± 0.07^bc^ | **0.023** | **0.002** | **0.001** |
| P.p.75 | 0.68 ± 0.09^c^ | 1.68 ± 0.14^ab^ | 1.52 ± 0.26^ab^ | 1.54 ± 0.20^ab^ | 0.87 ± 0.14^bc^ | 1.28 ± 0.23^ac^ | 1.87 ± 0.10^a^ | 1.11 ± 0.15^ac^ | 0.575 | **<0.001** | 0.081 |
| P.a.75 | 0.71 ± 0.04^c^ | 1.05 ± 0.23^ac^ | 0.80 ± 0.04^bc^ | 1.39 ± 0.05^ab^ | 1.48 ± 0.21^a^ | 0.97 ± 0.13^ac^ | 0.89 ± 0.09^ac^ | 1.00 ± 0.08^ac^ | 0.318 | 0.093 | **0.003** |
| P.g.75 | 0.97 ± 0.02 | 1.33 ± 0.27 | 1.24 ± 0.07 | 1.52 ± 0.14 | 1.40 ± 0.22 | 1.01 ± 0.15 | 1.18 ± 0.04 | 1.26 ± 0.06 | 0.632 | 0.435 | 0.080 |
| P.l.75 | 0.94 ± 0.05^b^ | 1.07 ± 0.15^b^ | 0.91 ± 0.05^b^ | 2.30 ± 0.43^a^ | 1.06 ± 0.06^b^ | 1.08 ± 0.13^b^ | 0.80 ± 0.06^b^ | 1.06 ± 0.10^b^ | **0.027** | **0.001** | **0.005** |
| Mg.b.65 | 1.48 ± 0.20^ad^ | 1.65 ± 0.08^ac^ | 1.26 ± 0.11^cd^ | 1.92 ± 0.11^a^ | 1.30 ± 0.08^bcd^ | 1.69 ± 0.08^ac^ | 1.08 ± 0.02^d^ | 1.77 ± 0.04^ab^ | 0.114 | **<0.001** | 0.660 |
| Mg.s.65 | 0.510 ± 0.041^b^ | 0.476 ± 0.029^b^ | 0.534 ± 0.027^ab^ | 0.730 ± 0.077^a^ | 0.405 ± 0.024^b^ | 0.598 ± 0.056^ab^ | 0.526 ± 0.051^ab^ | 0.603 ± 0.016^ab^ | 0.365 | **0.002** | 0.052 |
| Mg.p.65 | 0.739 ± 0.011^a^ | 0.732 ± 0.025^a^ | 0.652 ± 0.020^ab^ | 0.737 ± 0.019^a^ | 0.686 ± 0.024^ab^ | 0.763 ± 0.055^a^ | 0.647 ± 0.027^ab^ | 0.565 ± 0.012^b^ | **0.019** | **0.005** | **0.010** |
| Mg.a.65 | 1.12 ± 0.06^ab^ | 1.17 ± 0.03^ab^ | 0.86 ± 0.09^bc^ | 1.36 ± 0.04^a^ | 1.09 ± 0.03^ac^ | 1.21 ± 0.15^ab^ | 0.74 ± 0.06^c^ | 1.31 ± 0.05^a^ | 0.470 | **<0.001** | 0.784 |
| Mg.g.65 | 0.900 ± 0.067^a^ | 0.836 ± 0.028^ac^ | 0.663 ± 0.065^bc^ | 0.830 ± 0.024^ac^ | 0.906 ± 0.019^a^ | 0.854 ± 0.045^ab^ | 0.644 ± 0.019^c^ | 0.821 ± 0.020^ac^ | 0.976 | **<0.001** | 0.971 |
| Mg.l.65 | 0.997 ± 0.073^bc^ | 0.804 ± 0.031^cd^ | 0.606 ± 0.027^de^ | 1.249 ± 0.019^ab^ | 0.923 ± 0.032^c^ | 0.864 ± 0.042^cd^ | 0.524 ± 0.040^e^ | 1.267± 0.106^a^ | 0.618 | **<0.001** | 0.486 |
| Mg.b.75 | 1.98 ± 0.14^ab^ | 1.99 ± 0.07^ab^ | 1.31 ± 0.05^c^ | 2.09 ± 0.13^ab^ | 1.90 ± 0.07^ac^ | 1.85 ± 0.11^ac^ | 1.49 ± 0.14^bc^ | 2.13 ± 0.21^a^ | 0.995 | **<0.001** | 0.601 |
| Mg.s.75 | 0.779 ± 0.069^ab^ | 0.409 ± 0.016^b^ | 0.692 ± 0.029^ab^ | 0.954 ± 0.044^a^ | 0.780 ± 0.056^ab^ | 0.717 ± 0.105^ab^ | 0.728 ± 0.060^ab^ | 1.020 ± 0.147^a^ | 0.076 | **<0.001** | 0.215 |
| Mg.p.75 | 0.341 ± 0.052 | 0.402 ± 0.010 | 0.389 ± 0.044 | 0.327 ± 0.014 | 0.314 ± 0.059 | 0.366 ± 0.062 | 0.301 ± 0.020 | 0.256 ± 0.034 | 0.078 | 0.205 | 0.873 |
| Mg.a.75 | 1.06 ± 0.11^ac^ | 1.06 ± 0.03^ac^ | 0.83 ± 0.05^c^ | 1.40 ± 0.12^a^ | 1.15 ± 0.03^ac^ | 1.26 ± 0.13^ab^ | 0.93 ± 0.09^bc^ | 1.30 ± 0.02^ab^ | 0.224 | **<0.001** | 0.337 |
| Mg.g.75 | 0.532 ± 0.049^ab^ | 0.498 ± 0.033^ab^ | 0.398 ± 0.040^b^ | 0.651 ± 0.0489^a^ | 0.639 ± 0.068^a^ | 0.526 ± 0.027^ab^ | 0.409 ± 0.034^b^ | 0.682 ± 0.028^a^ | 0.164 | **<0.001** | 0.689 |
| Mg.l.75 | 0.556 ± 0.021^a^ | 0.444 ± 0.038^ab^ | 0.278 ± 0.028^c^ | 0.480 ± 0.034^a^ | 0.301 ± 0.046^bc^ | 0.488 ± 0.023^a^ | 0.259 ± 0.007^c^ | 0.479 ± 0.047^a^ | **0.025** | **<0.001** | **0.001** |
| Fe.b.65 | 159 ± 8 | 113 ± 19 | 124 ± 12 | 160 ± 33 | 118 ± 10 | 119 ± 8 | 152 ± 8 | 153 ± 10 | 0.752 | 0.126 | 0.214 |
| Fe.s.65 | 46.1 ± 10.4 | 43.6 ± 6.6 | 52.8 ± 6.0 | 82.4 ± 16.4 | 33.0 ± 5.4 | 57.2 ± 6.9 | 69.9 ± 15.3 | 76.2 ± 11.5 | 0.710 | **0.011** | 0.428 |
| Fe.p.65 | 21.6 ± 3.3^c^ | 28.1 ± 3.7^bc^ | 29.7 ± 1.0^bc^ | 53.0 ± 7.3^a^ | 30.4 ± 4.8^bc^ | 29.5 ± 3.7^bc^ | 37.6 ± 4.7^ac^ | 44.4 ± 5.7^ab^ | 0.474 | **0.001** | 0.245 |
| Fe.a.65 | 44.9 ± 2.3^b^ | 55.8 ± 8.6^ab^ | 64.2 ± 12.7^ab^ | 110.0 ± 23.9^ab^ | 49.1 ± 5.1^b^ | 66.4 ± 6.0^ab^ | 109.1 ± 2.1^ab^ | 125.0 ± 28.1^a^ | 0.086 | **0.001** | 0.524 |
| Fe.g.65 | 76.4 ± 9.9^c^ | 72.3 ± 3.9^c^ | 344.9 ± 15.9^b^ | 266.5 ± 23.2^bc^ | 81.4 ± 15.3^c^ | 87.9 ± 15.3^c^ | 707.2 ± 128.5^a^ | 171.3 ± 11.6^bc^ | **0.048** | **<0.001** | **0.001** |
| Fe.l.65 | 51.7 ± 8.2^c^ | 81.8 ± 9.6^bc^ | 166.0 ± 31.7^ac^ | 196.7 ± 24.0^ab^ | 60.7 ± 7.4^c^ | 56.2 ± 6.3^c^ | 253.1 ± 47.0^a^ | 173.1 ± 33.3^ac^ | 0.522 | **<0.001** | 0.134 |
| Fe.b.75 | 88 ± 6 | 133 ± 19 | 119 ± 11 | 113 ± 13 | 143 ± 15 | 128 ± 22 | 146 ± 6 | 133 ± 17 | **0.033** | 0.652 | 0.277 |
| Fe.s.75 | 51.6 ± 2.9 | 62.1 ± 10.6 | 74.8 ± 12.4 | 61.5 ± 5.2 | 52.0 ± 14.4 | 73.2 ± 6.0 | 63.3 ± 5.7 | 75.8 ± 3.3 | 0.566 | 0.173 | 0.453 |
| Fe.p.75 | 22.2 ± 2.4 | 33.7 ± 7.4 | 28.0 ± 8.4 | 20.5 ± 2.0 | 21.1 ± 2.2 | 21.9 ± 4.8 | 24.4 ± 3.5 | 22.6 ± 2.6 | 0.300 | 0.471 | 0.525 |
| Fe.a.75 | 49.4 ± 2.5 | 71.4 ± 7.3 | 50.5 ± 7.3 | 43.7 ± 1.3 | 62.4 ± 10.4 | 65.7 ± 8.3 | 62.9 ± 5.3 | 53.7 ± 2.1 | 0.119 | **0.047** | 0.434 |
| Fe.g.75 | 218 ± 11^bc^ | 124 ± 7^c^ | 425 ± 43^b^ | 126 ± 17^c^ | 262 ± 37^bc^ | 208 ± 18^c^ | 776 ± 99^a^ | 239 ± 39^bc^ | **<0.001** | **<0.001** | **0.012** |
| Fe.l.75 | 117 ± 13^bc^ | 73 ± 2^c^ | 177 ± 9^ab^ | 83 ± 11^c^ | 172 ± 36^ab^ | 93 ± 7^bc^ | 229 ± 13^a^ | 126 ± 22^bc^ | **0.003** | **<0.001** | 0.734 |
| Mn.b.65 | 63.9 ± 5.2^a^ | 35.1 ± 7.0^b^ | 36.0 ± 2.4^b^ | 55.6 ± 4.6^ab^ | 38.1 ± 6.0^ab^ | 45.7 ± 4.2^ab^ | 51.0 ± 9.4^ab^ | 49.9 ± 1.6^ab^ | 0.715 | 0.120 | **0.008** |
| Mn.s.65 | 26.7 ± 2.7^ab^ | 17.9 ± 1.3^b^ | 27.8 ± 5.3^ab^ | 35.2 ± 4.8^a^ | 21.9 ± 3.2^ab^ | 24.5 ± 2.1^ab^ | 26.6 ± 4.3^ab^ | 27.9 ± 1.8^ab^ | 0.497 | **0.049** | 0.247 |
| Mn.p.65 | 41.0 ± 1.4 | 36.8 ± 2.1 | 38.5 ± 5.5 | 40.8 ± 2.8 | 32.8 ± 4.1 | 37.8 ± 4.9 | 35.8 ± 4.0 | 35.2 ± 1.3 | 0.145 | 0.992 | 0.623 |
| Mn.a.65 | 13.4 ± 1.9^b^ | 10.0 ± 2.1^b^ | 16.8 ± 3.4^b^ | 36.0 ± 5.4^a^ | 12.6 ± 1.9^b^ | 13.4 ± 1.0^b^ | 12.5 ± 2.4^b^ | 31.6 ± 0.9^a^ | 0.436 | **<0.001** | 0.459 |
| Mn.g.65 | 12.3 ± 1.1^b^ | 12.0 ± 2.5^b^ | 15.1 ± 2.1^ab^ | 23.4 ± 2.4^a^ | 12.6 ± 2.3^b^ | 13.1 ± 1.0^b^ | 18.2 ± 3.1^ab^ | 20.7 ± 0.5^ab^ | 0.757 | **0.001** | 0.573 |
| Mn.l.65 | 26.9 ± 4.5^ab^ | 10.3 ± 1.7^c^ | 16.3 ± 2.1^bc^ | 36.0 ± 2.9^a^ | 22.0 ± 2.4^bc^ | 17.7 ± 1.4^bc^ | 14.3 ± 2.5^c^ | 35.7 ± 0.7^a^ | 0.975 | **<0.001** | 0.124 |

**Supplementary Table 2.** Continued.

| **Trait** | **Control** | | | | **Low N** | | | | ***P*-value** | | |
| --- | --- | --- | --- | --- | --- | --- | --- | --- | --- | --- | --- |
|  | **KNI** | **DRI** | **EUR** | **HAR** | **KNI** | **DRI** | **EUR** | **HAR** | **N** | **G** | **G×N** |
| Mn.b.75 | 117.3 ± 12.8^ab^ | 62.3 ± 10.7^b^ | 91.6 ± 9.1^ab^ | 83.5 ± 2.7^ab^ | 142.3 ± 19.8^a^ | 102.3 ± 10.1^ab^ | 113.3 ± 14.9^ab^ | 92.1 ± 9.4^ab^ | **0.013** | **0.006** | 0.646 |
| Mn.s.75 | 37.5 ± 3.2^ab^ | 31.7 ± 4.6^b^ | 39.9 ± 4.6^ab^ | 39.3 ± 2.5^ab^ | 35.9 ± 2.3^ab^ | 39.1 ± 0.6^ab^ | 34.1 ± 4.2^ab^ | 49.5 ± 4.2^a^ | 0.320 | 0.081 | 0.124 |
| Mn.p.75 | 9.9 ± 0.7 | 14.4 ± 2.0 | 10.8 ± 2.2 | 14.0 ± 1.6 | 11.1 ± 1.5 | 11.9 ± 2.1 | 9.7 ± 0.7 | 11.1 ± 0.2 | 0.231 | 0.193 | 0.543 |
| Mn.a.75 | 33.4 ± 3.6^ab^ | 22.1 ± 5.1^b^ | 36.2 ± 4.6^ab^ | 48.2 ± 10.1^ab^ | 23.8 ± 4.1^ab^ | 31.1 ± 5.1^ab^ | 31.5 ± 5.5^ab^ | 51.2 ± 4.8^a^ | 0.883 | **0.004** | 0.403 |
| Mn.g.75 | 16.0 ± 2.0^ab^ | 9.1 ± 1.3^b^ | 20.3 ± 4.6^a^ | 19.1 ± 1.1^ab^ | 12.9 ± 2.0^ab^ | 11.9 ± 0.9^ab^ | 15.1 ± 1.5^ab^ | 20.7 ± 2.5^a^ | 0.551 | **0.004** | 0.282 |
| Mn.l.75 | 26.8 ± 2.7^abc^ | 12.9 ± 1.4^d^ | 19.1 ± 2.9^bd^ | 28.0 ± 2.0^ab^ | 18.0 ± 2.5^bd^ | 20.2 ± 1.9^bd^ | 16.3 ± 0.1^cd^ | 34.5 ± 2.7^a^ | 0.733 | **<0.001** | **0.005** |
| Cu.b.65 | 7.59 ± 0.23 | 6.26 ± 0.65 | 7.73 ± 0.48 | 5.91 ± 0.69 | 6.29 ± 1.19 | 6.66 ± 0.93 | 6.34 ± 0.66 | 5.43 ± 0.80 | 0.212 | 0.288 | 0.616 |
| Cu.s.65 | 3.72 ± 0.53 | 2.31 ± 0.26 | 3.65 ± 0.45 | 2.31 ± 0.30 | 3.73 ± 0.37 | 3.09 ± 0.60 | 2.61 ± 0.43 | 1.72 ± 0.19 | 0.481 | **0.006** | 0.181 |
| Cu.p.65 | 4.71 ± 0.36^ab^ | 4.48 ± 0.15^ab^ | 4.74 ± 0.39^ab^ | 5.30 ± 0.20^a^ | 4.42 ± 0.43^ab^ | 4.52 ± 0.66^ab^ | 3.53 ± 0.41^ab^ | 3.31 ± 0.45^b^ | **0.009** | 0.719 | 0.090 |
| Cu.a.65 | 4.62 ± 0.61 | 3.88 ± 0.33 | 4.52 ± 0.70 | 4.54 ± 0.95 | 4.78 ± 0.55 | 4.63 ± 0.92 | 2.73 ± 0.06 | 3.16 ± 0.39 | 0.225 | 0.368 | 0.179 |
| Cu.g.65 | 4.74 ± 0.59 | 3.63 ± 0.26 | 4.97 ± 0.11 | 2.80 ± 0.22 | 4.67 ± 0.94 | 4.41 ± 0.86 | 3.81 ± 0.84 | 2.33 ± 0.26 | 0.598 | **0.012** | 0.461 |
| Cu.l.65 | 4.83 ± 0.49^a^ | 3.74 ± 0.48^ab^ | 4.36 ± 0.46^ab^ | 2.62 ± 0.48^b^ | 4.51 ± 0.11^ab^ | 4.00 ± 0.56^ab^ | 2.52 ± 0.20^b^ | 2.87 ± 0.45^ab^ | 0.197 | **0.003** | 0.085 |
| Cu.b.75 | 3.97 ± 0.16 | 5.79 ± 0.32 | 3.95 ± 0.48 | 4.95 ± 0.73 | 4.76 ± 0.36 | 4.49 ± 0.68 | 5.16 ± 0.63 | 4.55 ± 0.27 | 0.830 | 0.454 | 0.081 |
| Cu.s.75 | 1.28 ± 0.17^c^ | 2.70 ± 0.11^ab^ | 1.29 ± 0.23^c^ | 1.15 ± 0.26^c^ | 1.43 ± 0.17^c^ | 1.63 ± 0.21^bc^ | 2.96 ± 0.45^a^ | 1.16 ± 0.12^c^ | 0.273 | **0.001** | **<0.001** |
| Cu.p.75 | 1.24 ± 0.25^b^ | 3.05 ± 0.25^a^ | 1.97 ± 0.36^ab^ | 1.54 ± 0.19^b^ | 1.89 ± 0.28^ab^ | 2.11 ± 0.26^ab^ | 2.62 ± 0.44^ab^ | 1.25 ± 0.18^b^ | 0.932 | **0.002** | **0.037** |
| Cu.a.75 | 1.23 ± 0.13^b^ | 3.47 ± 0.06^a^ | 1.12 ± 0.15^b^ | 2.88 ± 0.23^a^ | 2.01 ± 0.34^ab^ | 2.56 ± 0.10^ab^ | 3.10 ± 0.64^a^ | 2.28 ± 0.36^ab^ | 0.171 | **0.002** | **0.001** |
| Cu.g.75 | 2.15 ± 0.42^bc^ | 3.47 ± 0.14^ab^ | 1.29 ± 0.29^c^ | 1.61 ± 0.30^c^ | 2.59 ± 0.32^ac^ | 2.18 ± 0.11^bc^ | 4.32 ± 0.70^a^ | 1.93 ± 0.30^bc^ | **0.027** | **0.035** | **<0.001** |
| Cu.l.75 | 0.24 ± 0.01^c^ | 3.30 ± 0.30^a^ | 1.35 ± 0.28^bc^ | 2.28 ± 0.21^ab^ | 1.86 ± 0.21^b^ | 1.86 ± 0.26^b^ | 2.31 ± 0.16^ab^ | 2.05 ± 0.25^b^ | 0.177 | **<0.001** | **<0.001** |
| C.grain.75 | 441 ± 2 | 439 ± 3 | 435 ± 2 | 438 ± 4 | 437 ± 2 | 435 ± 0 | 434 ± 3 | 437 ± 0 | 0.156 | 0.334 | 0.854 |
| C.grain.85 | 436 ± 2 | 435 ± 1 | 435 ± 3 | 438 ± 0 | 434 ± 1 | 432 ± 0 | 433 ± 1 | 436 ± 0 | 0.063 | 0.122 | 0.940 |
| C.grain.92 | 435 ± 2 | 437 ± 0 | 436 ± 5 | 439 ± 1 | 435 ± 2 | 434 ± 0 | 433 ± 2 | 437 ± 0 | 0.185 | 0.390 | 0.784 |
| N.grain.75 | 24.9 ± 1.4^ab^ | 25.0 ± 0.4^ab^ | 23.8 ± 0.5^ac^ | 20.4 ± 1.2^bc^ | 23.2 ± 1.7^ac^ | 25.7 ± 0.7^a^ | 21.3 ± 0.2^ac^ | 19.7 ± 0.6^c^ | 0.163 | **<0.001** | 0.425 |
| N.grain.85 | 27.1 ± 2.8 | 24.2 ± 1.2 | 24.1 ± 3.6 | 21.3 ± 2.4 | 26.7 ± 2.9 | 24.0 ± 1.6 | 21.9 ± 3.5 | 21.0 ± 1.5 | 0.695 | 0.209 | 0.974 |
| N.grain.92 | 26.1 ± 2.7 | 23.7 ± 0.6 | 26.3 ± 1.9 | 22.4 ± 1.0 | 26.0 ± 2.8 | 23.3 ± 1.5 | 19.0 ± 1.2 | 20.9 ± 1.2 | 0.080 | 0.113 | 0.189 |
| CN.grain.75 | 17.0 ± 1.3^b^ | 17.6 ± 0.4^ab^ | 18.3 ± 0.5^ab^ | 21.6 ± 1.2^a^ | 19.0 ± 1.3^ab^ | 16.7 ± 0.5^b^ | 20.4 ± 0.3^ab^ | 21.3 ± 0.2^a^ | 0.247 | **0.001** | 0.202 |
| CN.grain.85 | 16.5 ± 1.9 | 17.9 ± 1.1 | 18.9 ± 2.9 | 20.3 ± 1.9 | 16.7 ± 1.9 | 18.0 ± 1.1 | 20.7 ± 2.8 | 21.0 ± 1.5 | 0.638 | 0.213 | 0.971 |
| CN.grain.92 | 17.0 ± 1.9 | 18.0 ± 1.0 | 16.7 ± 1.0 | 19.2 ± 0.4 | 17.2 ± 2.0 | 18.4 ± 0.9 | 22.9 ± 1.3 | 21.1 ± 1.3 | **0.033** | 0.108 | 0.119 |
| K.grain.92 | 4.25 ± 0.21 | 4.00 ± 0.07 | 4.36 ± 0.12 | 4.42 ± 0.21 | 4.05 ± 0.14 | 3.86 ± 0.12 | 4.17 ± 0.13 | 4.19 ± 0.25 | 0.133 | 0.153 | 0.994 |
| P.grain.92 | 4.00 ± 0.07 | 4.19 ± 0.05 | 4.15 ± 0.06 | 3.82 ± 0.04 | 3.96 ± 0.08 | 3.98 ± 0.15 | 4.13 ± 0.12 | 3.77 ± 0.13 | 0.250 | **0.012** | 0.729 |
| S.grain.92 | 1.53 ± 0.11 | 1.31 ± 0.04 | 1.39 ± 0.10 | 1.59 ± 0.11 | 1.37 ± 0.07 | 1.25 ± 0.04 | 1.35 ± 0.11 | 1.46 ± 0.02 | 0.108 | 0.050 | 0.900 |
| Mg.grain.92 | 1.21 ± 0.05 | 1.20 ± 0.04 | 1.15 ± 0.03 | 1.13 ± 0.04 | 1.24 ± 0.04 | 1.20 ± 0.03 | 1.17 ± 0.05 | 1.12 ± 0.04 | 0.687 | 0.124 | 0.964 |
| Ca.grain.92 | 0.279 ± 0.007^b^ | 0.229 ± 0.010^c^ | 0.273 ± 0.008^bc^ | 0.375 ± 0.011^a^ | 0.284 ± 0.011^b^ | 0.247 ± 0.008^bc^ | 0.288 ± 0.011^b^ | 0.384 ± 0.009^a^ | 0.104 | **<0.001** | 0.913 |
| Mn.grain.92 | 32.69 ± 2.68 | 32.71 ± 2.13 | 32.47 ± 1.04 | 34.39 ± 1.77 | 30.62 ± 1.39 | 31.51 ± 0.97 | 30.03 ± 2.45 | 31.63 ± 1.60 | 0.125 | 0.802 | 0.977 |
| Fe.grain.92 | 33.60 ± 0.9 | 41.4 ± 4.3 | 35.6 ± 2.5 | 31.1 ± 0.6 | 34.0 ± 2.3 | 38.2 ± 2.5 | 33.0 ± 3.0 | 30.5 ± 2.4 | 0.416 | **0.020** | 0.884 |
| Na.grain.92 | 17.8 ± 4.6 | 15.9 ± 3.1 | 19.9 ± 4.5 | 21.8 ± 3.8 | 15.7 ± 2.1 | 21.9 ± 2.9 | 19.7 ± 0.8 | 29.9 ± 2.3 | 0.215 | 0.070 | 0.369 |
| Zn.grain.92 | 21.0 ± 1.2^ab^ | 18.8 ± 2.2^ab^ | 21.0 ± 1.6^ab^ | 15.6 ± 0.2^b^ | 22.5 ± 2.1^ab^ | 22.9 ± 1.4^ab^ | 24.1 ± 2.2^a^ | 18.1 ± 1.8^ab^ | **0.033** | **0.018** | 0.895 |
| Cu.grain.92 | 5.01 ± 0.47 | 4.49 ± 0.16 | 4.37 ± 0.28 | 4.73 ± 0.30 | 4.26 ± 0.37 | 3.80 ± 0.17 | 3.85 ± 0.21 | 4.20 ± 0.07 | **0.006** | 0.220 | 0.966 |
| Mo.grain.92 | 0.552 ± 0.101 | 0.709 ± 0.074 | 0.567 ± 0.099 | 0.754 ± 0.033 | 0.622 ± 0.049 | 0.493 ± 0.078 | 0.582 ± 0.075 | 0.670 ± 0.024 | 0.304 | 0.242 | 0.243 |
| GCY | 2638 ± 105^ad^ | 2827 ± 337^abc^ | 3110 ± 203^ab^ | 3323 ± 17^a^ | 1842 ± 195^d^ | 2196 ± 121^cd^ | 2337 ± 208^bd^ | 2831 ± 127^abc^ | **<0.001** | **0.003** | 0.836 |
| GNY | 158 ± 17^ab^ | 153 ± 18^ab^ | 188 ± 17^a^ | 169 ± 8^ab^ | 112 ± 22^b^ | 118 ± 11^ab^ | 104 ± 15^b^ | 135 ± 3^ab^ | **<0.001** | 0.629 | 0.340 |
| GKY | 25.8 ± 1.6^ad^ | 25.8 ± 2.9^ad^ | 31.0 ± 1.3^ab^ | 33.4 ± 1.6^a^ | 17.2 ± 2.2^d^ | 19.5 ± 0.8^cd^ | 22.5 ± 2.1^bcd^ | 27.2 ± 2.6^ac^ | **<0.001** | **0.001** | 0.882 |
| GPY | 24.2 ± 0.5^ab^ | 27.1 ± 3.2^a^ | 29.6 ± 2.1^a^ | 29.0 ± 0.3^a^ | 16.8 ± 1.8^b^ | 20.2 ± 1.4^ab^ | 22.4 ± 2.5^ab^ | 24.5 ± 1.9^ab^ | **<0.001** | **0.022** | 0.859 |
| GSY | 9.22 ± 0.52^ab^ | 8.49 ± 1.09^ab^ | 10.01 ± 1.21^ab^ | 12.01 ± 0.86^a^ | 5.80 ± 0.72^b^ | 6.33 ± 0.52^b^ | 7.35 ± 1.21^b^ | 9.47 ± 0.54^ab^ | **0.001** | **0.005** | 0.908 |
| GMgY | 7.31 ± 0.11^ab^ | 7.76 ± 1.00^ab^ | 8.19 ± 0.34^a^ | 8.54 ± 0.31^a^ | 5.27 ± 0.67^b^ | 6.09 ± 0.32^ab^ | 6.38 ± 0.83^ab^ | 7.27 ± 0.53^ab^ | **0.001** | 0.083 | 0.927 |

**Supplementary Table 2.** Continued.

| **Trait** | **Control** | | | | **Low N** | | | | ***P*-value** | | |
| --- | --- | --- | --- | --- | --- | --- | --- | --- | --- | --- | --- |
|  | **KNI** | **DRI** | **EUR** | **HAR** | **KNI** | **DRI** | **EUR** | **HAR** | **N** | **G** | **G×N** |
| GCaY | 1.69 ± 0.10^c^ | 1.49 ± 0.23^c^ | 1.96 ± 0.17^bc^ | 2.84 ± 0.07^a^ | 1.21 ± 0.16^c^ | 1.25 ± 0.09^c^ | 1.56 ± 0.19^c^ | 2.49 ± 0.17^ab^ | **0.004** | **<0.001** | 0.886 |
| GMnY | 0.197 ± 0.011^ac^ | 0.211 ± 0.027^ac^ | 0.232 ± 0.019^ab^ | 0.260 ± 0.012^a^ | 0.130 ± 0.017^c^ | 0.160 ± 0.014^bc^ | 0.164 ± 0.027^bc^ | 0.205 ± 0.017^ac^ | **<0.001** | **0.015** | 0.957 |
| GFeY | 0.204 ± 0.011 | 0.270 ± 0.049 | 0.256 ± 0.032 | 0.236 ± 0.006 | 0.146 ± 0.023 | 0.193 ± 0.011 | 0.181 ± 0.029 | 0.197 ± 0.017 | **0.004** | 0.188 | 0.868 |
| GNaY | 0.108 ± 0.028^ab^ | 0.102 ± 0.0204^ab^ | 0.140 ± 0.025^ab^ | 0.165 ± 0.029^ab^ | 0.068 ± 0.015^b^ | 0.110 ± 0.011^ab^ | 0.107 ± 0.013^ab^ | 0.195 ± 0.023^a^ | 0.573 | **0.004** | 0.351 |
| GZnY | 0.128 ± 0.009 | 0.119 ± 0.010 | 0.149 ± 0.012 | 0.118 ± 0.002 | 0.096 ± 0.015 | 0.115 ± 0.002 | 0.132 ± 0.022 | 0.116 ± 0.007 | 0.119 | 0.104 | 0.569 |
| GCuY | 30.2 ± 2.1^ac^ | 29.0 ± 3.7^ac^ | 31.2 ± 2.9^ab^ | 35.8 ± 2.3^a^ | 18.2 ± 3.1^c^ | 19.2 ± 9.3^bc^ | 21.0 ± 2.8^bc^ | 27.2 ± 1.6^ac^ | **<0.001** | **0.035** | 0.931 |
| GMoY | 3.32 ± 0.52^bc^ | 4.48 ± 0.17^ab^ | 4.04 ± 0.72^ac^ | 5.70 ± 0.22^a^ | 2.66 ± 0.44^bc^ | 2.46 ± 0.29^c^ | 3.10 ± 0.35^bc^ | 4.33 ± 0.12^ac^ | **<0.001** | **0.001** | 0.372 |
| GProtY | 882 ± 90 | 917 ± 115 | 994 ± 148 | 1022 ± 72 | 597 ± 97 | 721 ± 52 | 751 ± 120 | 868 ± 42 | **0.006** | 0.233 | 0.916 |
| d13C.b.65 | -29.2 ± 0.1 | -29.0 ± 0.1 | -28.9 ± 0.6 | -29.9 ± 0.3 | -29.5 ± 0.2 | -28.7 ± 0.3 | -29.4 ± 0.3 | -29.8 ± 0.2 | 0.649 | **0.030** | 0.590 |
| d13C.s.65 | -27.8 ± 0.2^ab^ | -27.4 ± 0.2^a^ | -28.6 ± 0.2^ac^ | -29.0 ± 0.4^c^ | -28.2 ± 0.2^ac^ | -27.5 ± 0.3^a^ | -28.8 ± 0.07^bc^ | -29.0 ± 0.1^c^ | 0.365 | **<0.001** | 0.853 |
| d13C.p.65 | -25.0 ± 0.2^a^ | -25.7 ± 0.1^ab^ | -26.1 ± 0.06^bc^ | -27.4 ± 0.5^d^ | -25.9 ± 0.1^abc^ | -25.6 ± 0.2^ab^ | -26.5 ± 0.3^bd^ | -27.0 ± 0.2^cd^ | 0.290 | **<0.001** | 0.060 |
| d13C.a.65 | -27.1 ± 0.5 | -27.0 ± 0.0 | -27.9 ± 0.4 | -28.2 ± 0.5 | -27.2 ± 0.1 | -26.9 ± 0.2 | -27.9 ± 0.1 | -27.9 ± 0.5 | 0.689 | **0.016** | 0.928 |
| d13C.g.65 | -26.9 ± 0.4 | -26.7 ± 0.0 | -27.2 ± 0.6 | -28.1 ± 0.6 | -27.1 ± 0.3 | -26.8 ± 0.4 | -27.9 ± 0.2 | -28.0 ± 0.3 | 0.425 | **0.012** | 0.761 |
| d13C.l.65 | -27.1 ± 0.3 | -26.8 ± 0.2 | -26.9 ± 0.6 | -27.8 ± 0.4 | -27.1 ± 0.2 | -26.9 ± 0.3 | -27.7 ± 0.1 | -27.8 ± 0.0 | 0.312 | **0.031** | 0.547 |
| d13C.b.75 | -29.5 ± 0.0^ab^ | -29.0 ± 0.2^a^ | -29.3 ± 0.3^ab^ | -29.9 ± 0.2^b^ | -29.3 ± 0.1^ab^ | -29.3 ± 0.2^ab^ | -29.3 ± 0.1^ab^ | -29.9 ± 0.1^b^ | 0.970 | **0.003** | 0.415 |
| d13C.s.75 | -28.6 ± 0.3^ab^ | -27.9 ± 0.1^a^ | -28.3 ± 0.1^ab^ | -28.9 ± 0.3^ab^ | -28.4 ± 0.1^ab^ | -28.0 ± 0.4^a^ | -28.3 ± 0.1^ab^ | -29.1 ± 0.1^b^ | 0.906 | **0.001** | 0.884 |
| d13C.p.75 | -26.6 ± 0.2^ab^ | -26.2 ± 0.4^a^ | -26.3 ± 0.2^a^ | -27.9 ± 0.3^b^ | -26.3 ± 0.2^a^ | -26.3 ± 0.3^a^ | -26.4 ± 0.1^a^ | -27.3 ± 0.4^ab^ | 0.406 | **0.001** | 0.693 |
| d13C.a.75 | -27.4 ± 0.1^ab^ | -26.7 ± 0.4^ab^ | -26.5 ± 0.2^a^ | -28.1 ± 0.4^b^ | -27.3 ± 0.4^ab^ | -26.6 ± 0.3^a^ | -26.9 ± 0.3^ab^ | -27.9 ± 0.2^ab^ | 0.939 | **0.001** | 0.701 |
| d13C.g.75 | -27.9 ± 0.2 | -27.2 ± 0.4 | -26.9 ± 0.3 | -28.4 ± 0.5 | -28.0 ± 0.4 | -27.4 ± 0.2 | -27.8 ± 0.2 | -28.5 ± 0.3 | 0.177 | **0.008** | 0.548 |
| d13C.l.75 | -27.5 ± 0.1 | -27.0 ± 0.4 | -26.6 ± 0.3 | -28.0 ± 0.5 | -27.5 ± 0.3 | -27.2 ± 0.3 | -27.2 ± 0.1 | -27.8 ± 0.3 | 0.542 | **0.022** | 0.619 |
| d13C.grain.75 | -26.7 ± 0.2 | -26.2 ± 0.4 | -26.6 ± 0.1 | -27.1 ± 0.4 | -26.0 ± 0.3 | -26.2 ± 0.2 | -26.6 ± 0.2 | -26.8 ± 0.3 | 0.279 | 0.122 | 0.619 |
| d13C.grain.85 | -26.0 ± 0.1^ac^ | -25.7 ± 0.4^ab^ | -26.7 ± 0.3^bc^ | -26.7 ± 0.1^bc^ | -26.3 ± 0.2^ac^ | -25.4 ± 0.1^a^ | -26.2 ± 0.2^ac^ | -27.0 ± 0.2^c^ | 0.852 | **<0.001** | 0.273 |
| d13C.grain.92 | -25.8 ± 0.1^ac^ | -25.6 ± 0.4^ab^ | -26.5 ± 0.3^bc^ | -26.6 ± 0.1^bc^ | -26.1 ± 0.1^ac^ | -25.3 ± 0.1^a^ | -26.1 ± 0.2^ac^ | -26.8 ± 0.2^c^ | 0.726 | **<0.001** | 0.324 |
| d15N.b.65 | 1.38 ± 0.30 | 2.10 ± 0.24 | 1.98 ± 0.71 | 2.49 ± 0.14 | 1.87 ± 0.30 | 1.42 ± 0.33 | 2.21 ± 0.30 | 2.43 ± 0.37 | 0.991 | 0.150 | 0.457 |
| d15N.s.65 | 0.58 ± 0.41 | 1.46 ± 0.09 | 1.93 ± 0.65 | 1.57 ± 0.18 | 0.86 ± 0.33 | 0.54 ± 0.08 | 0.88 ± 0.34 | 0.93 ± 0.24 | **0.029** | 0.247 | 0.240 |
| d15N.p.65 | 1.64 ± 0.23 | 2.07 ± 0.33 | 2.07 ± 0.62 | 2.20 ± 0.44 | 1.02 ± 0.45 | 1.33 ± 0.30 | 1.47 ± 0.36 | 1.19 ± 0.42 | **0.021** | 0.708 | 0.954 |
| d15N.a.65 | 2.20 ± 0.21 | 3.03 ± 0.38 | 2.77 ± 0.46 | 2.41 ± 0.16 | 2.01 ± 0.30 | 1.70 ± 0.45 | 2.01 ± 0.56 | 2.01 ± 0.60 | **0.037** | 0.890 | 0.554 |
| d15N.g.65 | 1.71 ± 0.19 | 2.23 ± 0.56 | 1.87 ± 0.56 | 1.37 ± 0.10 | 1.62 ± 0.37 | 1.29 ± 0.40 | 1.34 ± 0.59 | 1.41 ± 0.40 | 0.230 | 0.849 | 0.662 |
| d15N.l.65 | 1.99 ± 0.18 | 2.45 ± 0.31 | 2.41 ± 0.41 | 1.54 ± 0.20 | 1.75 ± 0.31 | 1.19 ± 0.49 | 1.43 ± 0.47 | 1.59 ± 0.66 | 0.051 | 0.824 | 0.364 |
| d15N.b.75 | 1.74 ± 0.36 | 2.03 ± 0.24 | 2.23 ± 0.21 | 2.53 ± 0.24 | 1.39 ± 0.08 | 1.35 ± 0.44 | 1.60 ± 0.60 | 2.32 ± 0.09 | 0.063 | 0.082 | 0.869 |
| d15N.s.75 | 0.80 ± 0.31^ab^ | 0.99 ± 0.09^ab^ | 0.93 ± 0.07^ab^ | 1.51 ± 0.06^a^ | 0.18 ± 0.13^b^ | -0.01 ± 0.46^b^ | 0.33 ± 0.47^ab^ | 1.04 ± 0.14^ab^ | **0.003** | **0.029** | 0.782 |
| d15N.p.75 | 1.30 ± 0.31 | 1.16 ± 0.22 | 1.28 ± 0.08 | 1.33 ± 0.28 | 0.40 ± 0.09 | 0.42 ± 0.37 | 0.71 ± 0.39 | 0.61 ± 0.34 | **0.002** | 0.861 | 0.954 |
| d15N.a.75 | 2.35 ± 0.51 | 1.95 ± 0.12 | 2.13 ± 0.05 | 2.18 ± 0.34 | 1.47 ± 0.19 | 1.02 ± 0.27 | 1.76 ± 0.57 | 1.66 ± 0.08 | **0.009** | 0.445 | 0.784 |
| d15N.b.75 | 1.65 ± 0.61 | 1.40 ± 0.09 | 1.30 ± 0.10 | 1.63 ± 0.22 | 0.95 ± 0.43 | 0.34 ± 0.36 | 0.76 ± 0.49 | 1.04 ± 0.03 | **0.010** | 0.505 | 0.874 |
| d15N.l.75 | 1.88 ± 0.51^ab^ | 2.07 ± 0.23^a^ | 1.84 ± 0.16^ab^ | 2.18 ± 0.22^a^ | 0.38 ± 0.37^b^ | 0.23 ± 0.50^b^ | 1.23 ± 0.39^ab^ | 1.45 ± 0.02^ab^ | **<0.001** | 0.164 | 0.231 |
| d15N.grain75 | 4.32 ± 0.68^a^ | 3.68 ± 0.13^ab^ | 3.45 ± 0.19^ab^ | 3.49 ± 0.26^ab^ | 1.63 ± 0.59^b^ | 2.54 ± 0.43^ab^ | 2.32 ± 0.58^ab^ | 2.50 ± 0.21^ab^ | **<0.001** | 0.962 | 0.197 |
| d15N.grain.85 | 3.33 ± 0.37 | 2.87 ± 0.09 | 3.10 ± 0.78 | 2.38 ± 0.57 | 1.83 ± 0.46 | 1.57 ± 0.34 | 2.15 ± 0.76 | 2.38 ± 0.23 | **0.018** | 0.840 | 0.478 |
| d15N.grain.92 | 3.43 ± 0.38 | 3.01 ± 0.06 | 3.35 ± 0.80 | 2.61 ± 0.67 | 1.93 ± 0.47 | 1.71 ± 0.32 | 2.23 ± 0.79 | 2.65 ± 0.25 | **0.020** | 0.868 | 0.479 |

**Supplementary Figure 1.** Leaf chlorophyll, flavonols and anthocyanins contents and N balanced index (NBI) in four varieties of field-grown durum wheat (Kiko Nick, Don Ricardo, Euroduro and Haristide) at two N levels (control vs low N). Asterisks indicate a significant difference between varieties and N levels (*, *P*<0.05; **, *P*<0.01; ***, *P*<0.001) according to two-way ANOVA.


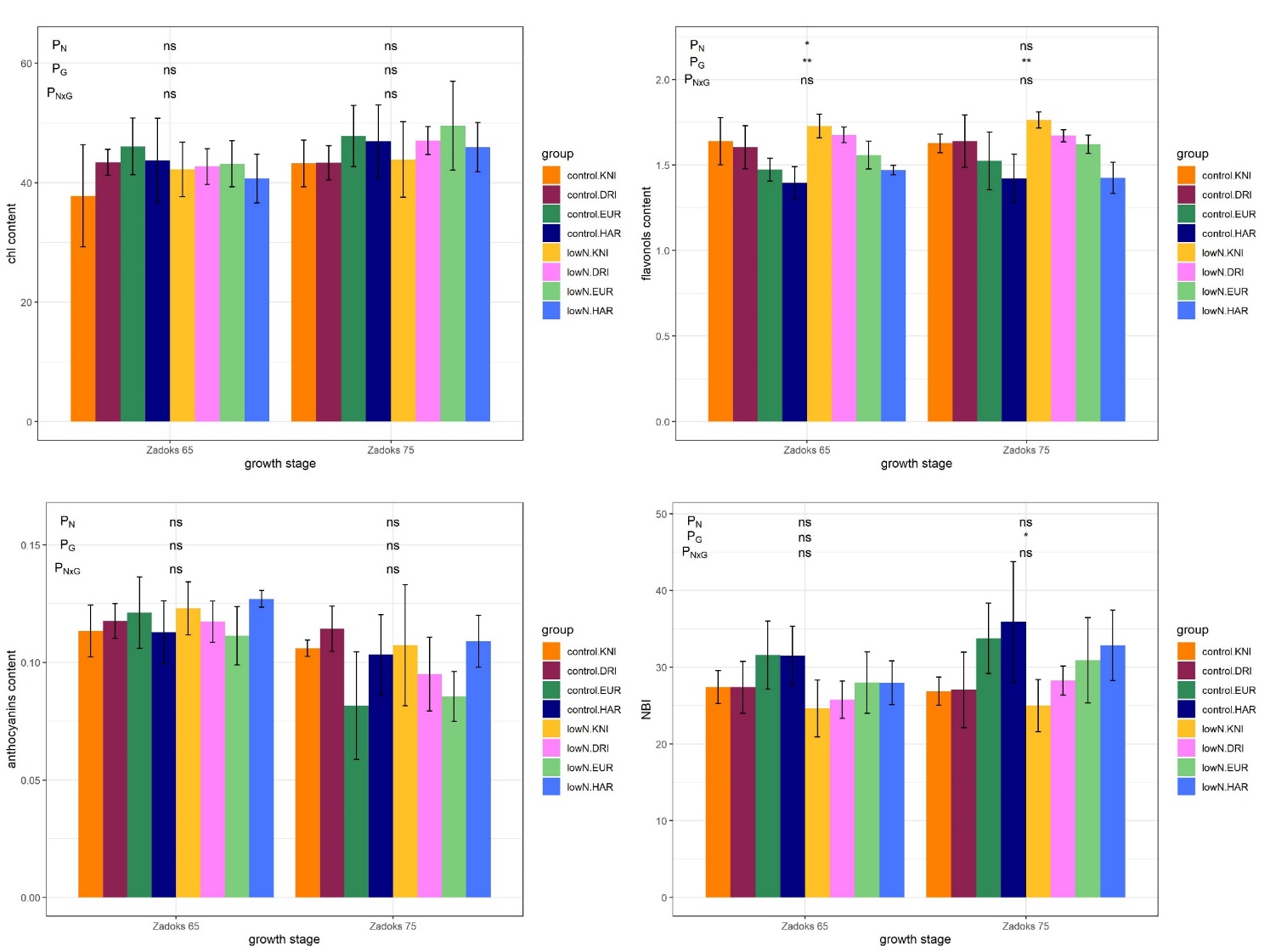


**Supplementary Figure 2.** Principal component analysis of metabolites, enzyme activities and nutrient composition in field-grown durum wheat per organ (blade, sheath, peduncle, awn, glume and lemma) at two N levels (control vs low N). The abbreviations are described in Supplementary Table 1.

**
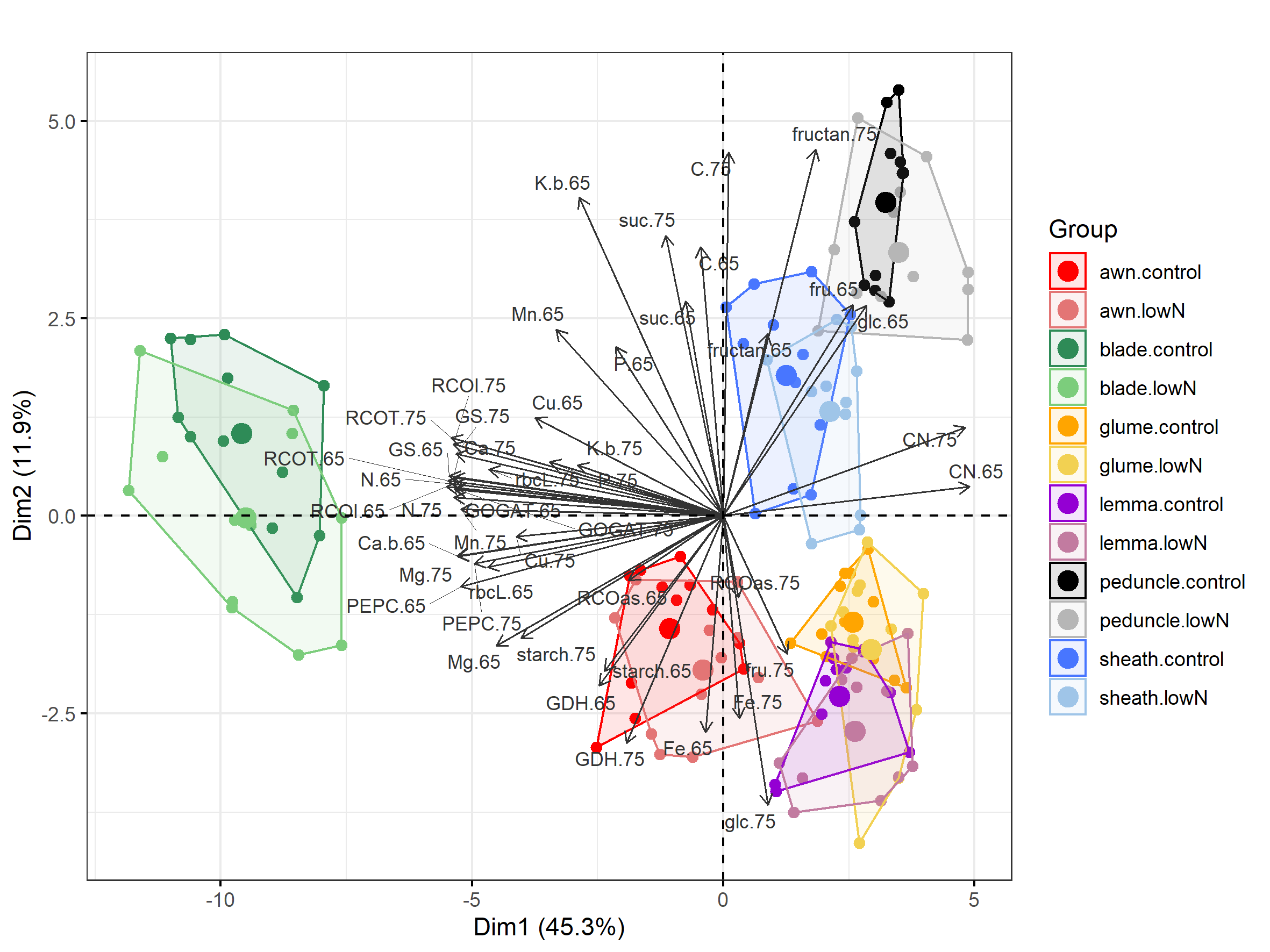
**

**Supplementary Figure 3.** Principal component analysis of metabolites, enzyme activities and nutrient composition in four varieties of field-grown durum wheat (Kiko Nick, Don Ricardo, Euroduro and Haristide) at two N levels (control vs low N) per organ (blade, sheath, peduncle, awn, glume and lemma). The abbreviations are described in Supplementary Table 1.

**
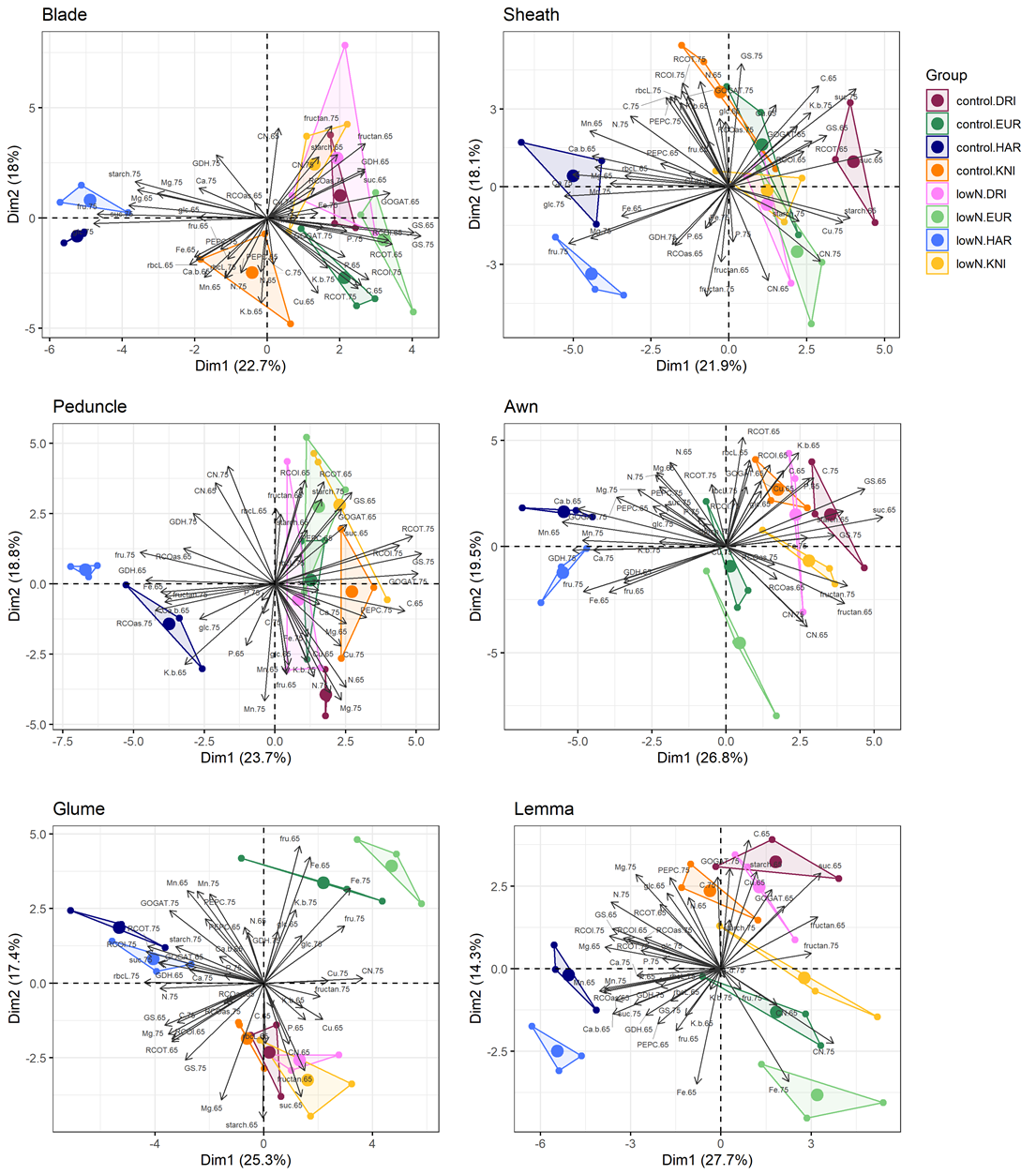
**
